# Expansion and subfunctionalisation of flavonoid 3',5'-hydroxylases in the grapevine lineage

**DOI:** 10.1186/1471-2164-11-562

**Published:** 2010-10-12

**Authors:** Luigi Falginella, Simone D Castellarin, Raffaele Testolin, Gregory A Gambetta, Michele Morgante, Gabriele Di Gaspero

**Affiliations:** 1 Dipartimento di Scienze Agrarie e Ambientali, University of Udine, via delle Scienze 208, 33100 Udine, Italy; 2 Istituto di Genomica Applicata, Parco Scientifico e Tecnologico Luigi Danieli, via Jacopo Linussio 51, 33100 Udine, Italy; 3 Department of Viticulture and Enology, University of California, 1 Shields Ave, Davis, CA 95616, USA

## Abstract

**Background:**

Flavonoid 3',5'-hydroxylases (F3'5'Hs) and flavonoid 3'-hydroxylases (F3'Hs) competitively control the synthesis of delphinidin and cyanidin, the precursors of blue and red anthocyanins. In most plants, *F3'5'H *genes are present in low-copy number, but in grapevine they are highly redundant.

**Results:**

The first increase in *F3'5'H *copy number occurred in the progenitor of the eudicot clade at the time of the γ triplication. Further proliferation of *F3'5'H*s has occurred in one of the paleologous loci after the separation of Vitaceae from other eurosids, giving rise to 15 paralogues within 650 kb. Twelve reside in 9 tandem blocks of ~35-55 kb that share 91-99% identity. The second paleologous *F3'5'H *has been maintained as an orphan gene in grapevines, and lacks orthologues in other plants. Duplicate *F3'5'H*s have spatially and temporally partitioned expression profiles in grapevine. The orphan *F3'5'H *copy is highly expressed in vegetative organs. More recent duplicate *F3'5'H*s are predominately expressed in berry skins. They differ only slightly in the coding region, but are distinguished in the structure of the promoter. Differences in *cis*-regulatory sequences of promoter regions are paralleled by temporal specialisation of gene transcription during fruit ripening. Variation in anthocyanin profiles consistently reflects changes in the *F3'5'H *mRNA pool across different cultivars. More *F3'5'H *copies are expressed at high levels in grapevine varieties with 93-94% of 3'5'-OH anthocyanins. In grapevines depleted in 3'5'-OH anthocyanins (15-45%), fewer *F3'5'H *copies are transcribed, and at lower levels. Conversely, only two copies of the gene encoding the competing F3'H enzyme are present in the grape genome; one copy is expressed in both vegetative and reproductive organs at comparable levels among cultivars, while the other is transcriptionally silent.

**Conclusions:**

These results suggest that expansion and subfunctionalisation of *F3'5'H*s have increased the complexity and diversification of the fruit colour phenotype among red grape varieties.

## Background

Flavonoid 3',5'-hydroxylases (F3'5'Hs) and flavonoid 3'-hydroxylases (F3'Hs) are versatile enzymes that accept several phenylpropanoid substrates [1]. Of particular interest for anthocyanin pigmentation is the 3',5'- or 3'-hydroxylation of naringenin and dihydrokaempferol. F3'5'Hs and F3'Hs compete for substrate recruitment and deliver their 3'5'- or 3'-OH products into the parallel synthesis of delphinidin and cyanidin [2], the precursors of blue and red anthocyanins in grape berries, respectively. Variation in anthocyanin profile within and between grape varieties is associated with differences in the ratio of *F3'5'H *to *F3'H *expression [3,4].

Anthocyanin biosynthesis takes place over 8-10 weeks, from shortly after berry softening (~60 days after blooming) until harvest [5]. *F3'H*s are expressed at comparable levels in both anthocyanin-pigmented and green-skinned varieties, before and after the onset of ripening [6,4]. However, regulation of *F3'5'H*s is largely genotype-specific and responsive to environmental cues [3,7]. The breadth of diversity in fruit colour among different grapevine accessions suggests a fine regulation of *F3'5'H *expression. Dark blue cultivars transcribe *F3'5'H*s at higher levels than light red cultivars, which nevertheless maintain traces of 3'5'-OH anthocyanins and barely detectable *F3'5'H *transcripts. In green-skinned cultivars, *F3'5'H *transcripts are completely absent [8,9]. The invariant presence of some 3'5'-OH anthocyanins in red pigmented grapes contrasts with many other flowering plants such as roses, carnations, chrysanthemums, lilies, gerbera, and *Arabidopsis*, which accumulate anthocyanins but do not synthesise 3'5'-OH derivatives.

The lack of grapevines with *F3'5'H *loss-of-function genotypes could be explained either by selection, which acted against knockout mutations, or by gene redundancy, which obscured the effect of single-gene loss/silencing. The observation that an absence of 3'5'-OH anthocyanins is generally tolerated in plants disfavours the first hypothesis. Furthermore, gene redundancy of *F3'5'H*s is commonplace in grape genomes [10,11], contrasting with most other species that have single or two-copy *F3'5'H*s, or none at all. We have previously shown that *F3'5'Hs *are highly duplicated, with multiple copies arrayed in clustered contigs of the 'Cabernet Sauvignon' physical map [10]. The genome assembly of the nearly-homozygous line PN40024 [12] allows a deeper investigation into the structure of the *F3'5'H *locus and into the evolutionary events that caused their proliferation in grapevine.

Expansion of gene families is common in plant genomes [13], and results from various mechanisms of duplication: whole-genome duplication (WGD), segmental duplication, tandem duplication, and transpositional duplication [14,15]. WGDs have repeatedly occurred over evolutionary time in the common ancestor of eudicots and in specific lineages [12,16]. Segmental duplications occur over chromosomal regions, which may undergo subsequent rearrangement. Tandem duplications generate nearby gene copies [13]. Small-scale duplications may also cause transposition of one of the duplicate genes to an ectopic site. In this paper, local duplications of small fragments (<10 kb) containing a single gene are referred to as *tandem duplications*. Duplication of DNA blocks >10 kb are referred to as *segmental duplications*.

Retention of duplicate genes results from a stochastic process, in which the effect of the earliest mutation occurring after duplication governs the fate of extra copies. Deleterious mutations occur much more frequently than mutations resulting in novel and favourable functions [17]. Following this assumption, gene disruption would largely prevail, with genomes populated by vestiges of ancient duplicates. This raises the question as to why intact duplicates are maintained and expressed much more frequently than expected by chance. According to the duplication-degeneration-complementation (DDC) model [18], degenerative mutations promote preservation of duplicate genes. Deleterious mutations in regulatory regions could eliminate different *cis*-elements in either duplicate, making both copies necessary to provide the full-complement of the expression profile of the ancestral single copy [19]. This kind of partitioned expression among duplicate genes is referred to as subfunctionalisation, and includes differential expression among organs and developmental stages, or in response to environmental cues [20-25].

Duplicate genes involved in secondary metabolism or that are responsive to environmental stimuli appear to be more frequently maintained [26-28], and have more highly diverged transcriptional patterns and intraspecific variation in expression [29] than duplicate genes in other categories. The pioneering study of [30] provided a paradigmatic case of duplication and transcriptional diversification in members of the stilbene synthase gene family in grapevine. It is generally assumed that maintenance of duplicate genes provides a foundation for consolidation and refinement of established functions, particularly in secondary metabolism, by preserving extra copies that guarantee a gene reservoir for adaptive evolution, free from the constraints of purifying selection [31-33].

In this paper, we present (i) the evolutionary path that led to the structural architecture of the *F3'5'H *gene family in grapevine, (ii) the transcriptional sub-functionalisation of duplicate copies among organs and developmental stages, and (iii) the extent of variation of expression patterns in four cultivars with divergent anthocyanin profiles.

## Results

### *F3'5'Hs *and *F3'Hs *in grapevine: genomic location and phylogeny

**Figure 1:**
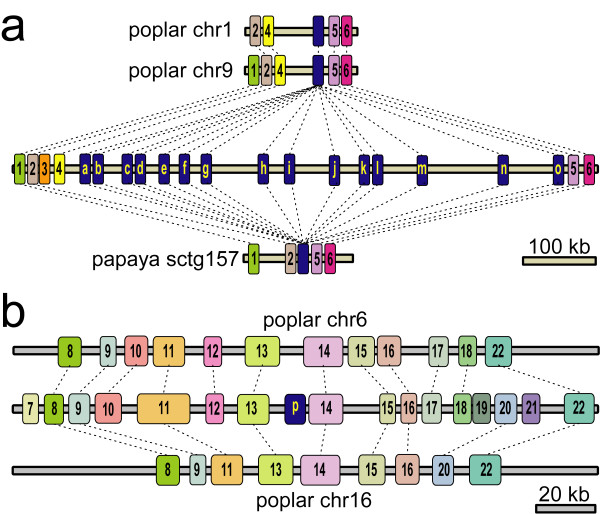
**Expansion of the *F3'5'H *locus in grapevine chr6, and expected position of the chr8 orphan *F3'5'Hp *in syntenic loci on poplar chr6 and 16**. Diagrams of gene colinearity between an 800-kb region containing arrayed *F3'5'H*s on grapevine chr6 (in the middle of **section a**), and syntenic regions in poplar (on top, chr1:19,850,000..20,000,000 and chr9:6,000,000..6,150,000) and in papaya (bottom, sctg157:250,000..400,000), and between 200 kb surrounding *F3'5'Hp *on grapevine chr8 (in the middle of **section b**) and syntenic loci in poplar. Blue boxes indicate *F3'5'H*s. Numbered boxes stand for genes encoding: (1) pentatricopeptide repeat-containing protein, (2) RNA recognition motif-containing protein, (3) histone mRNA exonuclease1, (4) dihydropyrimidinase, (5) calcium-dependent kinase, (6) rhodanese-like peptidyl-prolyl cis-trans isomerise, (7) zinc finger protein, (8) hypothetical protein, (9) DNA-binding protein, (10) alpha-glucosidase, (11) DNA gyrase subunit B, (12) UDP-glucuronosyltransferase, (13) microtubule-associated protein, (14) bZIP transcription factor; (15) hypothetical protein, (16) nudix hydrolase, (17) amino acid transporter, (18) hypothetical protein, (19) hypothetical protein, (20) phosphatidylinositol-4-phosphate 5-kinase, (21) cytokinin inducible protein, (22) glutamate synthase

Sixteen copies of *F3'5'H*s are present in the PN40024 genome. Each *F3'5'H *copy is referred to as *F3'5'Ha *through *F3'5'Hp*, with the alphabetical order reflecting their genomic coordinates [see Additional file 1]. Fifteen of them (*F3'5'Ha-o*) reside in a tandem array within a 650-kb region on chromosome (chr) 6. This chromosomal region is syntenic with the homoeologous chr1 and 9 in poplar, and with supercontig157 in papaya (Figure 1a). An isolated *F3'5'H *copy (*F3'5'Hp*) resides on grapevine chr8, a chromosome that was homoeologous to chr6 in the paleohexaploid ancestor [12]. However, other genes in a 100-kb interval around *F3'5'Hp *are single-copy, and not collinear with genes in the region on chr6 surrounding the other *F3'5'H*s [see Additional file 2]. *F3'5'Hp *is an orphan gene that lacks orthologues in other sequenced dicots and in EST databases. In poplar, one or both homoeologous loci syntenic with the grapevine *F3'5'Hp *region, which are present in the homoeologous chr6 and chr16 generated by the Salicoid WGD [34], have maintained the collinear genes present in grapevine, except for *F3'5'Hp *(Figure 1b).

Seven *F3'5'H*s on grapevine chr6 (*F3'5'Hd, -f, -j, -l, -m, -n, -o*) and *F3'5'Hp *on chr8 encode full-length proteins. In the haplotype of PN40024, the remainder gene models are either gene fragments without homology outside of conserved regions, or coding regions interrupted by transposable elements (TEs) or frameshift indels [see Additional file 3].

Grapevine contains two copies of *F3'H *(*F3'Ha *and *F3'Hb*) located in a 25-kb interval on chr17 [see Additional file 4a]. *F3'H*s reside in two blocks of ~5 kb, which share 93.5% identity over 4.3 kb of conserved sequence, separated by ~16 kb largely consisting of repetitive elements. Both *F3'H*s encode full-length proteins. *F3'Ha *and *F3'Hb *share 97% amino acid identity, but their genomic sequences differ extensively due to a large indel in the terminal intron [see Additional file 4]. Other genes surrounding the two *F3'H *copies on chr17 are not collinear with genes surrounding *F3'H*s on chr6 or on chr8.

**Figure 2:**
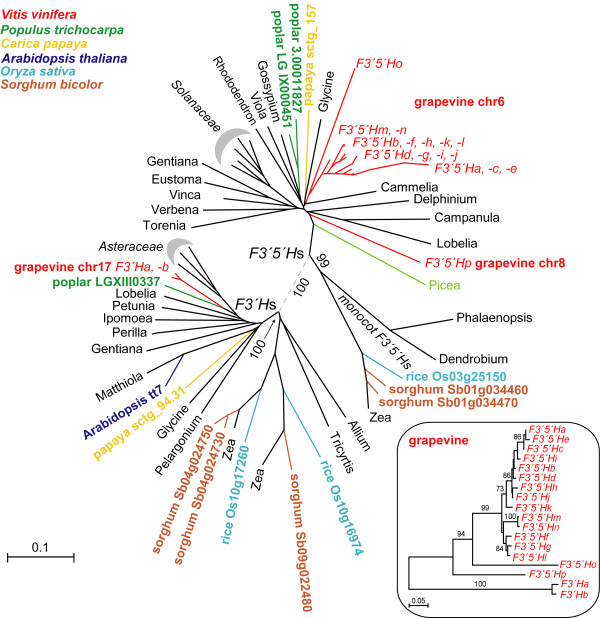
**Relatedness between *F3'5'H*s and *F3'H*s in completely sequenced genomes of six plant species (grapevine, poplar, papaya, Arabidopsis, rice, and sorghum) and in another 28 plants (indicated in the tree by the genus)**. The dashed grey branch connects the halves of the tree including either *F3'5'H*s (top) or *F3'H*s (bottom). A magnified view of the relatedness between grapevine *F3'5'H*s is given in the box. Bootstrapping was performed with 1,000 replicates. Percentage of replicates supporting each branch is given for major branches discussed in the text.

**Figure 3:**
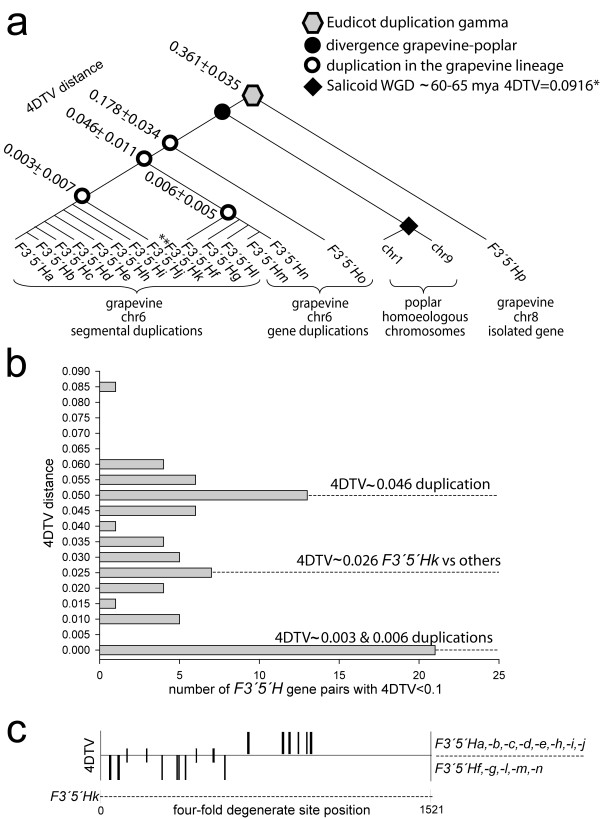
**Relative age of gene duplication using 4DTV as a proxy for time**. Branches are not drawn to scale. (**a**) 4DTV distance between grapevine *F3'5'H*s. (**b**) Distribution of 4DTV distance between the most recently duplicated *F3'5'H *pairs. (**c**) Nucleotide positions of 4DTV between the copy *F3'5'Hk *and two groups of the most recently duplicated *F3'5'H*s. Long ticks show variable positions within either group, short ticks show transversion with respect to both groups. * Estimated according to [34], ** intergenic recombination.

*F3'5'H *and *F3'H *gene phylogeny was analysed using translated sequences from six completely sequenced plant genomes and samples from other species, totalling 33 angiosperms and one gymnosperm (Figure 2). All *F3'5'H*s split from *F3'H*s. All grapevine *F3'5'H*s are highly conserved within the *F3'5'H *group. All of those located in the gene array on chr6 tightly group into a single major cluster. The more divergent *F3'5'Ho*, which resides at the distal side of the array on chr6, and the orphan *F3'5'Hp *on chr8 lie in deep-node branches (Figure 2). Subclades were identified within the major cluster based on maximum parsimony analysis of the coding sequences [see Additional file 5a]. Timing of divergence among duplicate *F3'5'H*s was estimated by four-fold synonymous third-codon transversion values (4DTV) (Figure 3a,b). The earliest duplication that gave rise to *F3'5'Hp *and the founder of all other *F3'5'H*s on chr6 occurred synchronously with the event of γ hexaploidisation (4DTV 0.361 ± 0.035). In the chr6 array, *F3'5'Ho *has extensively diverged from the progenitor of adjacent *F3'5'H*s, with 4DTV between gene pairs at 0.178 ± 0.034. Most of the recurrent duplications in the array have occurred much more recently, generating two groups of copies that diverged at 4DTV ~0.046 containing highly similar copies within each group (4DTV ~0.003-0.006). *F3'5'Hk *likely arose by illegitimate recombination between two paralogues that diverged at 4DTV ~0.046, as reflected by its intermediate 4DTV value (~0.026) and by the asymmetric distribution of 4DTV sites along *F3'5'Hk*, when compared with members of either group (Figure 3b,c).

The two copies of grapevine *F3'H *grouped tightly (Figure 2). *F3'H*s are consistently present in one or a few copies across fully sequenced plant species.

### Evolution of the *F3'5'H *locus on chromosome 6

The pattern and mode of gene duplication were characterised through several approaches: (i) dot plot self-comparison of the entire locus, (ii) conservation of non-coding sequences, TE patterns, and sequence divergence between long terminal repeats (LTRs) of retrotransposons in duplicate blocks, (iii) level of identity between 10-kb windows around each *F3'5'H*, (iv) intron divergence between the most recent duplicated *F3'5'H*s, and (v) conservation of duplicate *F3'5'H*s across the family Vitaceae.

**Figure 4:**
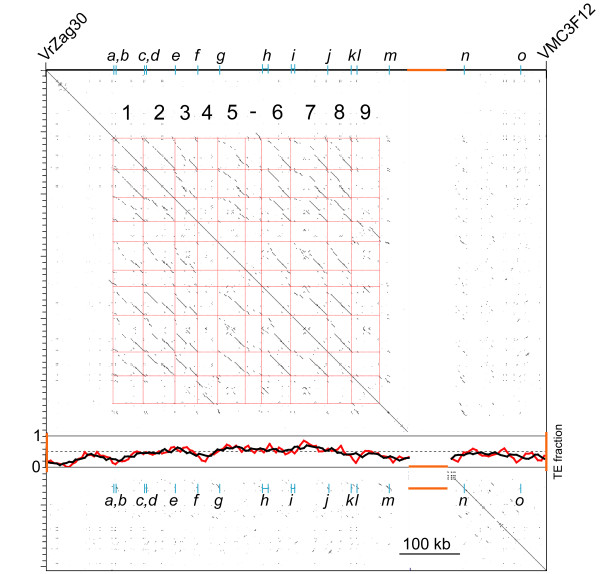
**Segmental duplications of 33-55 kb DNA blocks in the *F3'5'H *locus on grapevine chr6, delimited by genetic markers VrZag30 and VMC3F12**[10]. Dot plot self-comparison shows tandem duplications as linear arrays of dots in the same orientation as the main diagonal, but horizontally or vertically shifted off the main diagonal. The red grid delimits nine duplicate blocks. Cyan ticks indicate the position of 15 *F3'5'H*s. The fraction of TEs in a sliding window of 25 kb (red line) and 50 kb (black line) is shown in the graph superimposed onto the dot plot in the gap of the sequence assembly indicated by the orange bar.

**Figure 5:**
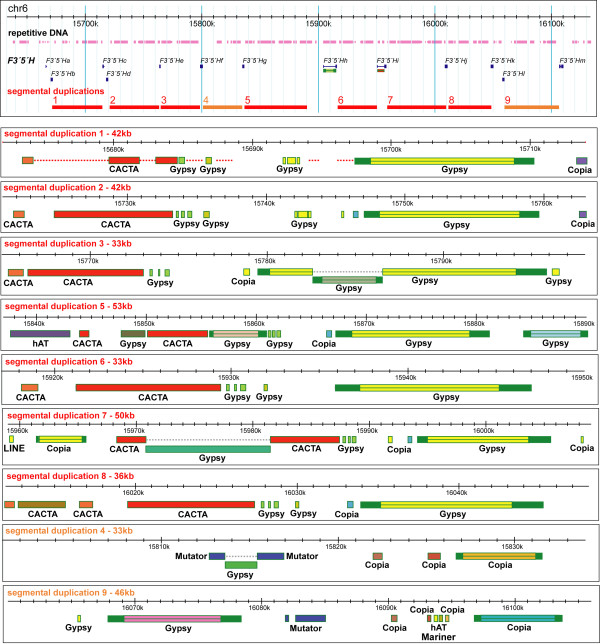
**Diagram of repetitive DNA and annotated TEs in segmental duplications within the *F3'5'H *locus on grapevine chr6**. Daughter copies of a TE present in an ancestral sequence and generated by a duplication of the block in which it resided are indicated with the same colour across blocks.

A dot plot self-comparison of the locus identified 9 blocks of DNA ranging in size from 35 to 55 kb, each containing one or two copies of *F3'5'H *at the forefront of the block (Figure 4). The remaining *F3'5'H *copies in this locus (*F3'5'Hm, F3'5'Hn*, and *F3'5'Ho*) are located downstream of the segmental duplications. Duplicated blocks do not contain genes other than *F3'5'H*s and are largely composed of repetitive DNA (Figure 4 and 5).

Blocks 1, 2, 3, 5, 6, 7, and 8 share 90-99% nucleotide identity, and each contain a CACTA and a *Gypsy *TE (Figure 5 and [see Additional file 6]). The ubiquitous presence of this *Gypsy *element across these blocks and the nucleotide substitution rate of 0.092 ± 0.023 between its LTRs date the *Gypsy *insertion to the ancestral single-copy sequence, recently in the evolutionary history of Vitaceae. The present-day block 6 is more reminiscent of the ancestral state of the sequence that initiated segmental duplications than blocks 1, 2, 3, 5, 7, and 8, as evidenced by the wide conservation of block 6 sequences among all of the other blocks, and by the fact that all of the other blocks resemble block 6 with various structural modifications. Blocks 1 and 2 resemble block 6 except for vestiges of a *Gypsy *element in the middle of the block. Block 3 is nearly identical to block 6, except for a recent *Gypsy *insertion into the shared *Gypsy *element. Sequence divergence between LTRs of this nested *Gypsy *is 0.003. Block 5 has undergone the most rearrangements, including *hAT *and *Gypsy *insertions at the extremities of the block, and two *Gypsy *invasions upstream and downstream of the proximal CACTA with low divergence between their LTRs (0.068). Block 7 has ~17 kb of extra DNA with respect to block 6 due to a *Copia *insertion and a nested *Gypsy *insertion into the shared CACTA. With respect to block 6, block 8 has an additional CACTA. Blocks 4 and 9 differ extensively from all other blocks and share 94.5% identity with each other (Figure 5 and [see Additional file 6]). A *Mutator *insertion predated the duplication of their common ancestor. In block 4, a *Gypsy *element has moved into the *Mutator *shared with block 9, and a *Copia *with 0.068 divergence between its LTRs has invaded the distal side. Block 9 was invaded by a *Gypsy *element with identical LTRs and by a *Copia *with 0.018 genetic distance between its LTRs.

Sequence conservation in a 10-kb window surrounding each *F3'5'H *copy supports the hypothesis that most of the copies were generated by duplications of the entire segment in which they reside [see Additional file 7], with the following exceptions. Downstream of the segmental duplications, sequence similarity between the nearly identical copies *F3'5'Hm *and *F3'5'Hn *does not extend more than ~ 2 kb beyond each side of their coding regions. *F3'5'Hk *and -*l *are both located upstream of block 9. *F3'5'Hl *and its 5' non-coding region are dissimilar from the paralogous *F3'5'H *in duplicate blocks 4 and 9, as though *F3'5'Hl *originated from a small scale duplication of *F3'5'Hg*, *-m*, or *-n*. *F3'5'Ho*, the copy at the far extremity of the locus, shares low similarity only upstream of the coding region with *F3'5'Ha*, -*b*, -*c*, -*d*, *-e*, and *-h*. *F3'5'Hp*, the copy on chr8, has no similarity outside of the coding region with other *F3'5'H*s.

Intronic sequences of highly similar paralogous *F3'5'H*s reflect the relatedness of the entirety of the duplicated block in which each *F3'5'H *resides [see Additional file 5b]. The few *F3'5'H*s that lie in pairs at the forefront of a duplicate block (*F3'5'Ha *and -*b*; *F3'5'Hc *and -*d*) are less similar within the pair than with a member of a different pair. Thus, paired *F3'5'H*s at the forefront of blocks 1 and 2 originated from an ectopic duplication before the duplication of the corresponding segment. The absence of intronless *F3'5'H*s excluded a role for retroposition in the process of gene duplication.

Conservation of duplicate *F3'5'H*s in the family Vitaceae was assayed by PCR with copy-specific primers. The orphan *F3'5'Hp *gene on chr8 was detected in the genera *Parthenocissus *and *Vitis*, while it was faintly amplified in *Ampelopsis*, likely due to more divergent priming sites [see Additional file 8]. In contrast, only a few primer pairs that amplified the most recent duplicate genes in *Vitis *genomes yielded amplicons in *Parthenocissus *or *Ampelopsis*. A wide sample of cultivars and species within the genus *Vitis *bears the marks of that expansion [see Additional file 8], including wine and table cultivars of *Vitis vinifera*, Asian and American *Vitis *species, and the muscadine grape.

### Prediction of functional domains among duplicate *F3'5'H*s

According to [35] and [36], six functional domains in the F3'5'H enzyme are important for the determination of substrate specificity and 3' vs. 3'5'-OH activity (substrate recognition sites, SRS; candidate region, CR1). *F3'5'Ha*, *-c, -e*, and *-h *are truncated in the PN40024 genome, and lack one or more functional domains [see Additional file 9]. All other grapevine *F3'5'H*s except *F3'5'Ho *have invariant amino acids specific for 3'5'-hydroxylation activity. In plants, *F3'5'H*s are conserved at three critical positions in the CR1 (positions 1, 3, and 10, which correspond to amino acids 178, 180, and 187 in the *Osteospermum F3'5'H *reference sequence used in [36]) and at two positions in the SRS6 (positions 5 and 8, which correspond to amino acids 484 and 487 in *Osteospermum F3'5'H*) [see Additional file 9]. All grapevine *F3'5'H*s that diverged less than 4DTV ~0.046 show complete amino acid conservation at the CR1 and SRS6 domains. *F3'5'Hp *on chr8 and *F3'5'Ho, -m*, and *-n*, the most divergent copies in the *F3'5'H *array on chr6, have a Met-to-Ile substitution at CR1 position 3 with respect to other paralogues. This substitution is shared with *F3'5'H*s in grasses. *F3'5'Ho *also has an Ala-to-Thr substitution at SRS6 position 8, which is shared with corn and sorghum *F3'5'H*s, as well as with most of the *F3'H*s. *F3'5'Hp *has an Ala-to-Val substitution at the same position, which is uniquely shared with *F3'5'H*s from orchids. *F3'5'Ho *has extensively diverged from all other *F3'5'H*s at SRS1 and SRS2, while *F3'5'Hp *has peculiar amino acid substitutions at SRS2, SRS4, and SRS5.

### Variation in promoter regions of duplicate *F3'5'H*s

**Figure 6:**
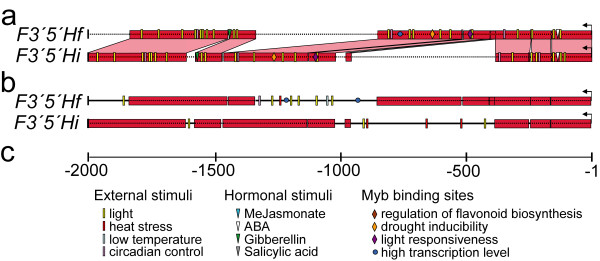
**Complementary evolution of 5' regulatory regions in duplicate *F3'5'H*s, within 2,000 bp upstream of the translation start site**. Red boxes in panels **a **and **b **indicate conserved regions, which were aligned using the DiAlign2 algorithm implemented in GEvo, and linked in this figure by pink connectors between the two paralogues. Regulatory DNA stretches uniquely present in either duplicate are represented as horizontal dotted lines. Putative DNA binding motifs were identified using PlantCARE, and are shown in panel **a **if they occurred in regulatory modules conserved between the two duplicate genes or in panel **b **if they occurred in regions that distinguished either duplicate. The scale of bp distance from the translation start site and the legend for twelve categories of DNA binding sites are given in panel **c**.

Duplicate *F3'5'H*s have originated from segmental duplications of large DNA blocks, which included the coding sequences and several kilobases of the surrounding DNA. In some cases, reorganisation of promoter regions within 2-kb upstream of the start codon occurred via TE insertion, for example *Copia *and hAT elements in the common ancestor of the present-day *F3'5'Hc *and *-e *duplicates. In other cases (Figure 6), structural variation in the promoter was caused by insertions/deletions of DNA segments of variable length up to a few hundred nucleotides, which do not belong to any annotated class of repetitive elements. These inserted/deleted portions are neither detected by algorithms of repetitive DNA search such as ReAS, nor are they duplicated elsewhere in the genome based on blastN searches. Structural variation in the promoters of *F3'5'H*s often occurred in a complementary fashion among gene copies, with a segment of one promoter having been lost in one duplicate but maintained in another, and vice versa. Comparison among triplets of promoters indicated that those segments were more often conserved in two *F3'5'H*s and absent from the third one than vice versa. All of this evidence excludes a mechanism of copy-and-paste insertion in the promoter of either duplicate gene, and favours the alternative hypothesis that structural deletions in the promoters of daughter copies have progressively degenerated the original sequence of the ancestral single-copy gene, partitioning the full complement of the regulatory information among copies.

Deletions may have asymmetrically erased *cis*-elements from regulatory regions of duplicate *F3'5'H*s. Thus, the 2-kb promoter regions of duplicate *F3'5'H*s were searched for DNA-binding motifs (Figure 6). Segments that were alternatively maintained in either promoter contained binding sites for Myb-type transcription factors, light-responsive and drought-inducible *cis*-elements, motifs sensitive to ABA and methyl-jasmonate, and heat stress responsive motifs. Relatedness between the alignable regions of duplicate promoters was also evident from a phylogenetic tree [see Additional file 5c].

### Spatial expression patterns of duplicate *F3'5'H*s and *F3'H*s

Expression analyses were conducted on nine out of the sixteen *F3'5'H *copies for which primer pairs could individually distinguish each paralogue and that passed the thresholds of PCR efficiency as set in the Methods section.

**Figure 7:**
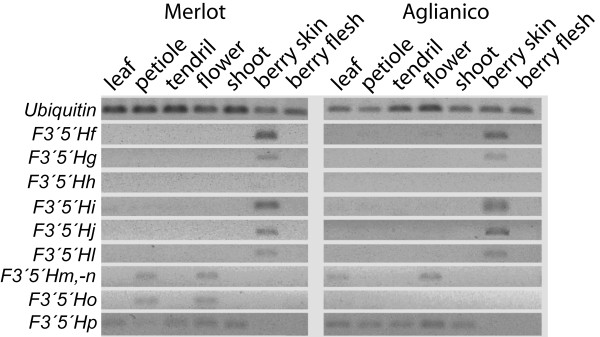
**Expression of duplicate *F3'5'H*s in organs of two grapevine cultivars**. Semiquantitative Q-PCR was performed upon cDNA normalisation with the housekeeping *Ubiquitin *gene.

Duplicate *F3'5'H*s are asymmetrically expressed across organs (Figure 7) [see Additional file 10]. The orphan copy *F3'5'Hp *is highly expressed in all vegetative organs (leaf, petiole, tendril, flower, and shoot) and very weakly in fruit. The highly duplicated *F3'5'H*s that reside in segmental duplications on chr6 are preferentially expressed in berry skin. Expression of *F3'5'Hm*, *-n*, and *-o*, three copies located outside of the segmentally duplicated region on chr6, was detectable in some vegetative organs, but not in berry skin during ripening in all cultivars tested [see Additional file 11]. In fruit, none of the *F3'5'H*s that are expressed in cultivars accumulating anthocyanins ('Aglianico', 'Marzemino', 'Grignolino', and 'Nebbiolo') are expressed during ripening in the green-skinned cultivar 'Tocai' (data not shown).

*F3'Ha *is widely expressed in many organs [see Additional file 4b]. In berry skins, *F3'Ha *expression increased 2-fold at full veraison, and then remained constant during the later stages of ripening [see Additional file 4d]. Transcripts of *F3'Hb *were never detected in the organs analysed in this study [see Additional file 4c] and weak expression of this copy was detected exclusively in adventitious roots of 'Cabernet Sauvignon' [37].

### Expression of the *F3'5'H *gene family and variation of anthocyanin profiles across different cultivars

Berries of four cultivars were sampled at eight developmental stages in order to quantify cumulative expression of the *F3'5'H *gene family and relative contribution of individual *F3'5'H *copies, and to determine anthocyanin profiles. The accessions 'Aglianico', 'Grignolino', 'Marzemino', and 'Nebbiolo' were chosen for their contrasting phenotypes of fruit colour, based on literature reports [4,9].

As a whole, expression of the *F3'5'H *gene family levelled off before veraison [see Additional file 12], in step with other genes of the flavonoid pathway [5]. *F3'5'H*s became increasingly more expressed at 10% veraison, peaking at full-veraison and ten days after full-veraison. Expression then declined two weeks before harvest and at harvest, but remained at higher levels than those detected before the onset of ripening.

**Figure 8:**
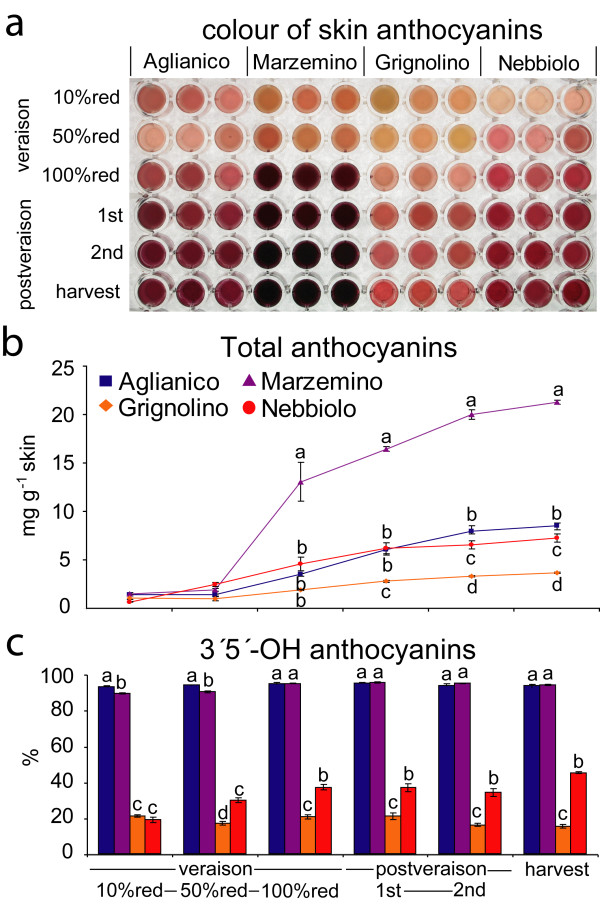
**Evolution of anthocyanin profile and colour in ripening fruit of four cultivars**. (**a**) Anthocyanins extracted from berry skin in a 1:1 methanol:water solution, (**b**) total anthocyanin content, and (**c**) percentage of 3'5'-OH anthocyanins in four cultivars at six ripening stages. Bars represent standard error of three biological replicates, shown for each cultivar in plate columns of **section a**. Differences among cultivars at each sampling date were tested for significance by one-way ANOVA. Means were separated by a Student-Newman-Keuls test, and significant differences at *P *< 0.05 are indicated by different letters. Stacked panels **b **and **c **share the same *x*-axis, reported below the bottom panel.

Cumulative expression of all duplicate *F3'5'H*s indicated that the cultivar 'Aglianico' had significantly greater *F3'5'H *expression during ripening than other cultivars. Cumulative *F3'5'H *expression in 'Aglianico' was 3-fold higher than in 'Marzemino', and almost 20-fold higher than in 'Grignolino' and 'Nebbiolo'. 'Aglianico' and 'Marzemino' yielded dark grape skin extracts (Figure 8a), with the highest concentrations of anthocyanins (Figure 8b), and their anthocyanin profiles were predominantly composed of 3'5'-OH anthocyanins (93-94%) (Figure 8c). 'Grignolino' and 'Nebbiolo' produced reddish skin extracts, with anthocyanin profiles depleted in 3'5'-OH anthocyanins (15% and 45%, respectively).

**Figure 9:**
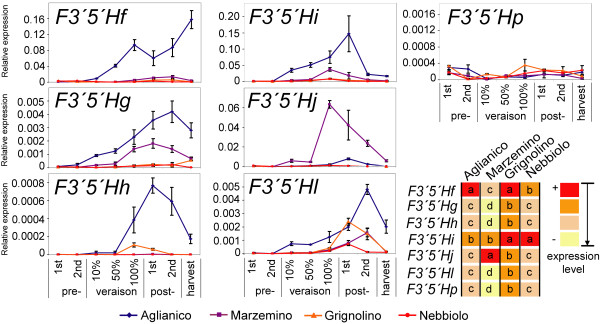
**Expression of duplicate *F3'5'H*s in berry skin of four cultivars across eight developmental stages**. Transcript levels are normalised to the expression of *Ubiquitin*. Bars indicate standard error of three biological replicates. Stacked panels share the same *x*-axis, reported below the bottom panel. The panel at the bottom right-hand corner reports significant differences between relative abundance of copy-specific transcripts across all developmental stages, within each cultivar. Colour scale from red to yellow indicates decreasing levels of gene expression, separated by a Student-Newman-Keuls test. Different letters indicate significant differences (*P *< 0.05).

The level of expression of every *F3'5'H *copy was highly variable in berry skin of different cultivars (Figure 9). As a result, the contribution of individual gene copies to the *F3'5'H *transcript pool was unique to each cultivar. PCR efficiency differences across cultivars are inherent when dealing with four heterozygous grapevine accessions of unrelated pedigree, due to possible nucleotide divergence across the eight haplotypes. For each *F3'5'H *primer pair we assessed that the standard deviation of PCR efficiency among cultivars is less than 10%, and it is therefore unlikely to explain these results. A two-way ANOVA identified significant differences in relative transcript levels among duplicate *F3'5'H*s within each cultivar. *F3'5'Hf *was the predominately expressed copy in 'Aglianico'. PCR efficiency for this copy in 'Aglianico' was 96.2%, which is within the bounds of the standard deviation of the average PCR efficiency of this gene family in the same cultivar (92.9% ± 4.6%). *F3'5'Hi *was the predominately expressed copy in 'Nebbiolo', and also in 'Grignolino' together with *F3'5'Hf*. In contrast, *F3'5'Hj *expression predominated in 'Marzemino'. *F3'5'Hg*, *-h*, *-l*, and *-p *were consistently expressed at lower levels across all cultivars, despite the observation that PCR efficiencies of their primer pairs were not lower than other *F3'5'H *copies in the accessions under study. Traces of transcripts of the copies *F3'5'Hm*, *-n*, and *-o *were never detected in the preliminary semiquantitative PCR screening at any stage of berry ripening in any of the accessions tested, even when PCR products were stained with silver nitrate for high sensitivity. Thus, they were excluded from further investigation by qPCR.

A three-way ANOVA was used to decouple and test the significance of three factors that contributed to the observed variation of expression patterns: gene-copy, cultivar, and developmental stage [see Additional file 12]. All three factors were significant, as well as the interactions: gene-copy × developmental stage, gene-copy × cultivar, cultivar × developmental stage, and gene-copy × cultivar × developmental stage (*P *< 0.00001).

### Distinct temporal expression patterns of duplicate *F3'5'H*s during ripening

**Figure 10:**
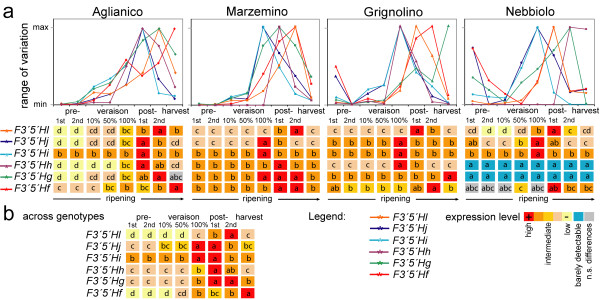
**Expression divergence of duplicate *F3'5'H*s during ripening**. (**a**) Transcript levels of each *F3'5'H *are expressed relative to the maximum peak of expression of that gene-copy in that cultivar. Different letters indicate significant differences in relative abundance of copy-specific transcripts among developmental stages. (**b) **Significant differences in relative abundance of copy-specific transcripts between developmental stages, regardless of the cultivar effect. In the diagram at the bottom of **section a**, and in **section b**, colour scale from red to cyan indicates decreasing levels of gene expression during ripening, separated by a Student-Newman-Keuls test. Different letters indicate significant differences (*P *< 0.05).

Individual gene copies were differentially regulated during ripening. Differences in the expression pattern of individual *F3'5'H*s with regard to developmental time were statistically significant in each of the four varieties, separately analysed by one-way ANOVA and when averaged across cultivars (Figure 10). *F3'5'Hi *and *-j *were expressed early, and attained a peak of expression between full-veraison and ten days post-veraison, consistently among cultivars. Late in ripening, *F3'5'H *expression was predominated by transcripts of *F3'5'Hf, -g ,-h*, and *-l*.

## Discussion

### Expansion of the *F3'5'H *family in grapevine

Gene-copy number of *F3'5'H*s has increased in the grapevine lineage through recurrent cycles of duplication. The most ancient duplication resulted in two *F3'5'H *loci. One of these, *F3'5'Hp*, has been maintained as a single-copy gene on chr8 in grapevine and other Vitaceae but lost from other dicot genomes. The other was the founder of the present-day *F3'5'H *gene array on chr6, orthologous to the *F3'5'H*s expressed in other dicot species and syntenic with the *F3'5'H *loci found in poplar and papaya (Figure 1). The 4DTV distance between *F3'5'Hp *and other *F3'5'H *copies is close to the peak of 4DTV distances between grape paleologues observed by Tang and coworkers [16] (Figure 3). Timing of the earliest *F3'5'H *duplication is therefore coincident with the event of eudicot γ hexaploidy [16], and the chromosomes in which the duplicate genes reside are indeed paleologous chromosomes [12].

The orphan copy *F3'5'Hp *is predominantly expressed in grape vegetative organs, in contrast with the *F3'5'H *copies on chr6, which are predominantly expressed in fruit (Figure 7). Several amino acid substitutions in *F3'5'Hp *are shared with *F3'H*s and monocot *F3'5'H*s. For instance, *F3'5'H*s are present in many monocot species, but in all cases studied, their transcription is uncoupled from the expression of other genes in the anthocyanin pathway. As a result monocots seldom accumulate 3'5'-OH anthocyanins [38]. For example, seed coats of rice varieties with dark red pigmentation contain exclusively 3'-OH anthocyanins, and the same holds true for sorghum and purple corn. 3'5'-OH anthocyanins are also absent in blue flowers of *Dendrobium *and *Phalaenopsis *orchids, albeit the detection of 3'5'-OH flavonols provides evidence for *F3'5'H *activity [39].

Expansion of *F3'5'H*s on chr6 occurred in the Vitaceae lineage after the separation from other dicots. Indeed, *F3'5'H *genes are present in low copy number in other fully sequenced plant genomes, if not lost. *F3'5'H *is absent from *Arabidopsis*, single-copy in rice and papaya, and dual-copy in poplar and sorghum. In poplar, the two copies of *F3'5'H *were generated by the Salicoid WGD [34]. The presence of a single-copy gene in the syntenic locus of poplar and papaya (Figure 1), and molecular dating of grapevine paralogues favour the hypothesis of lineage-specific gene duplications. The estimated age of *F3'5'H *duplications based on transversion rate at four-fold synonymous third-codon positions predicts most duplicate copies having diverged by less than 4DTV ~0.046 (Figure 3). If the molecular clock in grape is approximately calibrated by comparing the evolutionary rates in perennial dicots, the 4DTV distance of ~0.046 in grape is roughly half of the median 4DTV distance (~0.091) observed in poplar between duplicate genes that arose from the 60-65 myr-old Salicoid duplication [34]. However, grape has evolved more slowly than poplar, and the distances between paleologous genes that arose from the γ triplication are lower in grape (median *Ks*, 1.22) than in poplar (median *Ks*, 1.54), as estimated by [16]. Thus, recalibrating the mutation rate in grape, the 4DTV distances between *F3'5'H *in the chr6 array suggest that most duplications occurred within the past ~40 myr.

Molecular dating based on rate of nucleotide divergence is consistent with the conservation of duplicate gene copies across lineages in the family Vitaceae. While most of the 4DTV ~0.046 copies are conserved among *Vitis *species, they failed to be amplified from the DNA of related genera *Ampelopsis *and *Parthenocissus*. Conversely, the paleologous *F3'5'Hp *was conserved among these genera. Fossil records from the Late Cretaceous dates the radiation of *Vitis*, *Ampelopsis*, and *Parthenocissus *genera back to ~65 mya [40], confirming that most of the *F3'5'H *expansion occurred in an ancestor of the *Vitis *lineage, after the separation from the related lineages *Ampelopsis *and *Parthenocissus*.

The founder of the array of *F3'5'H*s on chr6 was initially duplicated through tandem gene duplication. Subsequently, different *F3'5'H *copies were involved in reiterated segmental duplications of large DNA blocks in which they resided, generating 9 blocks that range in size from ~35 to 55 kb (Figure 4 and 5). This modular structure suggests that unequal crossing-over between mispaired blocks was the most likely force that shaped the locus. Subsequent reorganisation via TE insertion, deletion, etc., resulted in structural variation among blocks, which might have reduced illegitimate recombination between adjacent blocks, thus resulting in the maintenance of the number of duplicates within the current bounds. Although our data suggest that most of the *F3'5'H *copies are maintained across grape varieties, at least in a heterozygous state, the extent of structural variation among haplotypes remains to be determined.

### Regulatory diversification within the *F3'5'H *family and anthocyanin profiles

Transcriptional subfunctionalisation has widely occurred within the *F3'5'H *family and is detectable even between some of the most recent duplicates that diverged less than 4DTV ~0.046. This is evident, for instance, among *F3'5'Hf*, *-j*, and *-l*, which have retained >94% amino acid identity, and among *F3'5'Hf*, *-g*, and *-l*, which show conservation at the CR1 and SRS6 domains for 3'5'-OH activity. Transcriptional subfunctionalisation is therefore one of the forces, if not the predominant one, that is responsible for the retention of the most recent duplicate *F3'5'H*s in grapevine. The extensive structural variation found in their 5' regulatory region, and the observed partitioned expression among organs and developmental stages might have promoted the diversification of duplicates shortly after their origination, and thus the preservation of both duplicates. These pieces of evidence fit well into the DDC model. Deletion of regulatory modules is expected to occur by chance in promoters of duplicate genes, eliminating different *cis*-elements in either duplicate and diversifying their expression profiles [18].

Alternatively, a gene dosage model may also explain retention of duplicate *F3'5'H*s [41], under the assumption that a fitness advantage is provided by extra *F3'5'H *copies. *F3'5'H *gene products compete with *F3'H *gene products for the enzymatic transformation of flavonoid substrates into delphinidin or cyanidin precursors. Copy number variation is a common cause of altered stoichiometry of concerted enzyme activities within metabolic pathways, which results in phenotypic variation [42]. Unbalanced phenotypes with increased levels of 3'5'-OH anthocyanins might have increased fitness, due to dissipation of high-energy blue wavelengths, attenuation of UV-B radiation, or conspicuousness of fruits to seed dispersers [43-46].

Regulatory modules alternatively maintained in the promoter of either *F3'5'H *duplicate contain binding sites for *Myb*-type transcription factors, drought-inducible *cis*-elements, and motifs responsive to ABA, methyl-jasmonate, light, and heat stress (Figure 6). The nature of these putative *cis*-elements correlates well with those factors shown to regulate *F3'5'H *expression. *Myb*-type transcription factors are activators of anthocyanin biosynthetic genes, including *F3'5'H*s [47-50]. Light and water deficits promote *F3'5'H *expression in the grape berry [3,7]. ABA and methyl-jasmonate are sucrose-dependent inducers of anthocyanin biosynthetic genes [51-53]. High temperatures restrict anthocyanin accumulation by promoting pigment degradation and transcriptional repression of anthocyanin genes [54,55].

Transcriptional regulation of duplicate *F3'5'H*s in berry skin is largely dependent on genotype, consistent with the observation in other plants that tandem duplicates have highly variable expression patterns [29]. In the present work, differential expression within the *F3'5'H *gene family between different cultivars was associated with the differential accumulation of 3'5'-OH anthocyanins. In the field, *F3'5'H *gene expression has a functional impact on anthocyanin biosynthesis that persists during fruit ripening. Different copies of duplicate *F3'5'H*s have also become temporally specialised for different developmental stages of berry ripening (Figure 10). The question remains as to why these nuanced expression patterns have been maintained evolutionarily. One hypothesis is that copy-specific *cis*-elements confer unique, adaptive patterns of expression and environmental responsiveness by increasing the ratio of F3'5'H/F3'H enzyme concentration (and thus 3'5'-OH anthocyanins) under circumstances when accumulation of this class of metabolites is advantageous.

## Conclusions

Expansion in copy-number and transcriptional specialisation of *F3'5'Hs *have increased the regulatory complexity of anthocyanin biosynthesis and fruit colour among red grape varieties. Most duplications occurred rather recently within this gene family, long after the Vitaceae lineage had separated from other dicot lineages. Among duplicate copies, accumulation of structural variation in promoter regions was more significant than divergence in coding regions. Transcriptional subfunctionalisation across organs and along developmental stages in ripening fruit was commonplace among gene copies, in addition to the extensive variation in gene expression among different cultivars. Transcriptional differences within the *F3'5'H *gene family in different accessions were paralleled by significant changes in the major metabolites synthesised by the *F3'5'H *gene products. In berry skin, the abundance of different anthocyanins that modulate the pigmentation of red grapes and wines was greatly affected by these transcriptional variations.

## Methods

### Sequence analyses

*F3'5'Hs *and *F3'Hs *were identified in grapevine (on chr6, chr8, and chr17 sequence assemblies deposited under the NCBI accession no. FN597024, FN597027, FN597042 as of 25 November 2009), poplar (version 1.0, [34]), *Arabidopsis*, rice, papaya, and sorghum (version of the genome assemblies available at Phytozome [56] as of November 2009) by tBlastN homology, using cytochrome P450 monooxygenases of the CYP75A subfamily (accession no. AAP31058, AB078781, AJ011862, Z22544, BAA03439, BAA03440) and the CYP75B sub-family (AY117551, BAD00189, AF155332) as a query. Matches were retained at thresholds of E<e^-20 ^and amino acid identity >50%. Each sequence was extended on each side until the next gene and annotated using GenScan, FgenesH, GeneMark, and Geneid. Sequence alignments were carried out using ClustalX. Exon-intron structure was predicted by comparison with ESTs and amino acid sequences from other plants. Trees were constructed using MEGA. Nucleotide substitution rate was calculated using DNAsp 4.0. 4DTV values were calculated and corrected for possible multiple transversions according to [16]. Gene models other than *F3'(5')H *were given the predicted function of their best match in the NCBI protein database. Syntenic regions were identified using the Genome Evolution tool [57]. Transposable elements were annotated according to the grape genome browser information [58]. LTRs in *Copia *and *Gypsy *retrotransposons were identified by dot plot analysis. Global DNA alignments of chromosomal segments were performed using LAGAN [59] in a window of 100 bp with a minimum identity of 70%. Dot plots of segmental duplications were made using Dotter. Alignments of 2-kb promoter regions were performed with DiAlign2, using a minimum HSP length of 10 bp and visualised with GEvo. DNA binding motifs were predicted by PlantCARE [60].

### Selective amplification of *F3'5'Hs *and *F3'Hs *paralogues

Selective primers were designed across dissimilar exonic DNA stretches or using a 3'-terminal SNP between the perfect match of the target gene-copy and the mismatched annealing site of paralogous sequences [see Additional file 13]. Absence of illegitimate cross-amplification of other paralogues was validated by amplification of genomic DNA, Sanger sequencing of the PCR products, and detection of variable sites inside of primer sequences that distinguished the target gene-copy from other paralogues. qPCR efficiencies in amplifying the DNA of PN40024 (from whose genome sequence gene-copy specific primers were designed) and of the mixed haplotypes of every heterozygous cultivar used in the present study were calculated using the equation E = 10^-1/slope ^of the standard curve. The standard curve was constructed with five 10-fold serial dilutions, using cDNA from organs and developmental stages in which the specific gene-copy was expressed or, if not possible, genomic DNA. Paralogue-specific primers with a PCR efficiency comprised between 90 and 110% in PN40024 were considered acceptable, and were used for qPCR if the standard deviation of their PCR efficiencies among the accessions under study was less than 10%. PCR primers that distinguished individual paleologous copies, as well as highly similar paralogues, and passed the thresholds set for the qPCR experiment, could be developed for nine out of the sixteen *F3'5'H *copies. The remaining copies were either highly identical in sequence or contained only a few polymorphic sites within DNA segments unsuitable for primer design. The range of variation in average PCR efficiency of primer pairs among the accessions tested was within the bounds of 87% in 'Marzemino' and 102% in 'Nebbiolo', with a similar average efficiency of 93% in 'Aglianico' and 'Grignolino'. This excluded a substantial cultivar effect of the efficiency of primer annealing during qPCR on the estimation of transcript levels of the whole gene family among cultivars, caused by possible SNPs in the annealing sites across haplotypes.

### Experimental design and statistics in expression and metabolite analyses

Variation in anthocyanin profile and in transcriptional level of duplicate genes among developmental stages and cultivars was studied using a complete randomized design, and tested for significance using ANOVA run by COSTAT statistical package (CoHort Software, Monterey, CA, USA). Each plot consisted of 10-in-a-row clonally replicated plants in north-south oriented rows.

Vines were grown at the germplasm repository of Vivai Cooperativi Rauscedo, northeastern Italy (46°04' N; 12°50' E; 110 masl). Vines were trained using the Sylvoz system. Three biological replicates of 20 berries per cultivar were collected at each developmental stage [see Additional file 14]. Berries of each replicate were collected in the vineyard on both sides of canopy by random sampling on every plant within each plot. Samples were frozen immediately in liquid nitrogen and stored at -80°C until processed. Skin of each biological replicate was peeled from frozen berries, powdered in liquid nitrogen, and split to obtain a 100 mg aliquot for RNA extraction and a 200 mg aliquot for anthocyanin extraction. A three-way ANOVA was used to partition the factors that contributed to expression divergence in ripening fruit: gene-copy, cultivar and developmental stage, and their interactions. A two-way ANOVA was used to assess the effect of gene-copy and developmental stage on expression level, regardless of the cultivar. A one-way ANOVA was used to assess the same effect in each cultivar, as well as the differences in metabolite content and composition among cultivars. Statistically significant differences were determined using the Student-Newman-Keuls test (*P *< 0.05).

### Anthocyanin profiling

Anthocyanins were extracted by sonication of 200 mg berry skin in 1.8 mL of 1:1 methanol-H_2_O for 30 minutes. After centrifugation at 13,000 × *g *for 15 min, samples were filtered with a 0.2 μm cellulose membrane (Phenomenex, Inc., Torrance, CA, USA). Anthocyanins were separated by an Agilent 1200 Series HPLC system (Agilent Technologies, Inc., Santa Clara, CA, USA) equipped with a C18 Purospher RP-18 (5 mm, 250 × 4 mm) column (Merck, Darmstadt, Germany), according to the procedure reported by [9], and detected at 520 nm by a UV-detector (Agilent Technologies, Inc., Santa Clara, CA, USA). Calibration curve was obtained with oenin-chloride (Extrasynthese, Genay, France). Total anthocyanins were expressed as malvidin 3-glucoside equivalents and included monoglucoside, acetyl-glucoside, and *p*-coumaroyl-glucoside fractions. The anthocyanin profile was calculated for the monoglucoside fraction as the percentage of 3'5'-OH derivatives.

### Transcript profiling

Total RNA was extracted as described in [61], treated with RNase-Free DNase I Set (Qiagen S.p.A., Milan, Italy), and purified with RNeasy MinElute Cleanup (Qiagen S.p.A., Milan, Italy) according to manufacturer's instructions. Complete removal of gDNA was assessed by direct use of treated RNA as a template for PCR reactions using the gene *VvUbiquitin1*. Absence of PCR products was visually inspected in 1% agarose gel stained with ethidium bromide. Absence of gDNA in reverse-transcribed samples was further confirmed by the melting curve performed during qPCR cycling using the intron-flanking primers for the normalisation gene *VvUbiquitin1*. The integrity of treated RNA was verified by electrophoresis in 1% agarose gel stained with ethidium bromide. RNA purity (A_260_/A_280 _nm) and quantification were estimated using a Nanodrop 1000 spectrophotometer (Thermo Fisher Scientific Inc., Wilmington, DE, USA). cDNA was synthesised using 2 μg of treated RNA, 0.5 μM (dT)_18 _primer, 0.5 mM dNTPs (Promega, Madison, WI, USA Cat# U1240), and 100 U of M-MLV Reverse Transcriptase (Promega, Madison, WI, USA Cat# N1701) in a 20 μL reaction volume supplemented with 20 U of RNasin Plus RNase inhibitor (Promega, Madison, WI, USA Cat# N2611) and incubated at 37°C for 90 min. Quantitative RT-PCR was carried out on a DNA Engine Opticon2 (MJ Research, Waltham, MA, USA) in a 20 μL reaction volume containing 5 μL of 20-fold diluted cDNA, 0.4 U of HotMaster *Taq *polymerase, 4.0 mM Magnesium acetate, 0.4 mM dNTPs, 1X SYBR solution (5 PRIME GmbH, Hamburg, Germany, Cat# 2200800), and 200 nM of each forward and reverse primer. Thermal cycling parameters were: initial denaturation at 95°C for 3 min, followed 40 cycles of 94°C for 15 s, 61°C for 20 s, and 68°C for 30 s, plate read at 78-82°C depending on each primer pair for 1 s, melting curve from 65°C to 95°C, read every 1°C, hold 1 s, and a final extension at 68°C for 5 min. Threshold cycle (C_t_) was determined using the Opticon Monitor analysis software (version 2.02, MJ Research, Waltham, MA, USA) with a threshold level of fluorescence signal detection of log -1.7. Aliquots from the same cDNA were run in duplicate in the qPCR assay. Intra-assay repeatability between technical replicates was below 1 C_t_. All assays included no-template controls. Relative gene expression of the target gene was calculated with the 2^-ΔΔCt ^method, using the constitutive expression of the housekeeping *Ubiquitin *gene (*VvUbiquitin1*) [6]. *VvUbiquitin1 *has been widely used in qPCR experiments conducted in grapevine across various organs by several research groups, in particular for berry samples. Semi-quantitative PCR was performed upon cDNA normalisation based on *VvUbiquitin1 *expression and visualised in a 1% agarose gel stained with ethidium bromide, or on SSCP gel stained with silver nitrate.

## Authors' contributions

LF planned and conducted most of the field and lab experiments; SDC carried out HPLC analyses and run statistics on transcriptional and metabolite data; GAG, MM, and RT contributed to the interpretation of results and participated in drafting the manuscript; GDG conceived the design of this study, analysed the structural organisation of the gene family and drafted the manuscript. All authors have read and approved the final manuscript.

## Supplementary Material

Additional file 1 **Chromosomal positions of *F3'H*s and *F3'5'H*s in the grapevine genome**. Click here for file

Additional file 2 **Lack of gene collinearity around the isolated *F3'5'Hp *on chr8 and the *F3'5'H *multi-copy array on chr6**. The positions of *F3'5'H*s are shown as cyan ticks, gene models are shown in blue, and partial peptides are shown in grey, above and below the corresponding GEvo diagrams. Regions of sequence similarity were identified by comparing both DNA strands using GEvo, and are shown as red ticks or boxes. Red lines connect regions of similarity within gene models, all other regions of similarity are either microsatellite DNA or transposable elements. Gaps in the sequence assembly are indicated by orange boxes. Click here for file

Additional file 3 **Genome landscape in a 10-kb window around *F3'5'H*s in the chr6 array**. Exons are indicated as thick blue bars, introns are thin blue connectors. Coloured models indicate annotated TEs. Sequence gaps (Ns) in the PN40024 genome assembly are indicated by dotted red lines. Click here for file

Additional file 4 **Genomic organisation and transcription of two copies of *F3'H*s present in the grapevine genome**. In **section a**, exon/intron structure of *F3'H*s is shown as blue boxes (exons) connected by blue lines (introns); TEs are shown as coloured boxes. In **section b **and **c**, selective amplification of exon junctions astride the terminal intron and expression of each *F3'H *copy are shown. Two primer pairs (orange and green triangles) were designed in the internal and terminal exons. The terminal intron varied in size between 249 bp and 96 bp in *F3'Ha *and *-b*, respectively. Each primer pair anneals perfectly to the target *F3'H*, but has a mismatch at the 3'-terminal nucleotide with the paralogous *F3'H*. Selectivity of primer pairs for either *F3'Ha *or *F3'Hb *was validated by amplifying PN40024 genomic DNA and by Sanger sequencing of the PCR amplicons. Selectivity for either *F3'Ha *or *F3'Hb *was also confirmed by assessing the size of the amplified genomic DNA (vs. the size prediction of 523 bp and 370 bp astride the second intron in *F3'Ha *and *F3'Hb*, respectively) and, for the expressed *F3'Ha*, by inferring intron size from the comparison between amplicons from genomic DNA and cDNA. Expression of *F3'Ha *was assessed by semi-quantitative PCR using cDNA from leaf, petiole, tendril, flower, shoot, and berry skin and flesh, in two grapevine cultivars ('Merlot' and 'Aglianico'). Expression of *F3'Ha *was also assessed in berry skin of four cultivars ('Aglianico', 'Marzemino', 'Grignolino', and 'Nebbiolo') at four stages of fruit development. cDNA was normalised using the constitutive gene *VvUbiquitin*. Transcripts of *F3'Hb *were never detected under the same experimental conditions. In **section d**, expression of *F3'Ha *was assessed by quantitative PCR in berry skin at 8 developmental stages in the cultivars 'Aglianico', 'Marzemino', 'Grignolino', and 'Nebbiolo'. Transcript levels of *F3'Ha *increased at full-veraison (stage of 100% coloured berries) by approximately 2-fold in all cultivars, with substantial differences among cultivars only at harvest. Transcript levels are expressed as arbitrary units, normalised using the constitutive gene coding for *VvUbiquitin*. Bars represent the standard deviation of three biological replicates. Letters above the histograms indicate significant differences between means, based on a Student-Newman-Keuls test (P < 0.05). Click here for file

Additional file 5 **Evolutionary relationships among grapevine *F3'5'H*s**. Phylogenetic trees are inferred using the Maximum Parsimony method and are based on (**a**) mRNA sequence alignments of all grapevine *F3'5'H*s and (**b**) intron sequences of *F3'5'H*s that reside in duplicate blocks on chr6. The most parsimonious tree was obtained using the Close-Neighbor-Interchange algorithm with search level 3, in which the initial trees were obtained with the random addition of sequences. The rectangular and radiation trees are drawn to scale, with branch lengths calculated using the average pathway method, and are expressed in units of the number of changes over the whole sequence. There were a total of 419 positions in the mRNA dataset, out of which 39 were parsimony informative, and 1546 positions in the intron dataset, out of which 180 were parsimony informative. For each gene, tree topology is compared to genomic location. Bootstrap values >70 are reported above the corresponding branch. DNA sequences were aligned with ClustalX and trees were obtained using MEGA4. (**c**) Tree based on LAGAN alignments of 5' regulatory sequences 2-kb upstream of the translation start codon. Click here for file

Additional file 6 **Multiple alignments of non-coding DNA within each of 9 tandemly duplicated blocks in the *F3'5'H *locus on chr6**. On top of each page, coloured bars indicate annotated TEs in the PN40024 genome; sequence gaps (Ns) in the genome assembly are indicated by dotted red lines. Plots of sequence identity range from 50 to 100% on the y-axis in the LAGAN multi-panels. The number of base pairs shared by each duplicated block with the reference block (on top) is given on the right-hand side, with the average nucleotide identity. Click here for file

Additional file 7 **Multiple alignments of non-coding DNA in 10-kb surrounding duplicate *F3'5'H *genes**. In the panel on top of each page, *F3'5'H *exons are indicated as thick blue bars, introns are thin blue connectors. Coloured boxes indicate annotated TEs. Plots of sequence identity range from 50 to 100% on the *y*-axis in the LAGAN multi-panels. Click here for file

Additional file 8 **Conservation and SSCP polymorphisms of duplicate *F3'5'H*s in the family Vitaceae**. PCR amplicons were obtained from genomic DNA using copy-specific primers. DNA samples included the ornamental grapevines Virginia creeper *Parthenocissus quinquefolia*, native to Northeastern-America, and the porcelain berry *Ampelopsis brevipedunculata*, native to temperate areas of Asia (segment A), wild grapevines (segment B) including the 2*n *= 40 *Muscadinia rotundifolia*, two North American species *V. riparia *and *V. candicans*, two Asian species *V. armata *and *V. romanetii*, and a spontaneous ecotype of *V. vinifera *ssp *sylvestris *collected in woods of Northeastern Italy; red-skinned cultivars of the domesticated *V. vinifera *ssp *sativa *(segment C); white-skinned cultivars (Pinot bud sports with mutations for skin colour are shown beside Pinot blanc) and the nearly-homozygous line PN40024 (segment D). PCR amplicons were run in agarose gel (**section a**) and in denaturing gel for detecting single-strand conformational polymorphisms (**section b**). Among *F3'5'H*s, the isolated gene copies *F3'5'Hp, -o, -m*, and *-n *showed the lowest levels of conformational polymorphisms, while segmentally duplicated *F3'5'H*s were more variable across taxa. Click here for file

Additional file 9 **Amino acid alignment of substrate recognition sites (SRS) and functional domains for hydroxylation activity (CR1) in plant *F3'5'H*s**. Amino acid positions crucial for 3' vs. 3'5'-hydroxylation in CR1 and SRS6 are indicated by black arrows; significant amino acid substitutions in grapevine *F3'5'H*s are in green background. Relevant amino acid substitutions within domains putatively involved in substrate recognition are highlighted in grapevine *F3'5'H*s by blue background when they are unique with respect to all other plant *F3'5'H*s or when they are shared exclusively with either monocot *F3'5'H*s or other plant *F3'Hs*, as possible remnants of ancestral transition stages in the evolution of dicot *F3'5'H*s. Click here for file

Additional file 10 **Transcripts of duplicate *F3'5'H*s detected in various organs of two grape cultivars by semiquantitative PCR**. Bold + indicates high expression of PCR amplicons visualised on agarose gel stained with ethidium bromide (see Figure 7), regular + indicates weak expression detected only by the more sensitive silver staining, - indicates lack of detectable transcripts. Click here for file

Additional file 11 **Expression of duplicate *F3'5'H*s in berry skin of four cultivars accumulating 3'5'-OH anthocyanins detected by semiquantitative PCR**. Berry skin was sampled at four developmental stages. cDNA was normalised using the housekeeping *Ubiquitin *gene. *UFGT *was used as a marker for anthocyanin gene expression. Even though the pre-veraison berries were sampled over green bunches immediately before visible colour transition, expression of *UFGT *had already been triggered in 'Aglianico' and was barely detectable in 'Nebbiolo'. Either primer of the oligonucleotide pairs targeting the *F3'5'Hi *and *-l *copies anneals to either exon of the corresponding gene model. The corresponding PCR bands obtained from gDNA are approximately 400 bp longer than the cDNA amplicons shown in the stripes of the electrophoresis gel of this figure. Click here for file

Additional file 12 **Analysis of variance of duplicate *F3'5'H *expression in berry skin of four cultivars along eight developmental stages**. Click here for file

Additional file 13 **Primer sequences for grapevine *F3'H*s and *F3'5'H*s**. Click here for file

Additional file 14 **Berry sampling in four red-skinned cultivars and a green-skinned cultivar (Tocai) across eight developmental stages**. Click here for file
