# Supplementary material for: Expansion and subfunctionalisation of flavonoid 3',5'-hydroxylases in the grapevine lineage
Source: BMC Genomics. 2010 Oct 12;11:562. doi: 10.1186/1471-2164-11-562 (PMC3091711; doi:10.1186/1471-2164-11-562)
Supplement: Additional file 1 — Chromosomal positions of F3'Hs and F3'5'Hs in the grapevine genome. [file 1471-2164-11-562-S1.PDF]

Additional file 1 - Chromosomal positions of *F3´H*s and *F3´5´H*s in the grapevine genome

sequence feature	name	chromosomal positions		notes/reference
SSR marker	VrZag30	chr6:15557815..15557839		Castellarin et al. 2006
SSCP marker	BES9C17-FM	chr6:15607288..15607645		Castellarin et al. 2006
F3´5´H	*F3´5´Ha*	chr6:15665962..15666150	first exon	first exon truncated at 5'end
F3´5´H	*F3´5´Ha*	chr6:15666566..15666865	terminal exon	terminal exon truncated at 3'end
F3´5´H	*F3´5´Hb*	chr6:15670204..15670863	first exon	first exon truncated at 5'end
F3´5´H	*F3´5´Hb*	chr6:15671267..15671893	terminal exon	
F3´5´H	*F3´5´Hc*	chr6:15714628..15715524	first exon	
F3´5´H	*F3´5´Hc*	chr6:15715940..15716227	terminal exon	terminal exon truncated at 3'end
F3´5´H	*F3´5´Hd*	chr6:15718279..15719175	first exon	full-length
F3´5´H	*F3´5´Hd*	chr6:15719579..15720205	terminal exon	full-length
F3´5´H	*F3´5´He*	chr6:15763822..15764193	first exon	first exon truncated at both ends
F3´5´H	*F3´5´Hf*	chr6:15798856..15799752	first exon	full-length
F3´5´H	*F3´5´Hf*	chr6:15800158..15800784	terminal exon	full-length
F3´5´H	*F3´5´Hg*	chr6:15834998..15835636	first exon	first exon truncated at 5'end
F3´5´H	*F3´5´Hg*	chr6:15835958..15836584	terminal exon	
SSCP marker	BES17K4-FM	chr6:15851252..15851578		Castellarin et al. 2006
F3´5´H	*F3´5´Hh*	chr6:15904021..15904182	first exon	first exon invaded by Gyspy
F3´5´H	*F3´5´Hh*	chr6:15914821..15915111	first exon	
F3´5´H	*F3´5´Hh*	chr6:15915525..15916155	terminal exon	
F3´5´H	*F3´5´Hi*	chr6:15950229..15950481	first exon	first exon invaded by Copia
F3´5´H	*F3´5´Hi*	chr6:15956483..15957127	first exon	
F3´5´H	*F3´5´Hi*	chr6:15957532..15958158	terminal exon	
F3´5´H	*F3´5´Hj*	chr6:16008924..16009820	first exon	full-length
F3´5´H	*F3´5´Hj*	chr6:16010235..16010861	terminal exon	full-length
F3´5´H	*F3´5´Hk*	chr6:16048254..16049149	first exon	1-bp frameshift deletion in the first exon, following aa 142
F3´5´H	*F3´5´Hk*	chr6:16049540..16050166	terminal exon	
F3´5´H	*F3´5´Hl*	chr6:16057809..16058705	first exon	full-length
F3´5´H	*F3´5´Hl*	chr6:16059095..16059721	terminal exon	full-length
F3´5´H	*F3´5´Hm*	chr6:16106732..16107628	first exon	
F3´5´H	*F3´5´Hm*	chr6:16108358..16109243	first exon	unusual structure: first exon and intron repeated
F3´5´H	*F3´5´Hm*	chr6:16110014..16110640	terminal exon	
F3´5´H	*F3´5´Hn*	chr6:16228524..16229420	first exon	full-length
F3´5´H	*F3´5´Hn*	chr6:16230220..16230846	terminal exon	full-length
F3´5´H	*F3´5´Ho*	chr6:16319312..16319938	terminal exon (-)	full-length
F3´5´H	*F3´5´Ho*	chr6:16320481..16321380	first exon (-)	full-length
SSR marker	VMC3F12	chr6:16361956..16362081		Castellarin et al. 2006
SSCP marker	BES38C24-RM	chr6:16408676..16408695		Castellarin et al. 2006
F3´5´H	*F3´5´Hp*	chr8:19086144..19087049		full-length
F3´5´H	*F3´5´Hp*	chr8:19087149..19087784		full-length
SSR marker	VMC3A9	chr17:4984101..4984243		Castellarin et al. 2006
SSR marker	VVIN73	chr17:5504409..5504674		Castellarin et al. 2006
SSCP marker	BES5A23-FM	chr17:7996989..7997206		Castellarin et al. 2006
F3´H	*F3´Ha*	chr17:8011925..8012359	first exon	full-length
F3´H	*F3´Ha*	chr17:8012795..8013247	internal exon	full-length
F3´H	*F3´Ha*	chr17:8013496..8014137	terminal exon	full-length
F3´H	*F3´Hb*	chr17:8035387..8035821	first exon	full-length
F3´H	*F3´Hb*	chr17:8036302..8036754	internal exon	full-length
F3´H	*F3´Hb*	chr17:8036850..8037488	terminal exon	full-length
SSR marker	VMC9G4	chr17:8681970..8681991		Castellarin et al. 2006
SSR marker	VVIN68	chr17:8919226..8919205		Castellarin et al. 2006
