# Supplementary material for: Expansion and subfunctionalisation of flavonoid 3',5'-hydroxylases in the grapevine lineage
Source: BMC Genomics. 2010 Oct 12;11:562. doi: 10.1186/1471-2164-11-562 (PMC3091711; doi:10.1186/1471-2164-11-562)

Additional file 2 - Lack of gene collinearity around the isolated *F3´5´Hp* on chr8 and the *F3´5´H* multi-copy array on chr6

The positions of *F3´5´H*s are shown as cyan ticks, gene models are shown in blue, and partial peptides are shown in grey, above and below the corresponding GEvo diagrams. Regions of sequence similarity were identified by comparing both DNA strands using GEvo, and are shown as red ticks or boxes. Red lines connect regions of similarity within gene models, all other regions of similarity are either microsatellite DNA or transposable elements. Gaps in the sequence assembly are indicated by orange boxes.

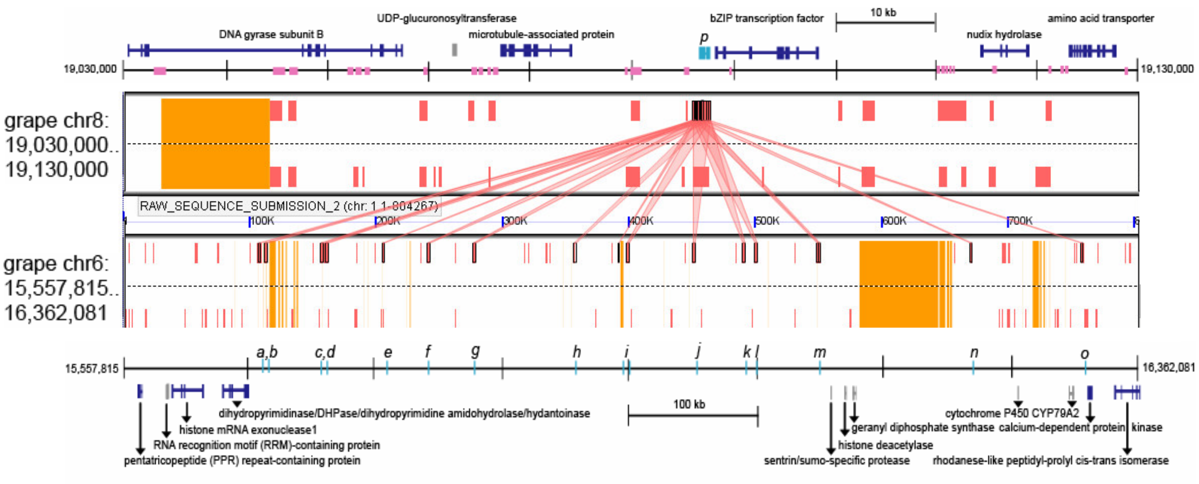

Supplement: Additional file 2 — Lack of gene collinearity around the isolated F3'5'Hp on chr8 and the F3'5'H multi-copy array on chr6. The positions of F3'5'Hs are shown as cyan ticks, gene models are shown in blue, and partial peptides are shown in grey, above and below the corresponding GEvo diagrams. Regions of sequence similarity were identified by comparing both DNA strands using GEvo, and are shown as red ticks or boxes. Red lines connect regions of similarity within gene models, all other regions of similarity are either microsatellite DNA or transposable elements. Gaps in the sequence assembly are indicated by orange boxes. [file 1471-2164-11-562-S2.PDF]
