# Supplementary material for: Expansion and subfunctionalisation of flavonoid 3',5'-hydroxylases in the grapevine lineage
Source: BMC Genomics. 2010 Oct 12;11:562. doi: 10.1186/1471-2164-11-562 (PMC3091711; doi:10.1186/1471-2164-11-562)

Additional file 3 - Genome landscape in a 10kb window around *F3´5´Hs* in the chr6 array

Exons are indicated as thick blue bars, introns are thin blue connectors. Coloured models indicate annotated TEs. Sequence gaps (Ns) in the PN40024 genome assembly are indicated by dotted red lines.

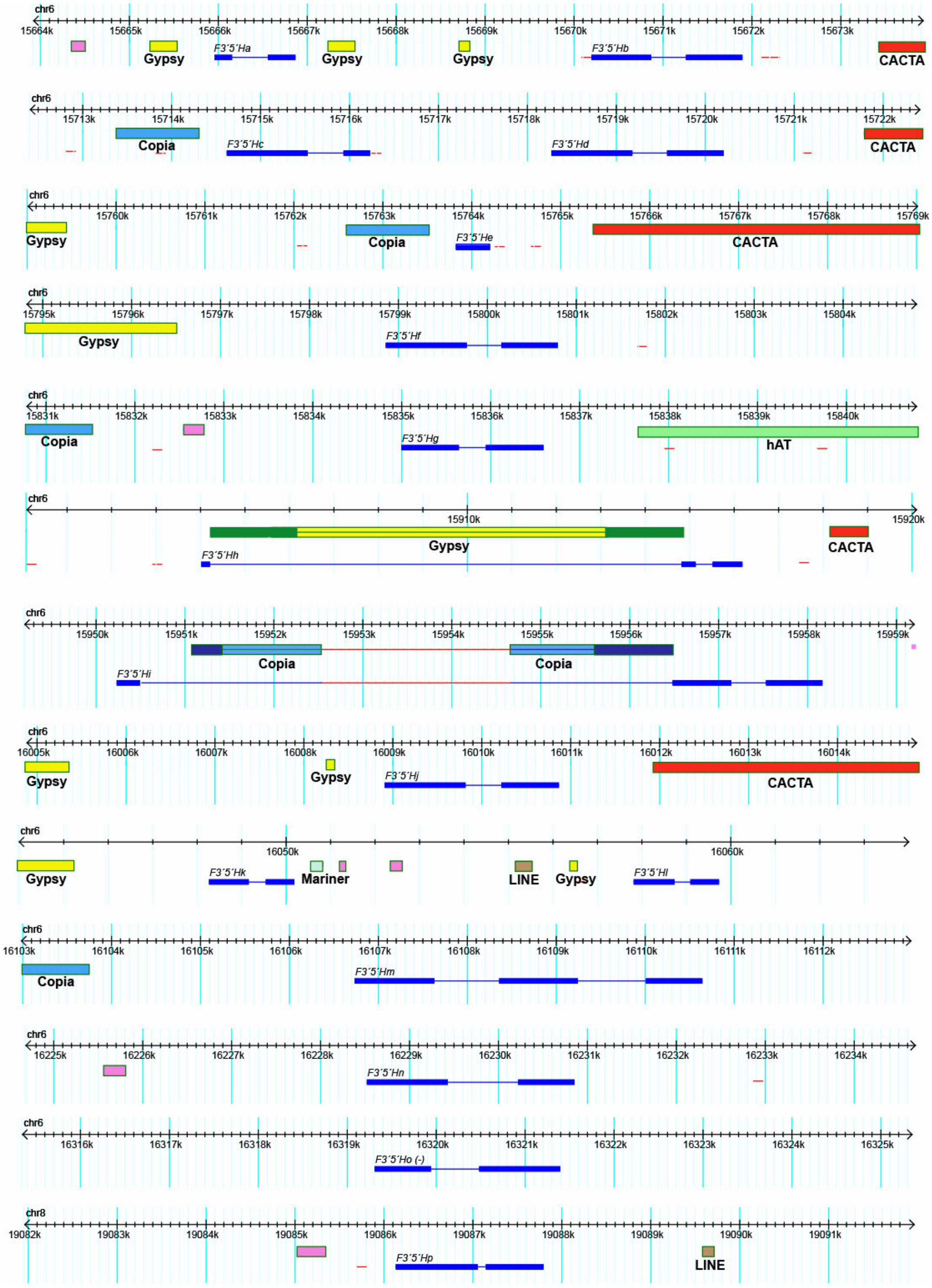

Supplement: Additional file 3 — Genome landscape in a 10-kb window around F3'5'Hs in the chr6 array. Exons are indicated as thick blue bars, introns are thin blue connectors. Coloured models indicate annotated TEs. Sequence gaps (Ns) in the PN40024 genome assembly are indicated by dotted red lines. [file 1471-2164-11-562-S3.PDF]
