# Supplementary material for: Expansion and subfunctionalisation of flavonoid 3',5'-hydroxylases in the grapevine lineage
Source: BMC Genomics. 2010 Oct 12;11:562. doi: 10.1186/1471-2164-11-562 (PMC3091711; doi:10.1186/1471-2164-11-562)
Supplement: Additional file 4 — Genomic organisation and transcription of two copies of F3'Hs present in the grapevine genome. In section a, exon/intron structure of F3'Hs is shown as blue boxes (exons) connected by blue lines (introns); TEs are shown as coloured boxes. In section b and c, selective amplification of exon junctions astride the terminal intron and expression of each F3'H copy are shown. Two primer pairs (orange and green triangles) were designed in the internal and terminal exons. The terminal intron varied in size between 249 bp and 96 bp in F3'Ha and -b, respectively. Each primer pair anneals perfectly to the target F3'H, but has a mismatch at the 3'-terminal nucleotide with the paralogous F3'H. Selectivity of primer pairs for either F3'Ha or F3'Hb was validated by amplifying PN40024 genomic DNA and by Sanger sequencing of the PCR amplicons. Selectivity for either F3'Ha or F3'Hb was also confirmed by assessing the size of the amplified genomic DNA (vs. the size prediction of 523 bp and 370 bp astride the second intron in F3'Ha and F3'Hb, respectively) and, for the expressed F3'Ha, by inferring intron size from the comparison between amplicons from genomic DNA and cDNA. Expression of F3'Ha was assessed by semi-quantitative PCR using cDNA from leaf, petiole, tendril, flower, shoot, and berry skin and flesh, in two grapevine cultivars ('Merlot' and 'Aglianico'). Expression of F3'Ha was also assessed in berry skin of four cultivars ('Aglianico', 'Marzemino', 'Grignolino', and 'Nebbiolo') at four stages of fruit development. cDNA was normalised using the constitutive gene VvUbiquitin. Transcripts of F3'Hb were never detected under the same experimental conditions. In section d, expression of F3'Ha was assessed by quantitative PCR in berry skin at 8 developmental stages in the cultivars 'Aglianico', 'Marzemino', 'Grignolino', and 'Nebbiolo'. Transcript levels of F3'Ha increased at full-veraison (stage of 100% coloured berries) by approximately 2-fold in all cultivars, with substantial d [file 1471-2164-11-562-S4.PDF]

Additional file 4 - Genomic organisation and transcription of two copies of *F3´H*s present in the grapevine genome

In **section a**, exon/intron structure of *F3´H*s ia shown as blue boxes (exons) connected by blue lines (introns); TEs are shown as coloured boxes.

In **section b** and **c**, selective amplification of exon junctions astride the terminal intron and expression of each *F3´H* copy. Two primer pairs (orange and green triangles) were designed in the internal and terminal exons. The terminal intron varied in size between 249 bp and 96 bp in *F3´Ha* and *F3´Hb*, respectively. Each primer pair anneals perfectly to the target *F3´H*, but has a mismatch at the 3´ terminal nucleotide with the paralogous *F3´H*. Selectivity of primer pairs for either *F3´Ha* or *F3´Hb* was validated by amplifying PN40024 genomic DNA and by Sanger sequencing of the PCR amplicons. Selectivity for either *F3´Ha* or *F3´Hb* was also confirmed by assessing the size of the amplified genomic DNA (vs. the size prediction of 523 bp and 370 bp astride the second intron in *F3´Ha* and *F3´Hb*, respectively) and, for the expressed *F3´Ha*, by inferring intron size from the comparison between amplicons from genomic DNA and cDNA. Expression of *F3´Ha* was assessed by semi-quantitative PCR using cDNA from leaf, petiole, tendril, flower, shoot, and berry skin and flesh, in two grapevine cultivars (‘Merlot’ and ‘Aglianico’). Expression of *F3´Ha* was also assessed in berry skin of four cultivars (‘Aglianico’, ‘Marzemino’, ‘Grignolino’, and ‘Nebbiolo’) at four stages of fruit development. cDNA was normalised using the constitutive gene *VvUbiquitin*. Transcripts of *F3´Hb* were never detected under the same experimental conditions.

In **section d**, expression of *F3´Ha* was assessed by quantitative PCR in berry skin at 8 developmental stages in the cultivars ‘Aglianico’, ‘Marzemino’, ‘Grignolino’, and ‘Nebbiolo’. Transcript levels of *F3´Ha* increased at full-veraison (stage of 100% coloured berries) by approximately 2-fold in all cultivars, with substantial differences among cultivars only at harvest. Transcript levels are expressed as arbitrary units, normalised using the constitutive gene coding for *VvUbiquitin*. Bars represent standard deviation of three biological replicates. Differences among cultivars at each sampling date were tested for significance by one-way ANOVA. Means were separated by a Student–Newman–Keuls test and significant differences at $P<0.05$ are indicated by different letters.

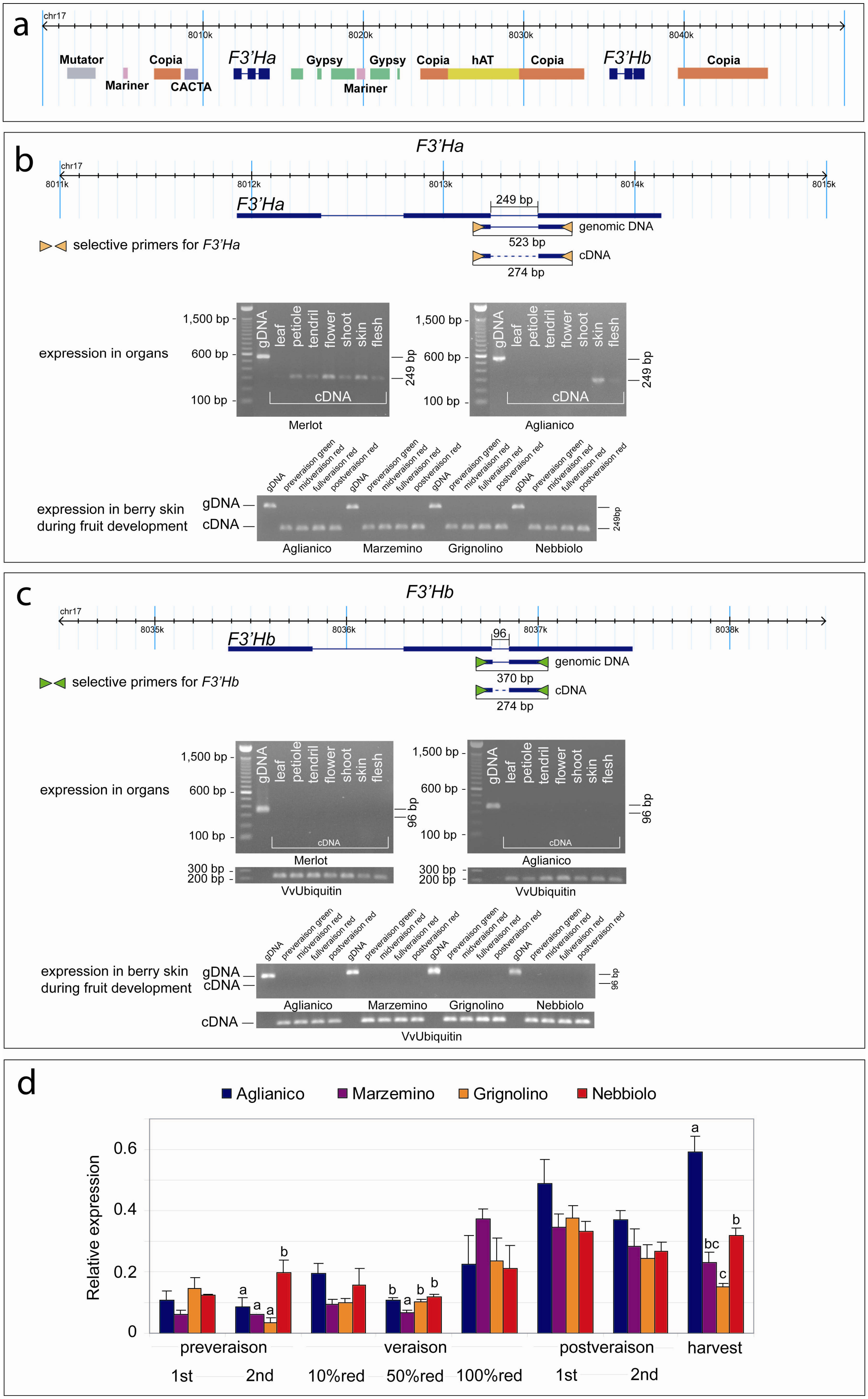
a
chr17
8010k
8020k
8030k
8040k
Mutator
Mariner
Copia
CACTA
F3'Ha
Gypsy
Mariner
Gypsy
Copia
hAT
Copia
F3'Hb
Copia
b
F3'Ha
chr17
8011k
8012k
8013k
8014k
8015k
F3'Ha
249 bp
genomic DNA
523 bp
cDNA
274 bp
selective primers for F3'Ha
expression in organs
1,500 bp
600 bp
100 bp
gDNA leaf petiole tendril flower shoot skin flesh
cDNA
249 bp
Merlot
Aglianico
expression in berry skin during fruit development
gDNA
cDNA
gDNA preveraison green midveraison red fullveraison red postveraison red
249bp
Aglianico
Marzemino
Grignolino
Nebbiolo
c
F3'Hb
chr17
8035k
8036k
8037k
8038k
F3'Hb
96
genomic DNA
370 bp
cDNA
274 bp
selective primers for F3'Hb
expression in organs
1,500 bp
600 bp
100 bp
96 bp
Merlot
Aglianico
300 bp
200 bp
VvUbiquitin
expression in berry skin during fruit development
Aglianico
Marzemino
Grignolino
Nebbiolo
VvUbiquitin
d
Aglianico
Marzemino
Grignolino
Nebbiolo
Relative expression
0.6
0.4
0.2
0
a
b
bc
c
preveraison
veraison
postveraison
harvest
1st
2nd
10%red
50%red
100%red
1st
2nd
