# Supplementary material for: Expansion and subfunctionalisation of flavonoid 3',5'-hydroxylases in the grapevine lineage
Source: BMC Genomics. 2010 Oct 12;11:562. doi: 10.1186/1471-2164-11-562 (PMC3091711; doi:10.1186/1471-2164-11-562)

Additional file 5 – Evolutionary relationships among grapevine *F3´5´H*s

Phylogenetic trees are inferred using the Maximum Parsimony method and are based on (**a**) mRNA sequence alignments of all grapevine *F3´5´H*s and (**b**) intron sequences of *F3´5´H*s that reside in duplicate blocks on chr6. The most parsimonious tree was obtained using the Close-Neighbor-Interchange algorithm with search level 3, in which the initial trees were obtained with the random addition of sequences. The rectangular and radiation trees are drawn to scale, with branch lengths calculated using the average pathway method and are expressed in the units of the number of changes over the whole sequence. There were a total of 419 positions in the mRNA dataset, out of which 39 were parsimony informative, and 1546 positions in the intron dataset, out of which 180 were parsimony informative. For each gene, tree topology is compared to genomic location. Bootstrap values >70 are reported above the corresponding branch. DNA sequences were aligned with ClustalX and trees were obtained using MEGA4. (**c**) Tree based on LAGAN alignments of 5´ regulatory sequences 2-kb upstream of the translation start codon.

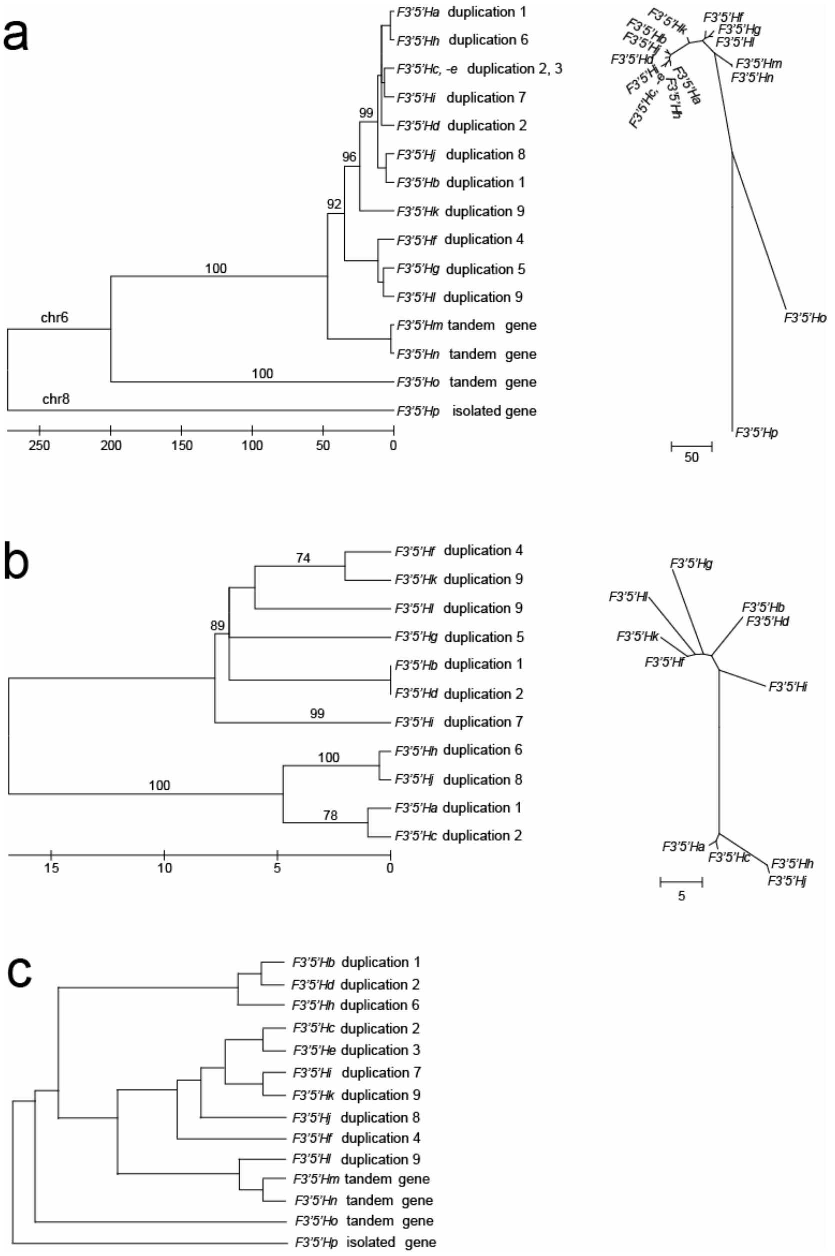

Supplement: Additional file 5 — Evolutionary relationships among grapevine F3'5'Hs. Phylogenetic trees are inferred using the Maximum Parsimony method and are based on (a) mRNA sequence alignments of all grapevine F3'5'Hs and (b) intron sequences of F3'5'Hs that reside in duplicate blocks on chr6. The most parsimonious tree was obtained using the Close-Neighbor-Interchange algorithm with search level 3, in which the initial trees were obtained with the random addition of sequences. The rectangular and radiation trees are drawn to scale, with branch lengths calculated using the average pathway method, and are expressed in units of the number of changes over the whole sequence. There were a total of 419 positions in the mRNA dataset, out of which 39 were parsimony informative, and 1546 positions in the intron dataset, out of which 180 were parsimony informative. For each gene, tree topology is compared to genomic location. Bootstrap values >70 are reported above the corresponding branch. DNA sequences were aligned with ClustalX and trees were obtained using MEGA4. (c) Tree based on LAGAN alignments of 5' regulatory sequences 2-kb upstream of the translation start codon. [file 1471-2164-11-562-S5.PDF]
