# Supplementary material for: Expansion and subfunctionalisation of flavonoid 3',5'-hydroxylases in the grapevine lineage
Source: BMC Genomics. 2010 Oct 12;11:562. doi: 10.1186/1471-2164-11-562 (PMC3091711; doi:10.1186/1471-2164-11-562)
Supplement: Additional file 6 — Multiple alignments of non-coding DNA within each of 9 tandemly duplicated blocks in the F3'5'H locus on chr6. On top of each page, coloured bars indicate annotated TEs in the PN40024 genome; sequence gaps (Ns) in the genome assembly are indicated by dotted red lines. Plots of sequence identity range from 50 to 100% on the y-axis in the LAGAN multi-panels. The number of base pairs shared by each duplicated block with the reference block (on top) is given on the right-hand side, with the average nucleotide identity. [file 1471-2164-11-562-S6.PDF]

Additional file 6 – Multiple alignments of non-coding DNA within each of 9 tandemly duplicated blocks in the *F3´5´H* locus on chr6

On top of each page, coloured bars indicate annotated TEs in the PN40024 genome; sequence gaps (Ns) in the genome assembly are indicated by dotted red lines. Plots of sequence identity range from 50 to 100% on the *y*-axis in the LAGAN multi-panels. The number of base pairs shared by each duplicated block with the reference block (on top) is given on the right-hand side, with the average nucleotide identity.

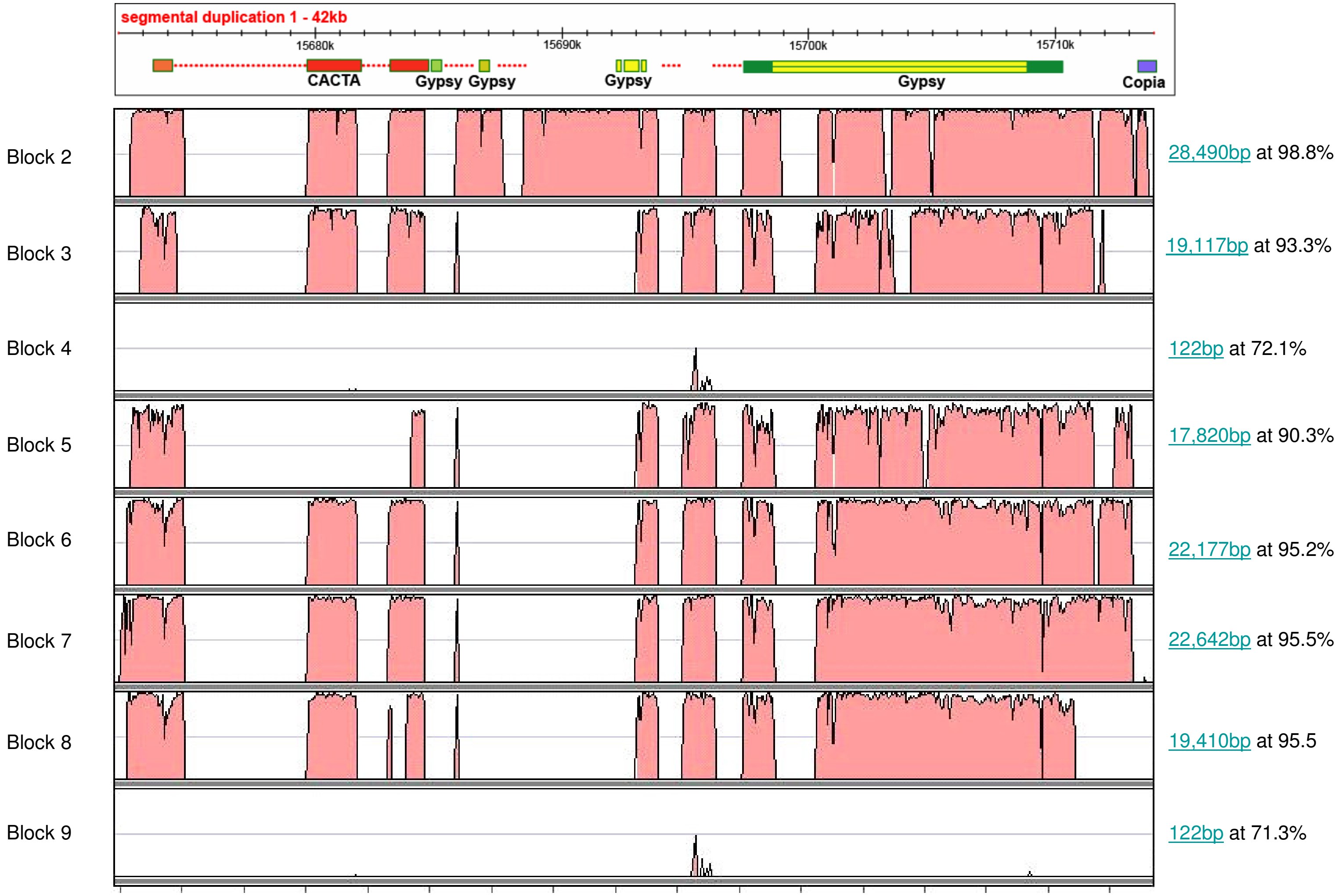

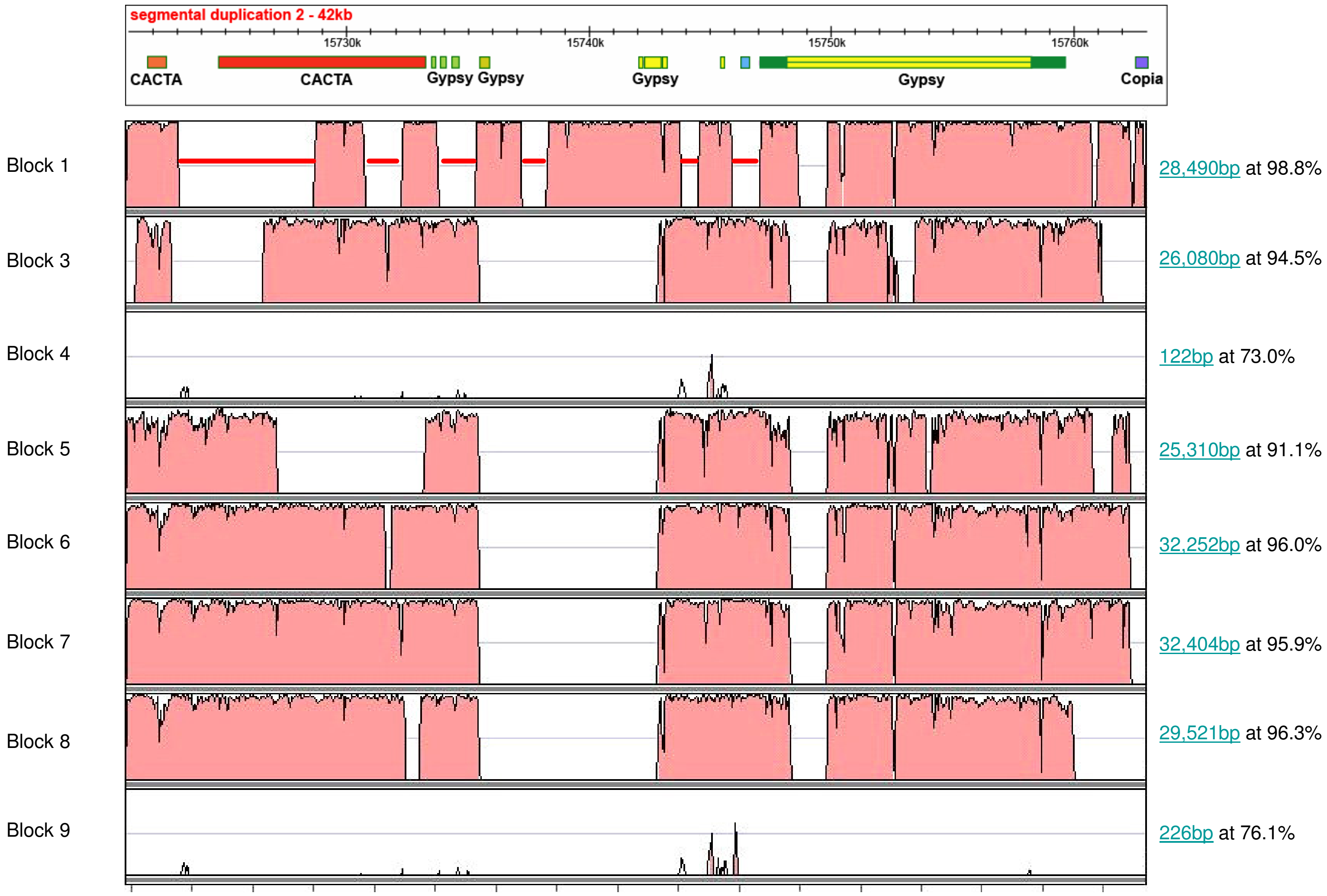

segmental duplication 2 - 42kb
15730k
15740k
15750k
15760k
CACTA
CACTA
Gypsy Gypsy
Gypsy
Gypsy
Copia
Block 1
28,490bp at 98.8%
Block 3
26,080bp at 94.5%
Block 4
122bp at 73.0%
Block 5
25,310bp at 91.1%
Block 6
32,252bp at 96.0%
Block 7
32,404bp at 95.9%
Block 8
29,521bp at 96.3%
Block 9
226bp at 76.1%

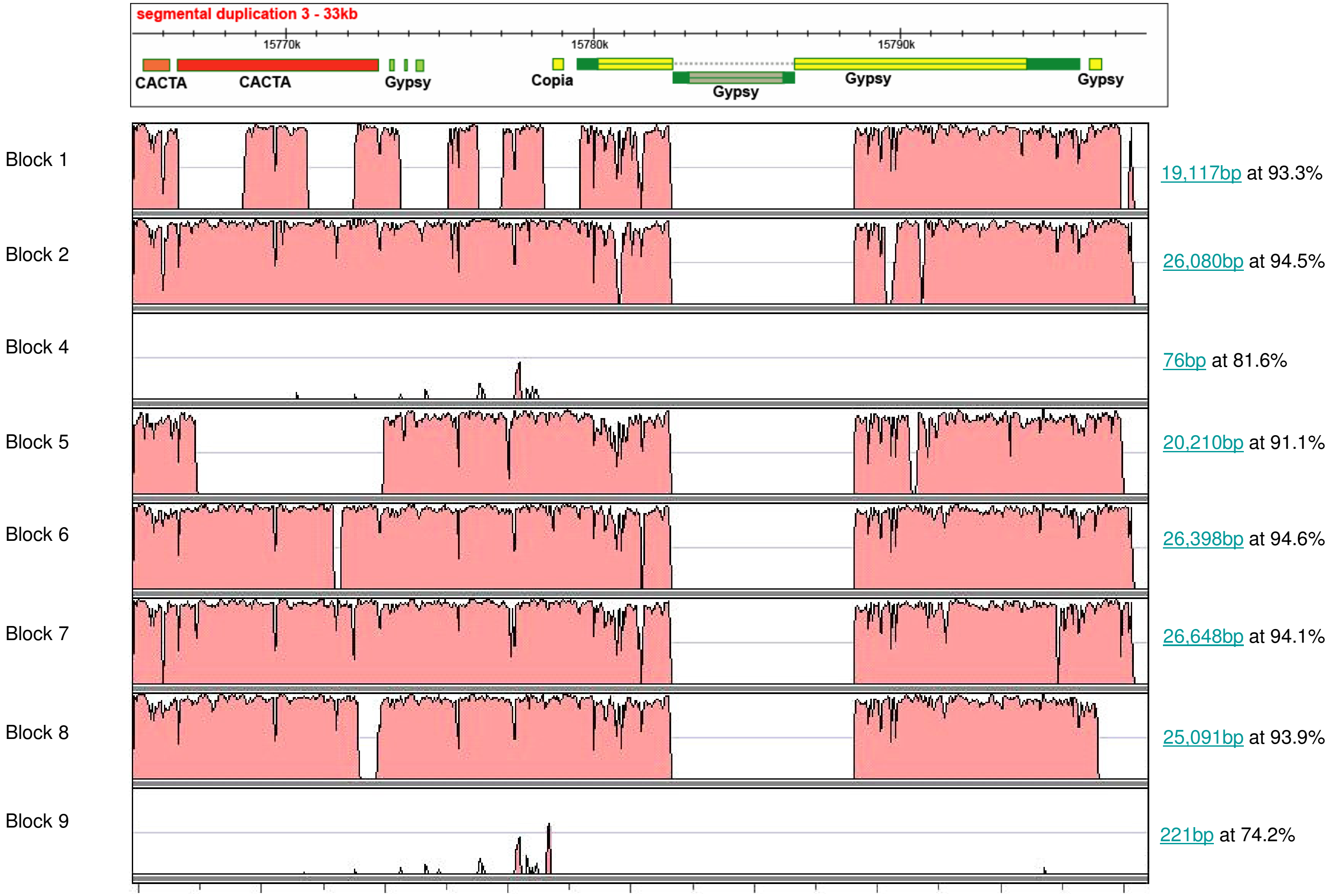
segmental duplication 3 - 33kb
15770k
15780k
15790k
CACTA
CACTA
Gypsy
Copia
Gypsy
Gypsy
Gypsy
Block 1
Block 2
Block 4
Block 5
Block 6
Block 7
Block 8
Block 9
19,117bp at 93.3%
26,080bp at 94.5%
76bp at 81.6%
20,210bp at 91.1%
26,398bp at 94.6%
26,648bp at 94.1%
25,091bp at 93.9%
221bp at 74.2%

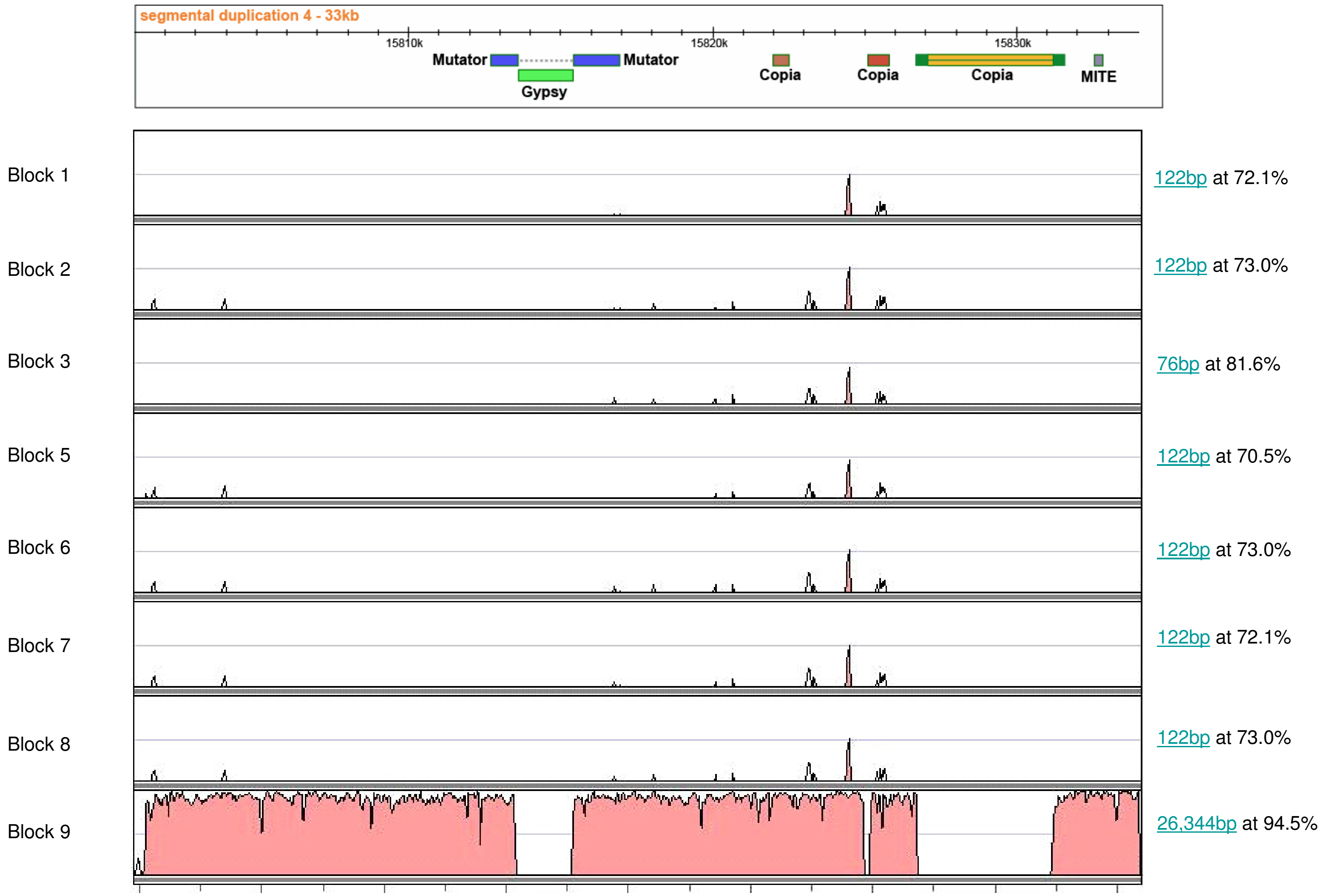
segmental duplication 4 - 33kb
15810k
15820k
15830k
Mutator
Mutator
Gypsy
Copia
Copia
Copia
MITE
Block 1
Block 2
Block 3
Block 5
Block 6
Block 7
Block 8
Block 9
122bp at 72.1%
122bp at 73.0%
76bp at 81.6%
122bp at 70.5%
122bp at 73.0%
122bp at 72.1%
122bp at 73.0%
26,344bp at 94.5%

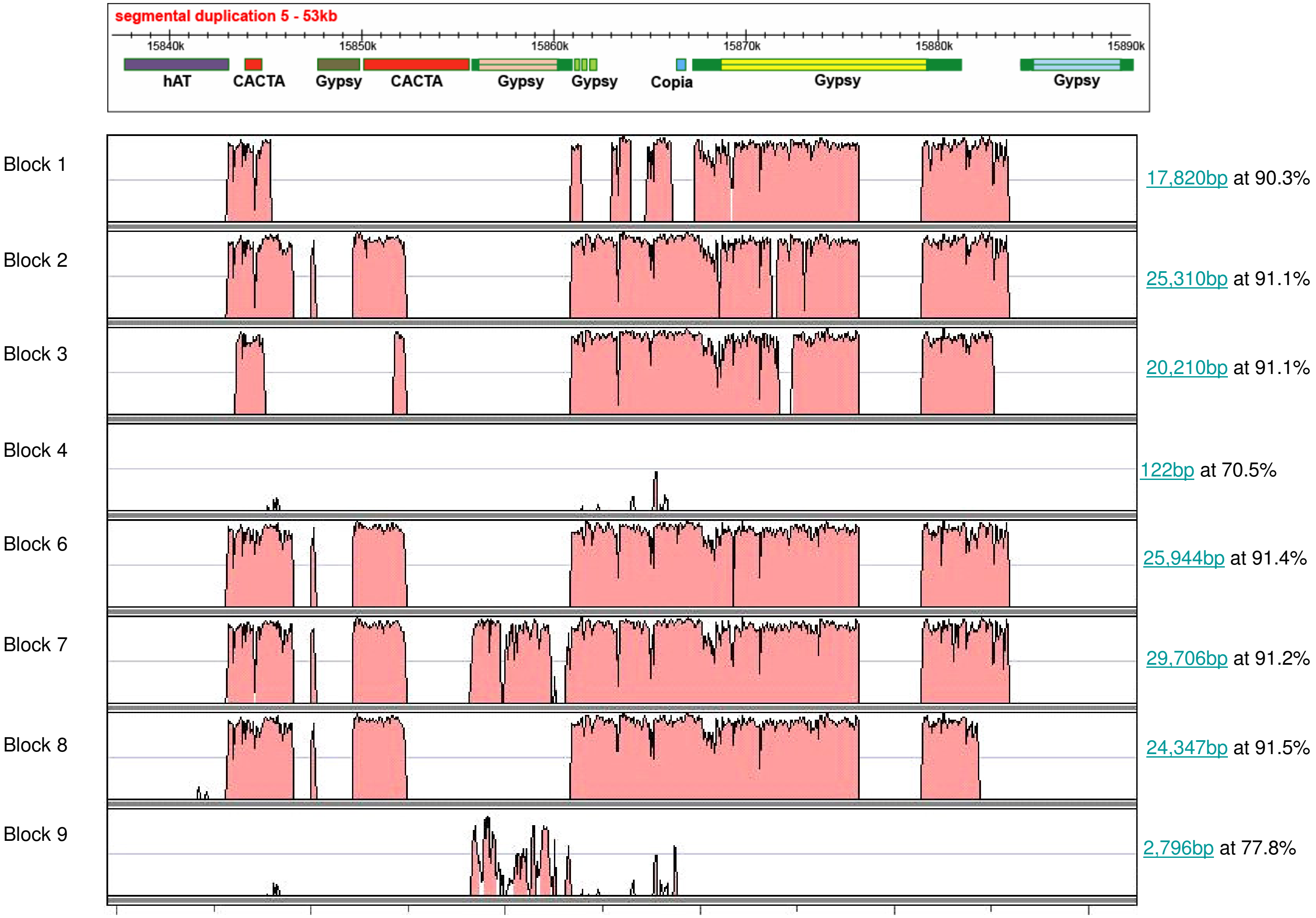
segmental duplication 5 - 53kb
15840k
15850k
15860k
15870k
15880k
15890k
hAT
CACTA
Gypsy
CACTA
Gypsy
Gypsy
Copia
Gypsy
Gypsy
Block 1
17,820bp at 90.3%
Block 2
25,310bp at 91.1%
Block 3
20,210bp at 91.1%
Block 4
122bp at 70.5%
Block 6
25,944bp at 91.4%
Block 7
29,706bp at 91.2%
Block 8
24,347bp at 91.5%
Block 9
2,796bp at 77.8%

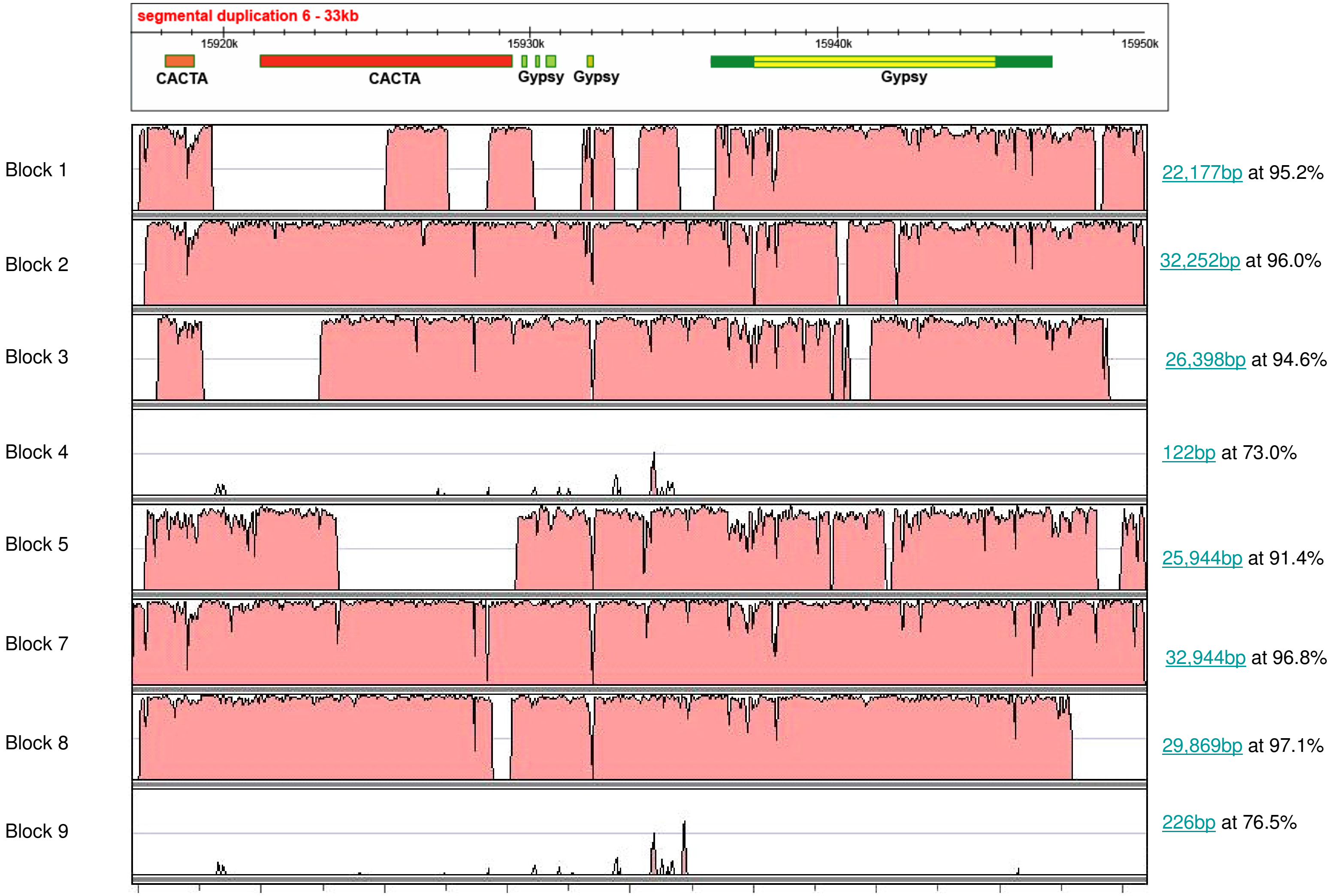

segmental duplication 6 - 33kb
15920k
15930k
15940k
15950k
CACTA
CACTA
Gypsy
Gypsy
Gypsy
Block 1
22,177bp at 95.2%
Block 2
32,252bp at 96.0%
Block 3
26,398bp at 94.6%
Block 4
122bp at 73.0%
Block 5
25,944bp at 91.4%
Block 7
32,944bp at 96.8%
Block 8
29,869bp at 97.1%
Block 9
226bp at 76.5%

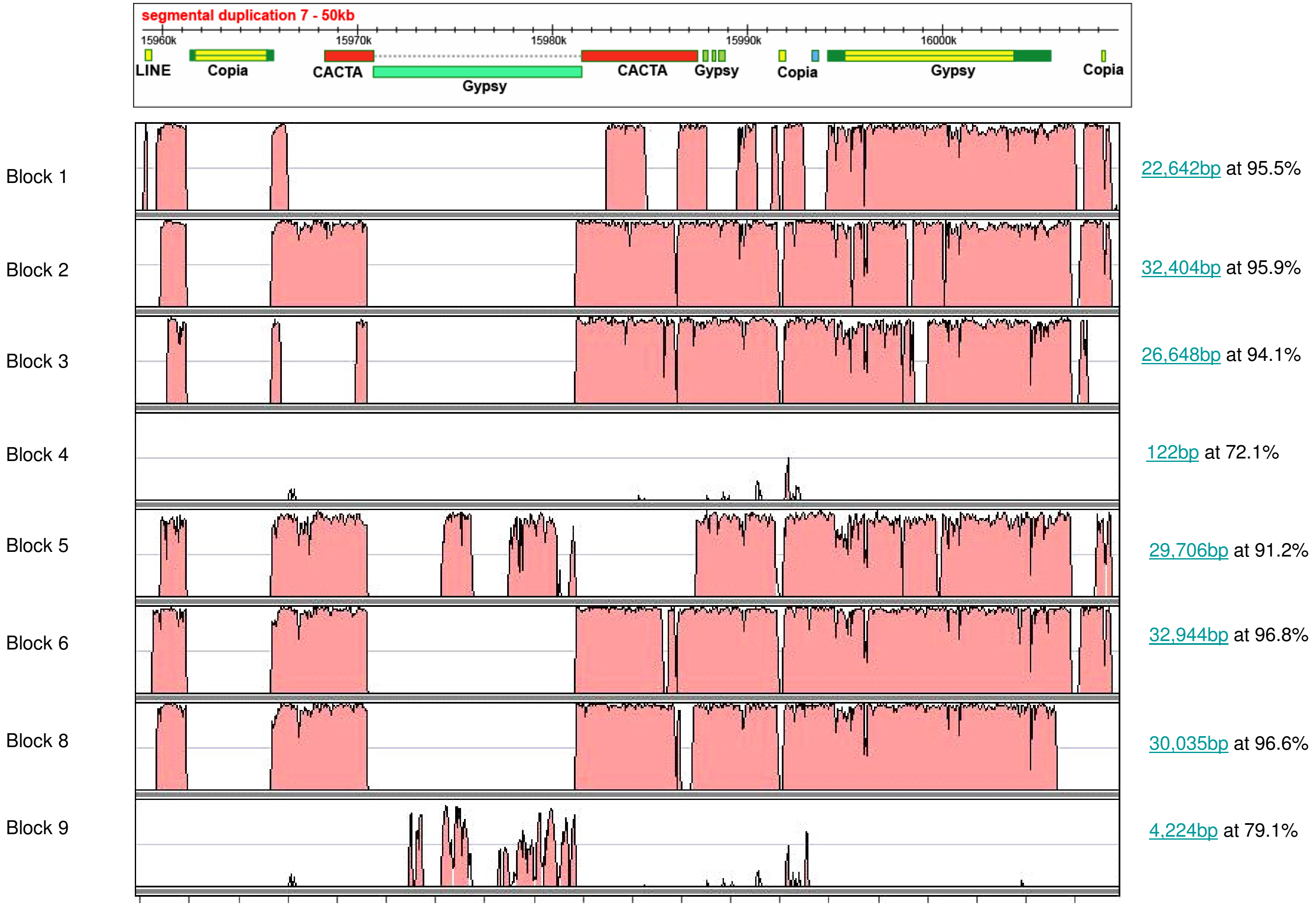
segmental duplication 7 - 50kb
15960k
15970k
15980k
15990k
16000k
LINE
Copia
CACTA
Gypsy
CACTA
Gypsy
Copia
Gypsy
Copia
Block 1
22,642bp at 95.5%
Block 2
32,404bp at 95.9%
Block 3
26,648bp at 94.1%
Block 4
122bp at 72.1%
Block 5
29,706bp at 91.2%
Block 6
32,944bp at 96.8%
Block 8
30,035bp at 96.6%
Block 9
4,224bp at 79.1%

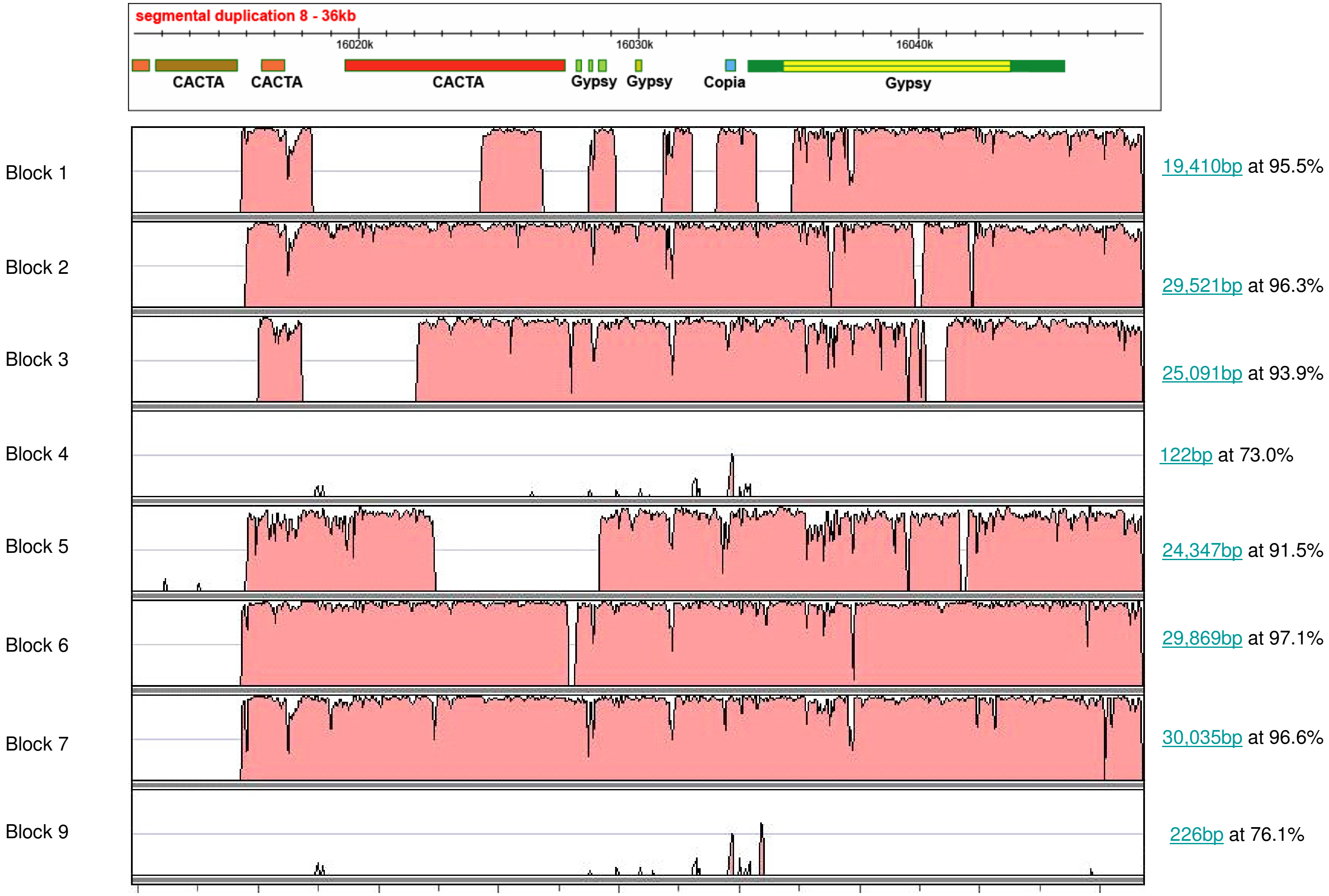
segmental duplication 8 - 36kb
16020k
16030k
16040k
CACTA
CACTA
CACTA
Gypsy
Gypsy
Copia
Gypsy
Block 1
19,410bp at 95.5%
Block 2
29,521bp at 96.3%
Block 3
25,091bp at 93.9%
Block 4
122bp at 73.0%
Block 5
24,347bp at 91.5%
Block 6
29,869bp at 97.1%
Block 7
30,035bp at 96.6%
Block 9
226bp at 76.1%

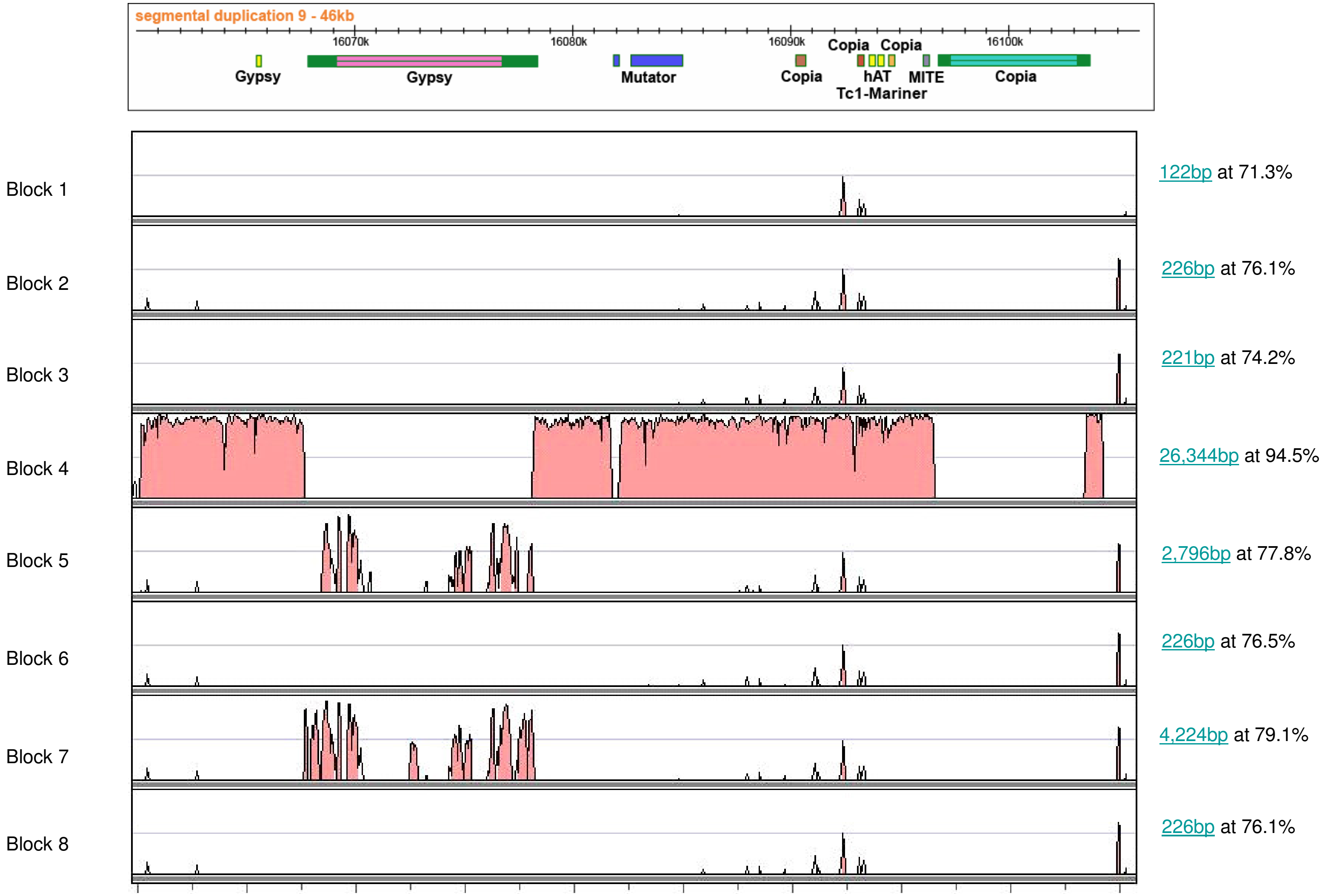

segmental duplication 9 - 46kb
16070k
16080k
16090k
16100k
Gypsy
Gypsy
Mutator
Copia
Copia
Copia
Copia
hAT
MITE
Tc1-Mariner
Block 1
Block 2
Block 3
Block 4
Block 5
Block 6
Block 7
Block 8
122bp at 71.3%
226bp at 76.1%
221bp at 74.2%
26,344bp at 94.5%
2,796bp at 77.8%
226bp at 76.5%
4,224bp at 79.1%
226bp at 76.1%
