# Supplementary material for: Expansion and subfunctionalisation of flavonoid 3',5'-hydroxylases in the grapevine lineage
Source: BMC Genomics. 2010 Oct 12;11:562. doi: 10.1186/1471-2164-11-562 (PMC3091711; doi:10.1186/1471-2164-11-562)
Supplement: Additional file 7 — Multiple alignments of non-coding DNA in 10-kb surrounding duplicate F3'5'H genes. In the panel on top of each page, F3'5'H exons are indicated as thick blue bars, introns are thin blue connectors. Coloured boxes indicate annotated TEs. Plots of sequence identity range from 50 to 100% on the y-axis in the LAGAN multi-panels. [file 1471-2164-11-562-S7.PDF]

Additional file 7 – Multiple alignments of non-coding DNA in 10-kb surrounding duplicate *F3´5´H* genes

In the panel on top of each page, *F3´5´H* exons are indicated as thick blue bars, introns are thin blue connectors. Coloured boxes indicate annotated TEs. Plots of sequence identity range from 50 to 100% on the *y*-axis in the LAGAN multi-panels.

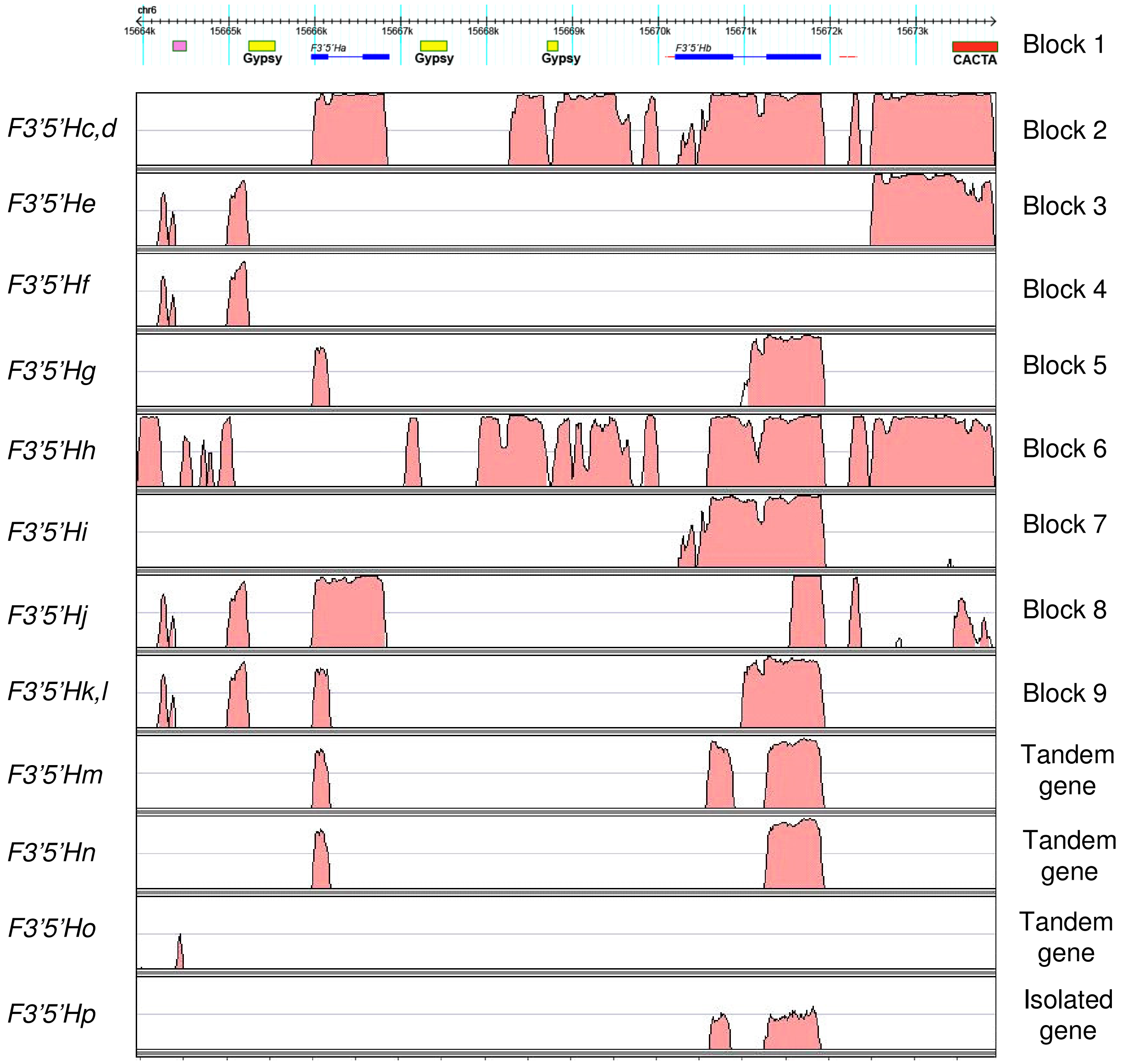

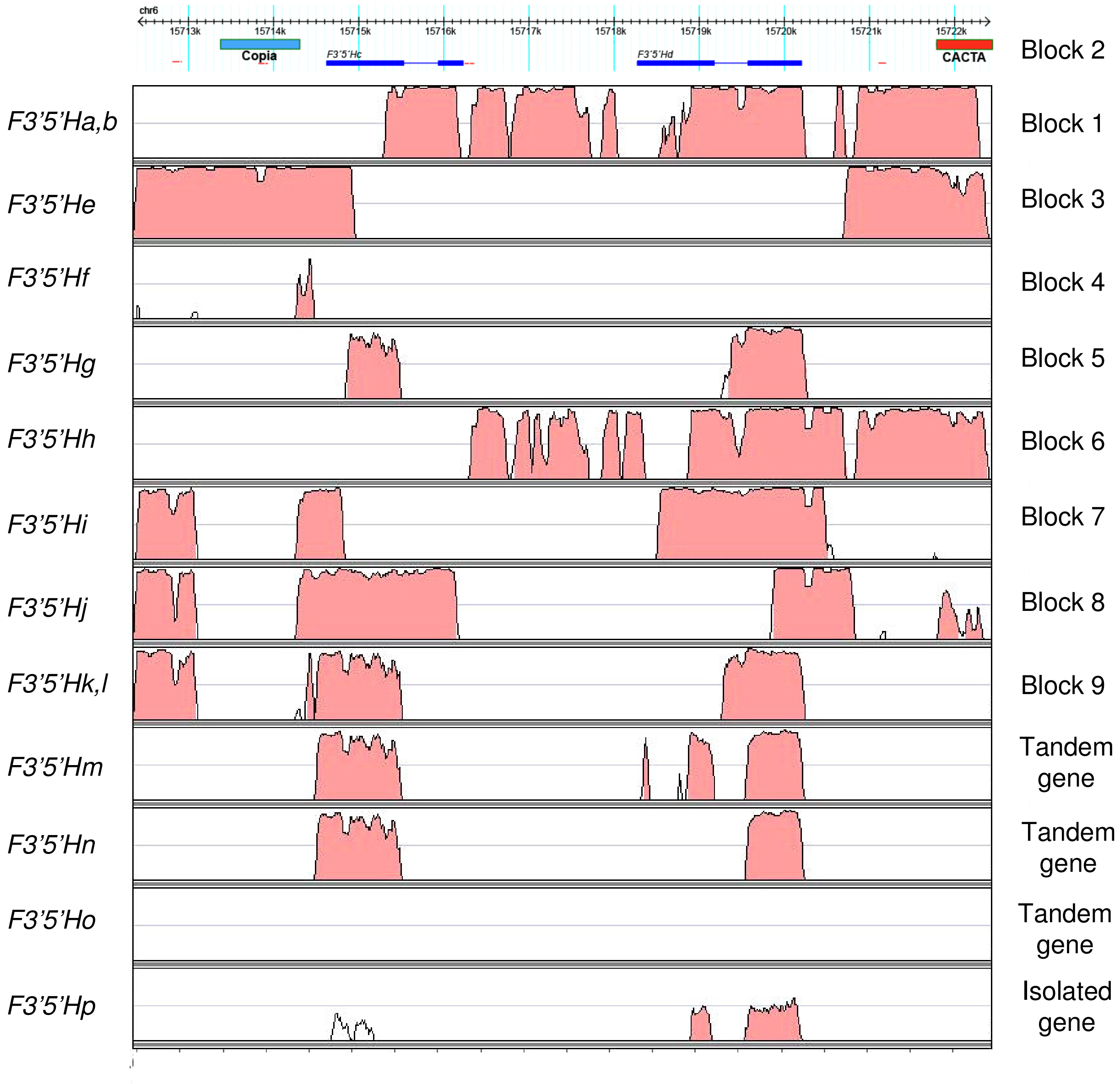
chr6
15713k
15714k
15715k
15716k
15717k
15718k
15719k
15720k
15721k
15722k
Copia
F3'5'Hc
F3'5'Hd
CACTA
Block 2
F3'5'Ha,b
Block 1
F3'5'He
Block 3
F3'5'Hf
Block 4
F3'5'Hg
Block 5
F3'5'Hh
Block 6
F3'5'Hi
Block 7
F3'5'Hj
Block 8
F3'5'Hk,l
Block 9
F3'5'Hm
Tandem gene
F3'5'Hn
Tandem gene
F3'5'Ho
Tandem gene
F3'5'Hp
Isolated gene

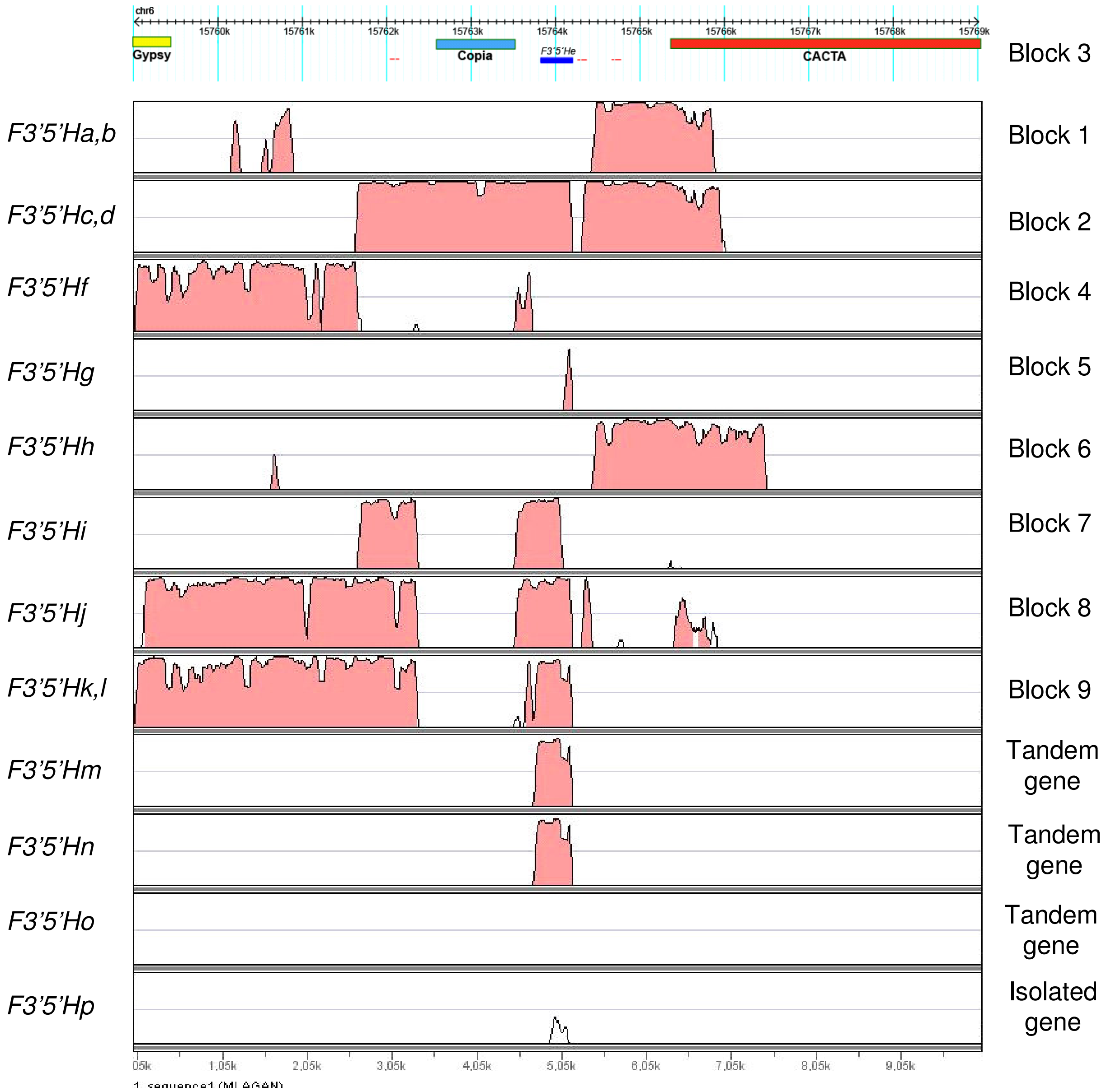

chr6
15760k
15761k
15762k
15763k
15764k
15765k
15766k
15767k
15768k
15769k
Gypsy
Copia
F3'5'He
CACTA
Block 3
F3'5'Ha,b
Block 1
F3'5'Hc,d
Block 2
F3'5'Hf
Block 4
F3'5'Hg
Block 5
F3'5'Hh
Block 6
F3'5'Hi
Block 7
F3'5'Hj
Block 8
F3'5'Hk,l
Block 9
F3'5'Hm
Tandem gene
F3'5'Hn
Tandem gene
F3'5'Ho
Tandem gene
F3'5'Hp
Isolated gene
05k
1,05k
2,05k
3,05k
4,05k
5,05k
6,05k
7,05k
8,05k
9,05k

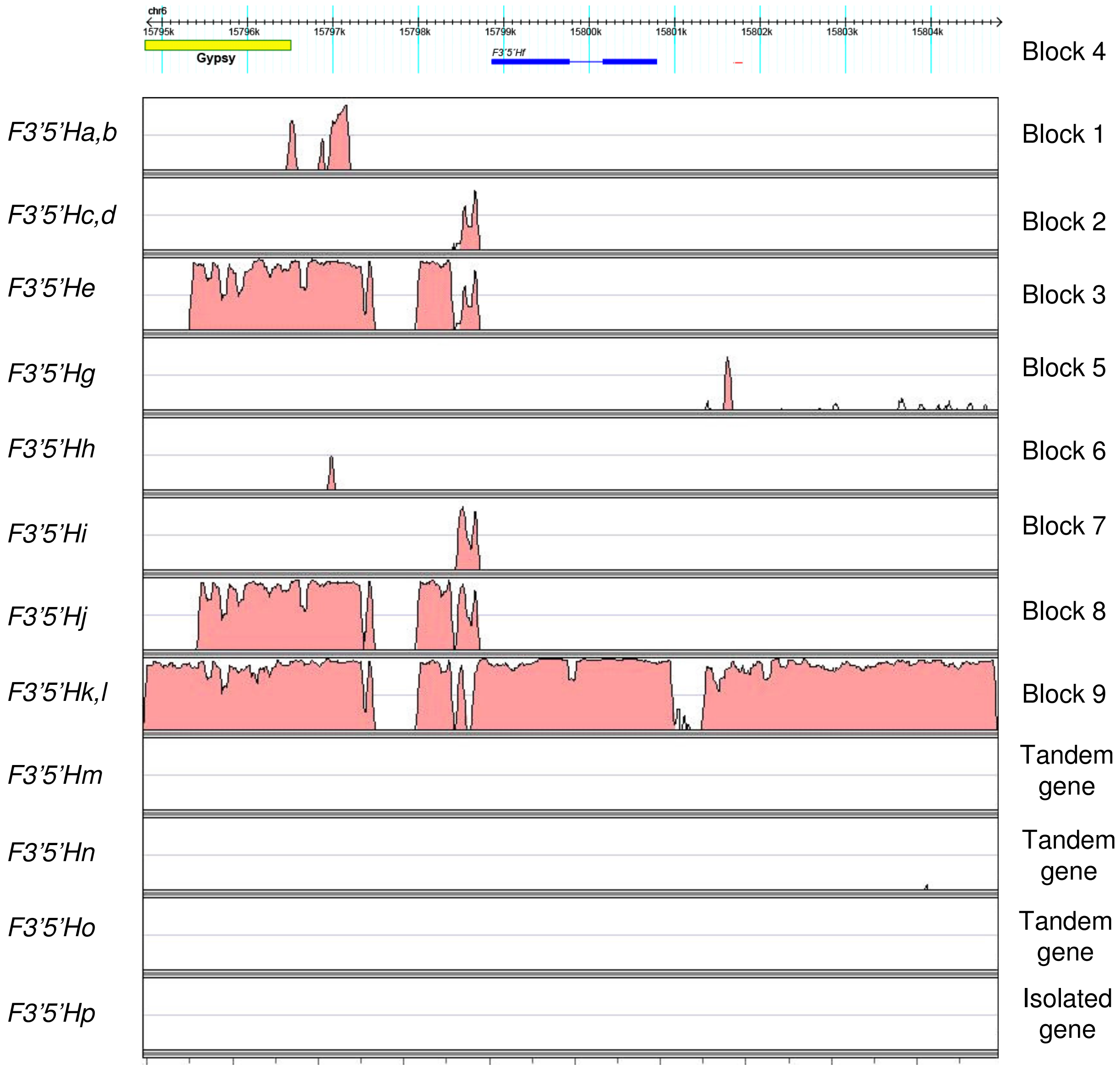
chr6
15795k
15796k
15797k
15798k
15799k
15800k
15801k
15802k
15803k
15804k
Gypsy
F3'5'Hf
Block 4
F3'5'Ha,b
Block 1
F3'5'Hc,d
Block 2
F3'5'He
Block 3
F3'5'Hg
Block 5
F3'5'Hh
Block 6
F3'5'Hi
Block 7
F3'5'Hj
Block 8
F3'5'Hk,l
Block 9
F3'5'Hm
Tandem gene
F3'5'Hn
Tandem gene
F3'5'Ho
Tandem gene
F3'5'Hp
Isolated gene

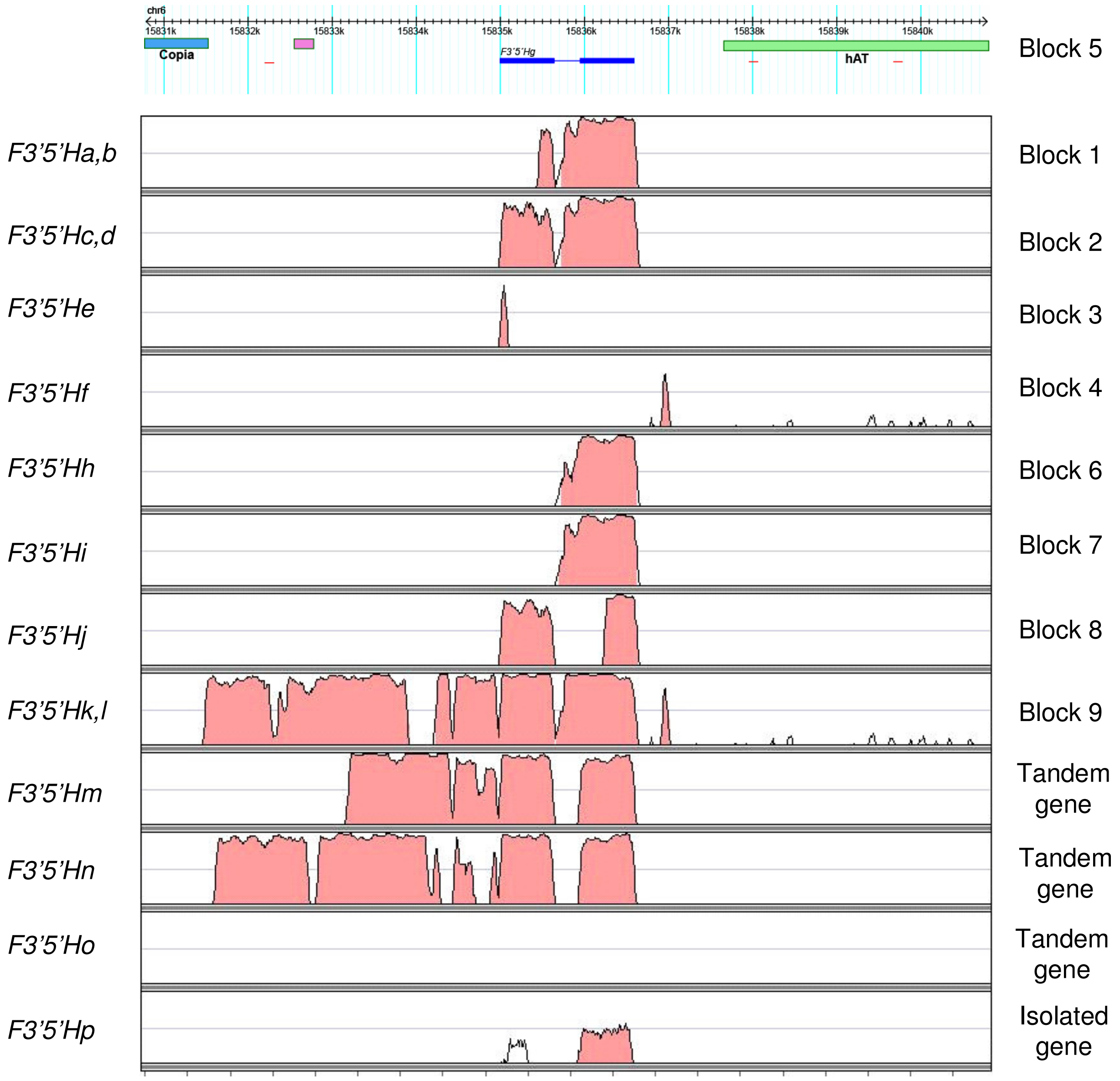
chr6
15831k
15832k
15833k
15834k
15835k
15836k
15837k
15838k
15839k
15840k
Copia
F3'5'Hg
hAT
Block 5
F3'5'Ha,b
Block 1
F3'5'Hc,d
Block 2
F3'5'He
Block 3
F3'5'Hf
Block 4
F3'5'Hh
Block 6
F3'5'Hi
Block 7
F3'5'Hj
Block 8
F3'5'Hk,l
Block 9
F3'5'Hm
Tandem gene
F3'5'Hn
Tandem gene
F3'5'Ho
Tandem gene
F3'5'Hp
Isolated gene

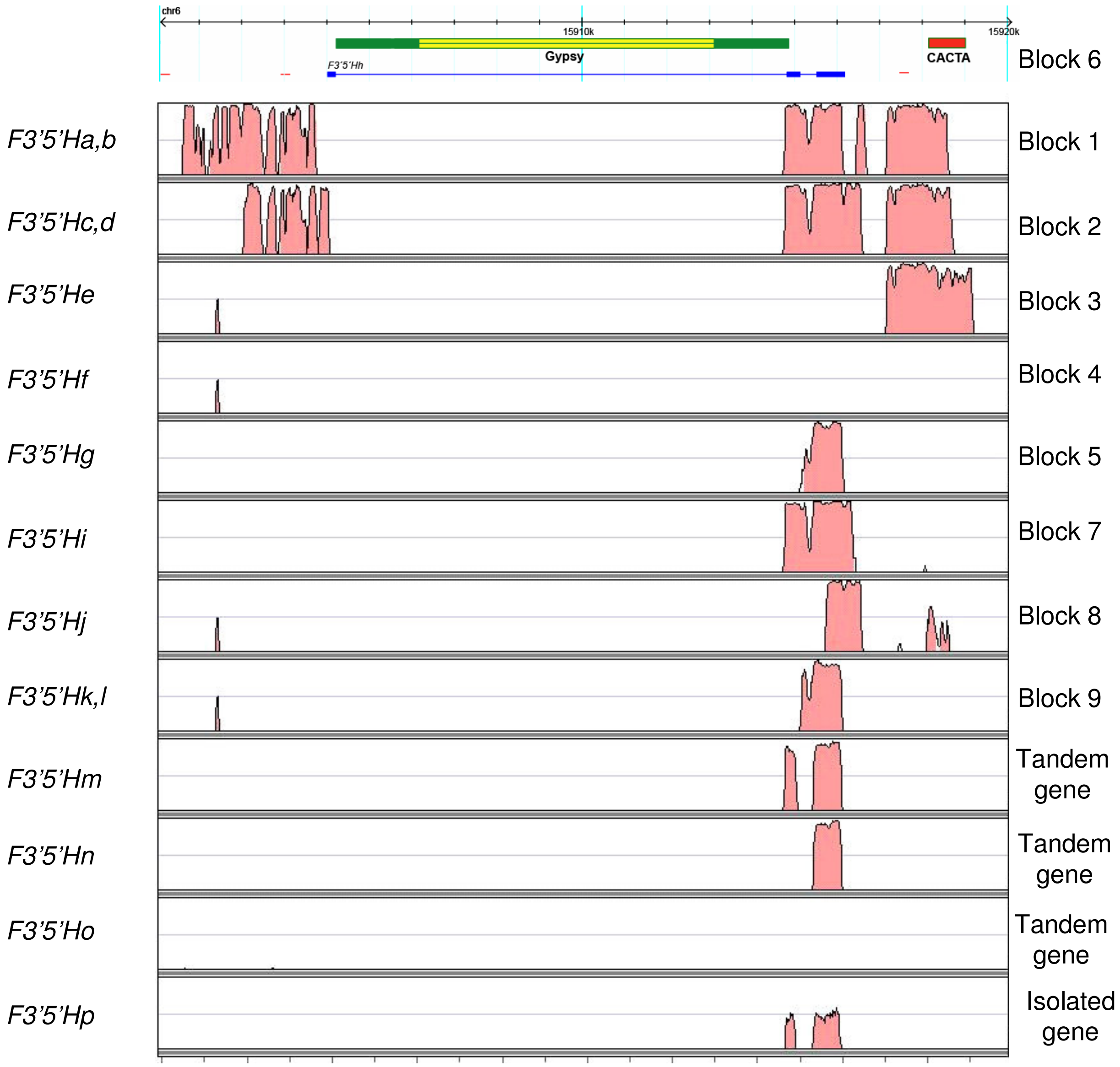

chr6
15910k
15920k
Gypsy
CACTA
F3'5'Hh
Block 6
F3'5'Ha,b
Block 1
F3'5'Hc,d
Block 2
F3'5'He
Block 3
F3'5'Hf
Block 4
F3'5'Hg
Block 5
F3'5'Hi
Block 7
F3'5'Hj
Block 8
F3'5'Hk,l
Block 9
F3'5'Hm
Tandem gene
F3'5'Hn
Tandem gene
F3'5'Ho
Tandem gene
F3'5'Hp
Isolated gene

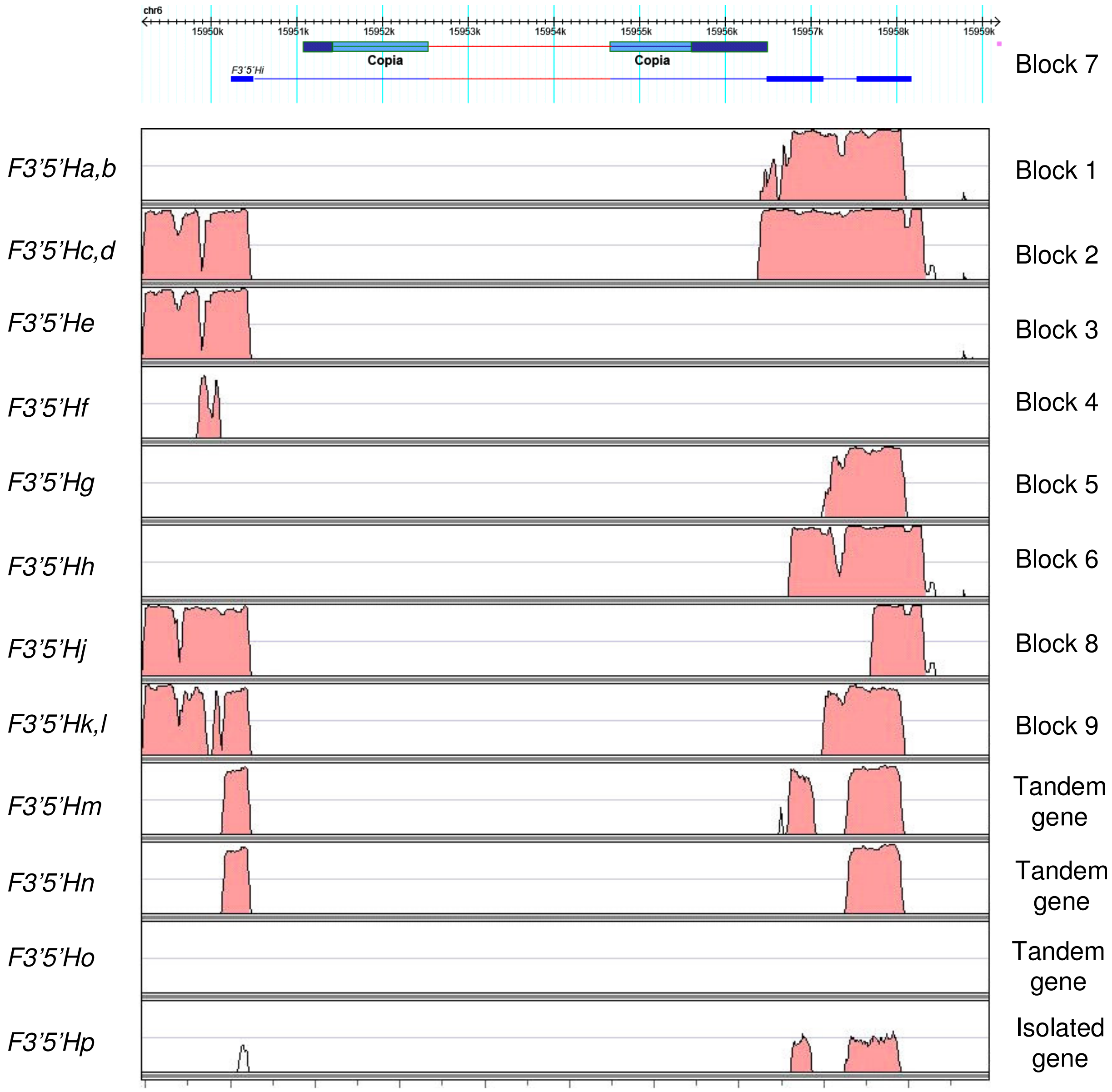

chr6
15950k
15951k
15952k
15953k
15954k
15955k
15956k
15957k
15958k
15959k
Copia
Copia
F3'5'Hi
Block 7
F3'5'Ha,b
Block 1
F3'5'Hc,d
Block 2
F3'5'He
Block 3
F3'5'Hf
Block 4
F3'5'Hg
Block 5
F3'5'Hh
Block 6
F3'5'Hj
Block 8
F3'5'Hk,l
Block 9
F3'5'Hm
Tandem gene
F3'5'Hn
Tandem gene
F3'5'Ho
Tandem gene
F3'5'Hp
Isolated gene

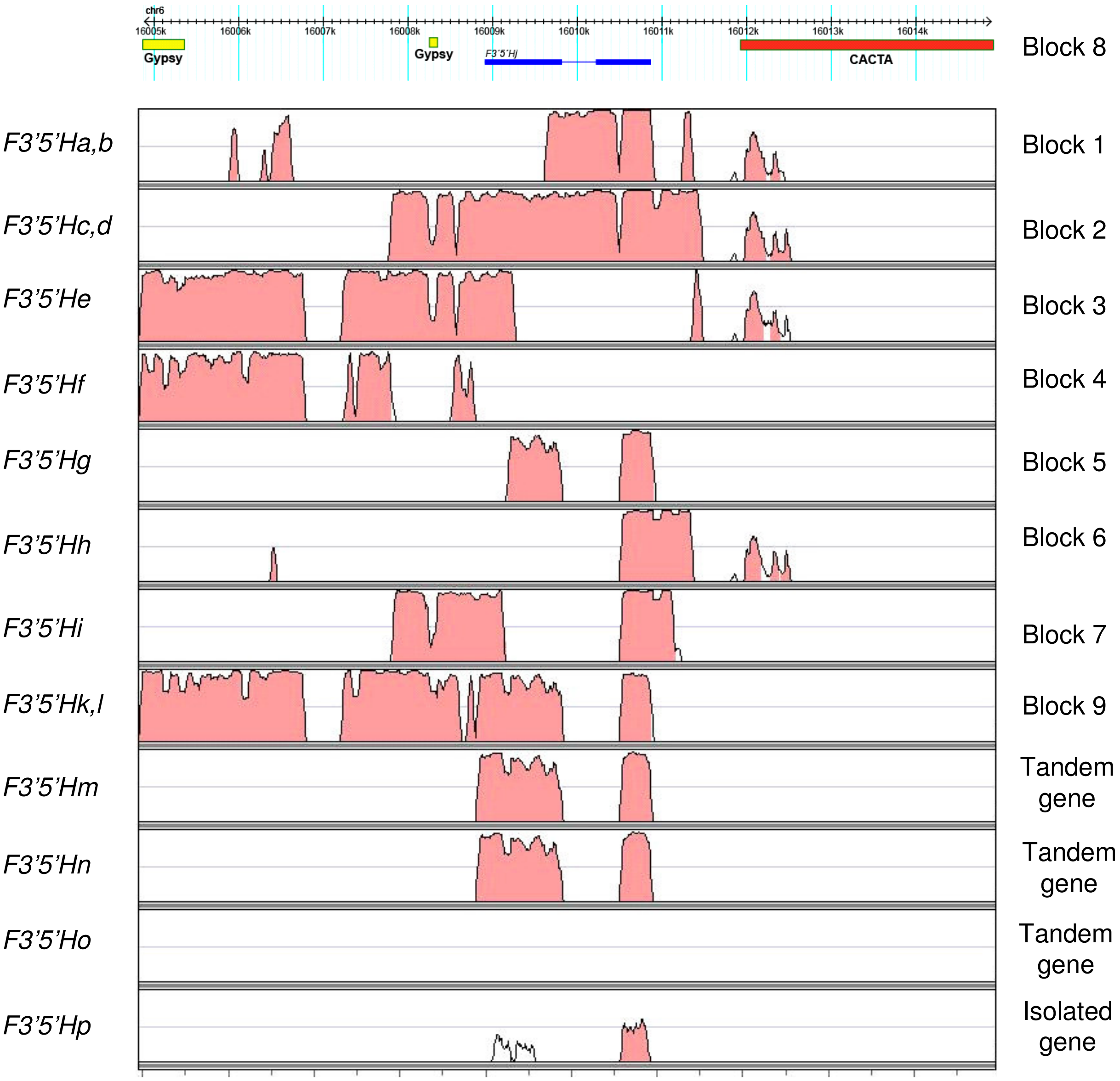
chr6
16005k
16006k
16007k
16008k
16009k
16010k
16011k
16012k
16013k
16014k
Gypsy
Gypsy
F3'5'Hj
CACTA
Block 8
F3'5'Ha,b
Block 1
F3'5'Hc,d
Block 2
F3'5'He
Block 3
F3'5'Hf
Block 4
F3'5'Hg
Block 5
F3'5'Hh
Block 6
F3'5'Hi
Block 7
F3'5'Hk,l
Block 9
F3'5'Hm
Tandem gene
F3'5'Hn
Tandem gene
F3'5'Ho
Tandem gene
F3'5'Hp
Isolated gene

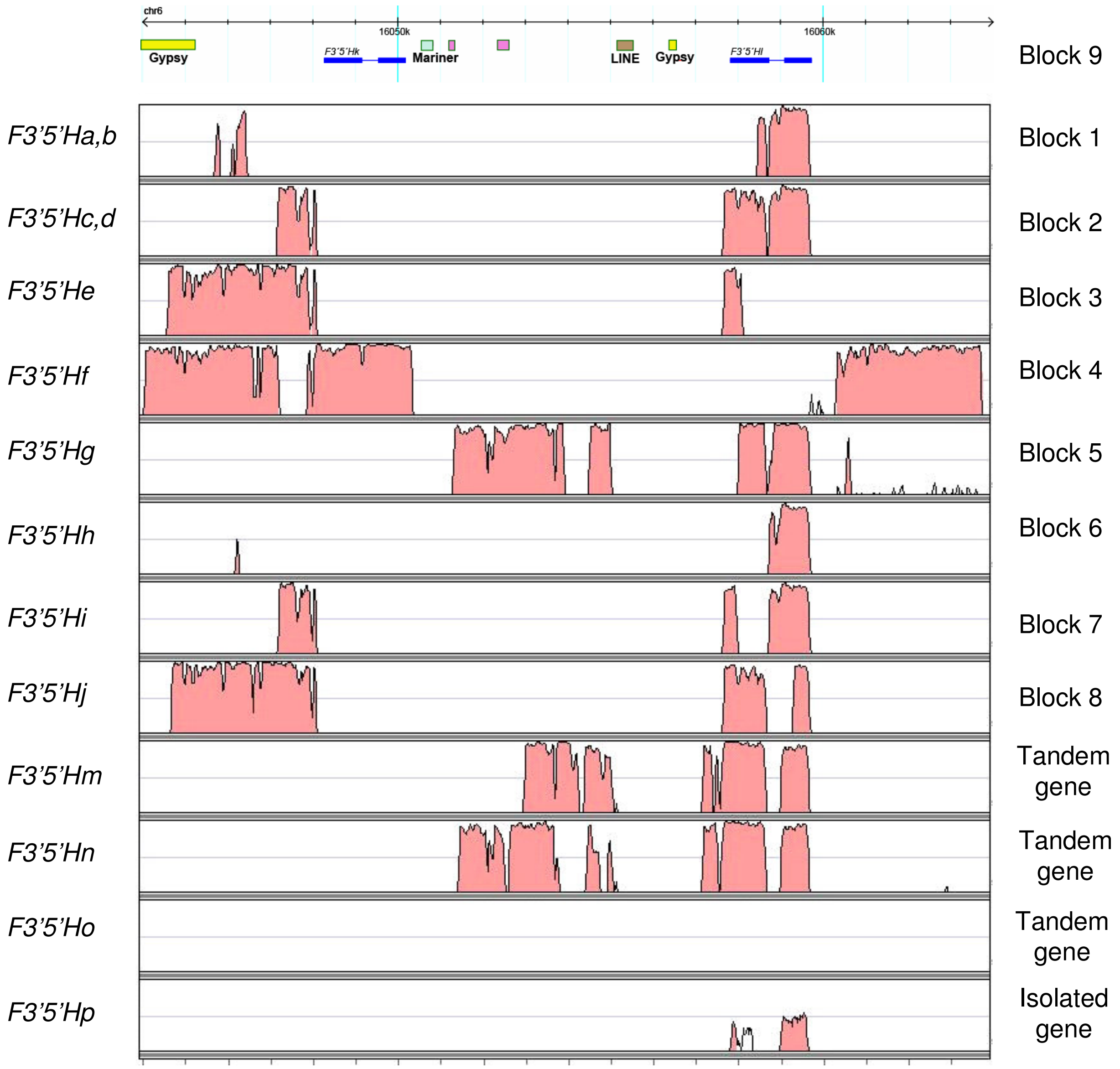

chr6
16050k
16060k
Gypsy
F3'5'Hk
Mariner
LINE
Gypsy
F3'5'Hl
Block 9
F3'5'Ha,b
Block 1
F3'5'Hc,d
Block 2
F3'5'He
Block 3
F3'5'Hf
Block 4
F3'5'Hg
Block 5
F3'5'Hh
Block 6
F3'5'Hi
Block 7
F3'5'Hj
Block 8
F3'5'Hm
Tandem gene
F3'5'Hn
Tandem gene
F3'5'Ho
Tandem gene
F3'5'Hp
Isolated gene

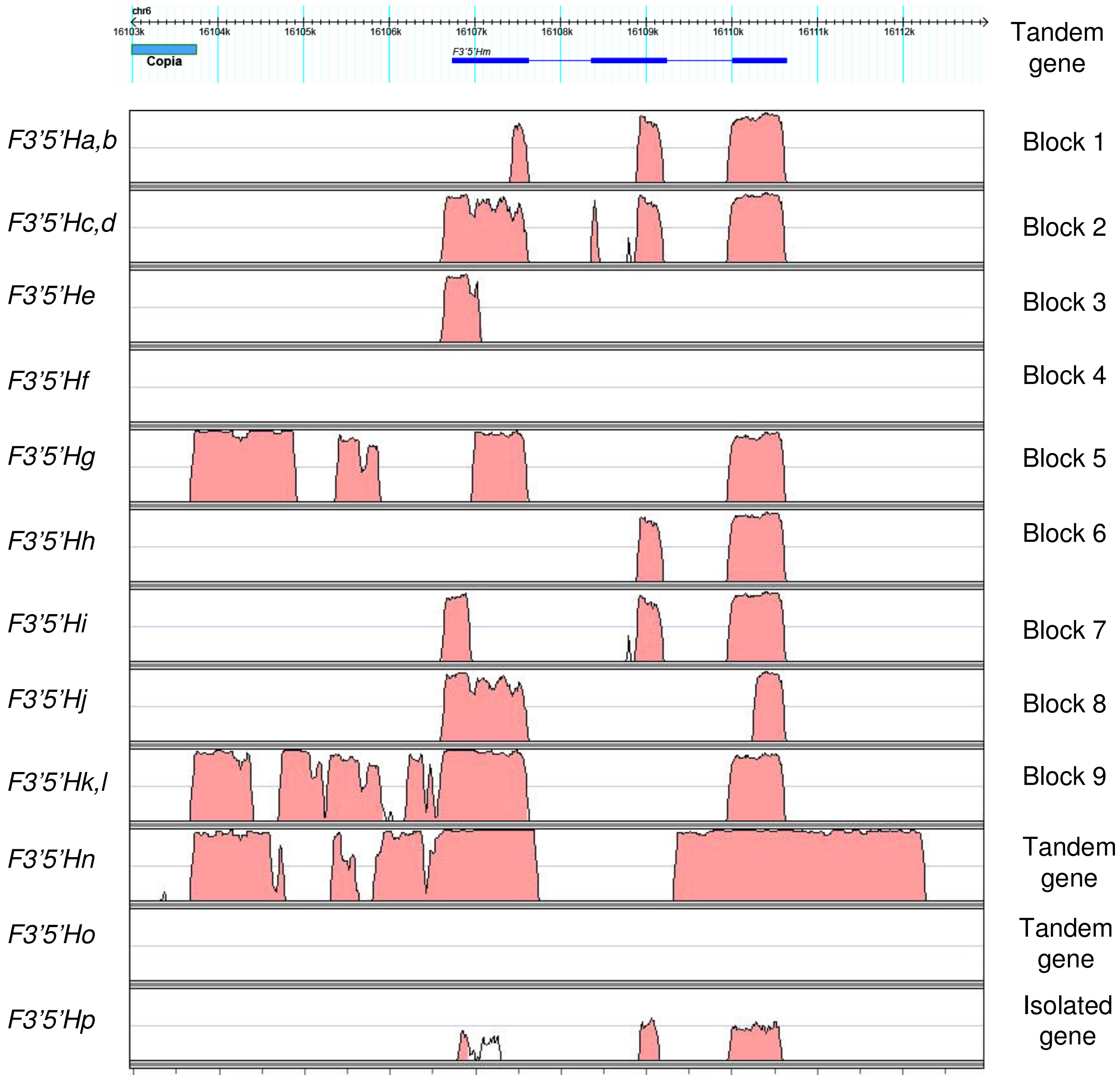
chr6
16103k
16104k
16105k
16106k
16107k
16108k
16109k
16110k
16111k
16112k
Copia
F3'5'Hm
Tandem gene
F3'5'Ha,b
Block 1
F3'5'Hc,d
Block 2
F3'5'He
Block 3
F3'5'Hf
Block 4
F3'5'Hg
Block 5
F3'5'Hh
Block 6
F3'5'Hi
Block 7
F3'5'Hj
Block 8
F3'5'Hk,l
Block 9
F3'5'Hn
Tandem gene
F3'5'Ho
Tandem gene
F3'5'Hp
Isolated gene

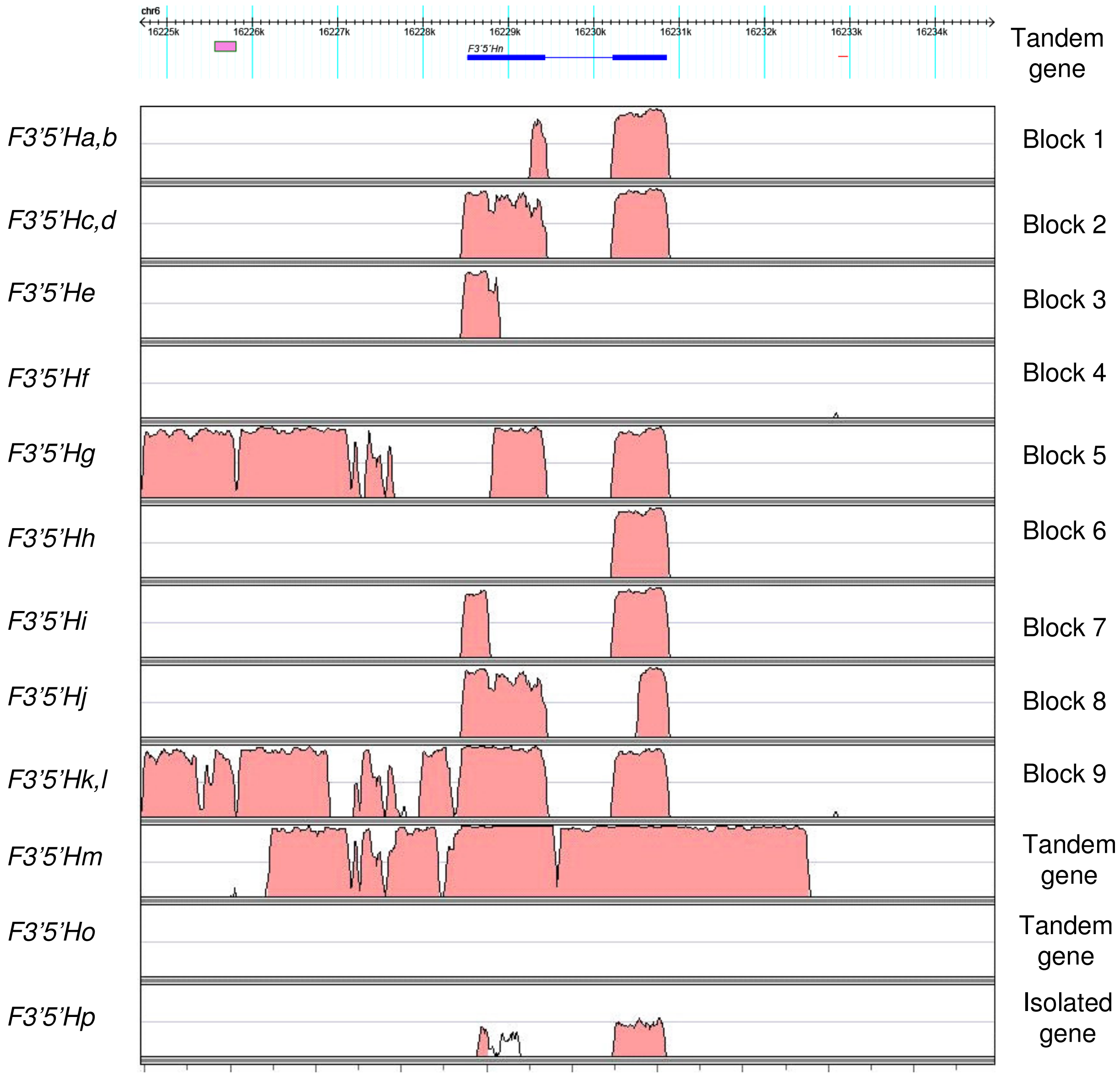
chr6
16225k
16226k
16227k
16228k
16229k
16230k
16231k
16232k
16233k
16234k
F3'5'Hn
Tandem gene
F3'5'Ha,b
Block 1
F3'5'Hc,d
Block 2
F3'5'He
Block 3
F3'5'Hf
Block 4
F3'5'Hg
Block 5
F3'5'Hh
Block 6
F3'5'Hi
Block 7
F3'5'Hj
Block 8
F3'5'Hk,l
Block 9
F3'5'Hm
Tandem gene
F3'5'Ho
Tandem gene
F3'5'Hp
Isolated gene

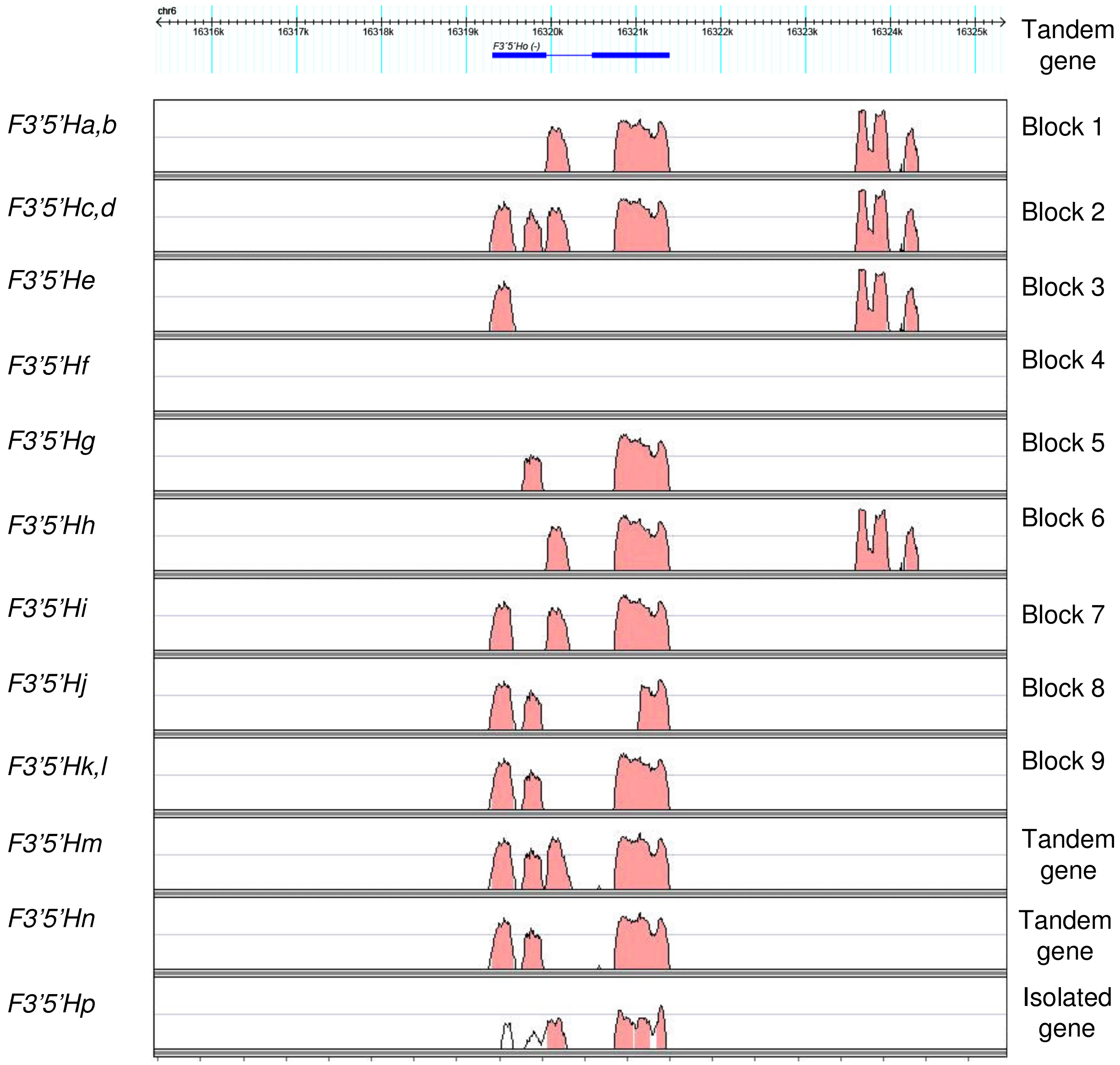
chr6
16316k
16317k
16318k
16319k
16320k
16321k
16322k
16323k
16324k
16325k
F3'5'Ho (-)
Tandem gene
F3'5'Ha,b
Block 1
F3'5'Hc,d
Block 2
F3'5'He
Block 3
F3'5'Hf
Block 4
F3'5'Hg
Block 5
F3'5'Hh
Block 6
F3'5'Hi
Block 7
F3'5'Hj
Block 8
F3'5'Hk,l
Block 9
F3'5'Hm
Tandem gene
F3'5'Hn
Tandem gene
F3'5'Hp
Isolated gene

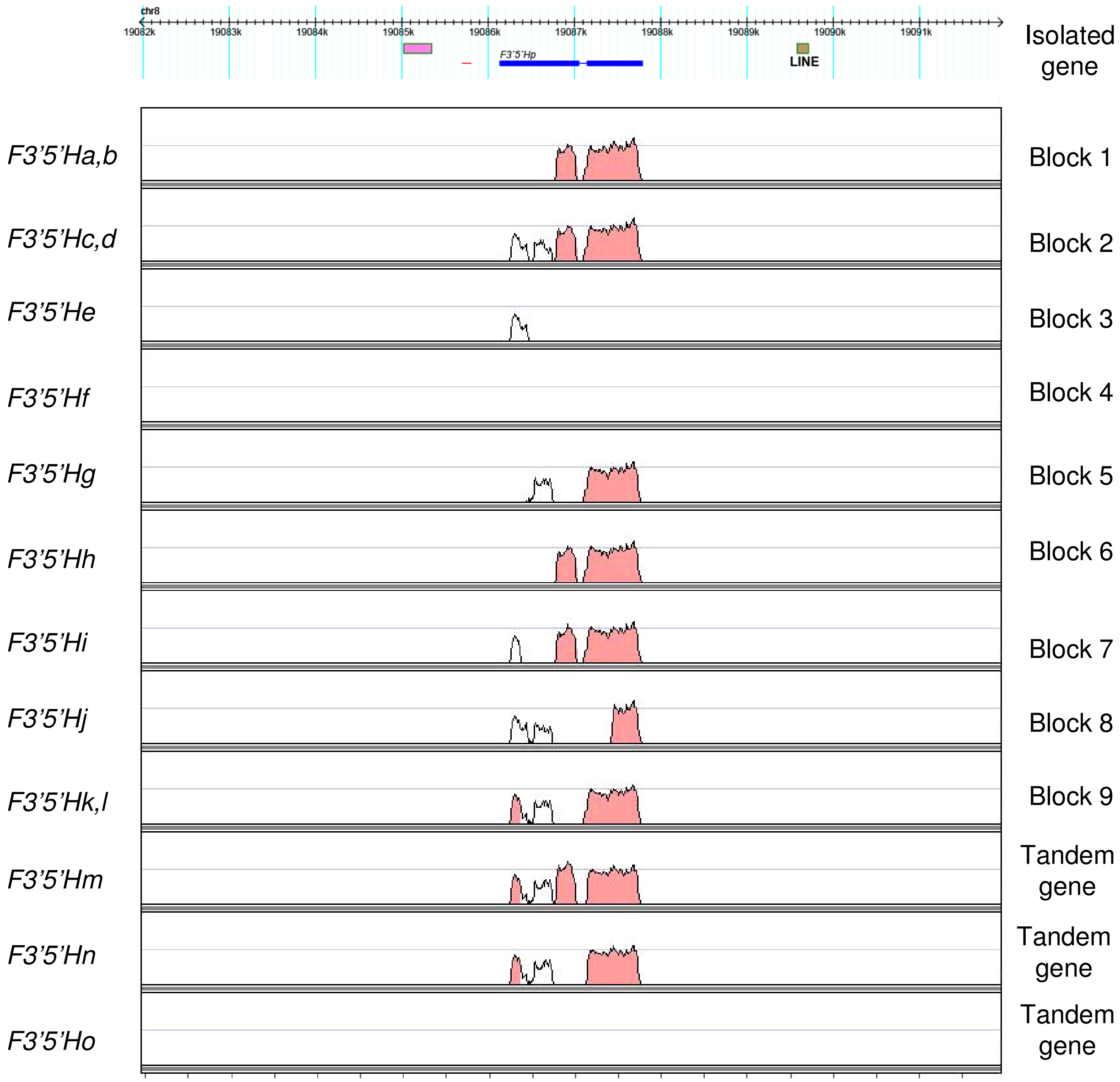
chr8
19082k
19083k
19084k
19085k
19086k
19087k
19088k
19089k
19090k
19091k
F3'5'Hp
LINE
Isolated gene
F3'5'Ha,b
Block 1
F3'5'Hc,d
Block 2
F3'5'He
Block 3
F3'5'Hf
Block 4
F3'5'Hg
Block 5
F3'5'Hh
Block 6
F3'5'Hi
Block 7
F3'5'Hj
Block 8
F3'5'Hk,l
Block 9
F3'5'Hm
Tandem gene
F3'5'Hn
Tandem gene
F3'5'Ho
Tandem gene
