# Supplementary material for: Expansion and subfunctionalisation of flavonoid 3',5'-hydroxylases in the grapevine lineage
Source: BMC Genomics. 2010 Oct 12;11:562. doi: 10.1186/1471-2164-11-562 (PMC3091711; doi:10.1186/1471-2164-11-562)
Supplement: Additional file 8 — Conservation and SSCP polymorphisms of duplicate F3'5'Hs in the family Vitaceae. PCR amplicons were obtained from genomic DNA using copy-specific primers. DNA samples included the ornamental grapevines Virginia creeper Parthenocissus quinquefolia, native to Northeastern-America, and the porcelain berry Ampelopsis brevipedunculata, native to temperate areas of Asia (segment A), wild grapevines (segment B) including the 2n = 40 Muscadinia rotundifolia, two North American species V. riparia and V. candicans, two Asian species V. armata and V. romanetii, and a spontaneous ecotype of V. vinifera ssp sylvestris collected in woods of Northeastern Italy; red-skinned cultivars of the domesticated V. vinifera ssp sativa (segment C); white-skinned cultivars (Pinot bud sports with mutations for skin colour are shown beside Pinot blanc) and the nearly-homozygous line PN40024 (segment D). PCR amplicons were run in agarose gel (section a) and in denaturing gel for detecting single-strand conformational polymorphisms (section b). Among F3'5'Hs, the isolated gene copies F3'5'Hp, -o, -m, and -n showed the lowest levels of conformational polymorphisms, while segmentally duplicated F3'5'Hs were more variable across taxa. [file 1471-2164-11-562-S8.PDF]

Additional file 8 – Conservation and SSCP polymorphisms of duplicate *F3´5´H*s in the family Vitaceae

PCR amplicons were obtained from genomic DNA using copy-specific primers. DNA samples included the ornamental grapevines Virginia creeper *Parthenocissus quinquefolia*, native to Northeastern-America, and the porcelain berry *Ampelopsis brevipedunculata*, native to temperate areas of Asia (segment A), wild grapevines (segment B) including the $2n = 40$ *Muscadinia rotundifolia*, two North American species *V. riparia* and *V. candicans*, two Asian species *V. armata* and *V. romanetii*, and a spontaneous ecotype of *V. vinifera* ssp *sylvestris* collected in woods of Northeastern Italy; red-skinned cultivars of the domesticated *V. vinifera* ssp *sativa* (segment C); white-skinned cultivars (Pinot bud sports with mutations for skin colour are shown beside Pinot blanc) and the nearly-homozygous line PN40024 (segment D). PCR amplicons were run in agarose gel (**section a**) and in denaturing gel for detecting single-strand conformational polymorphisms (**section b**). Among *F3´5´H*s, the isolated gene copies *F3´5´Hp, -o, -m, -n* showed the lowest levels of conformational polymorphisms; while segmentally duplicated *F3´5´H*s were more variable across taxa.

a

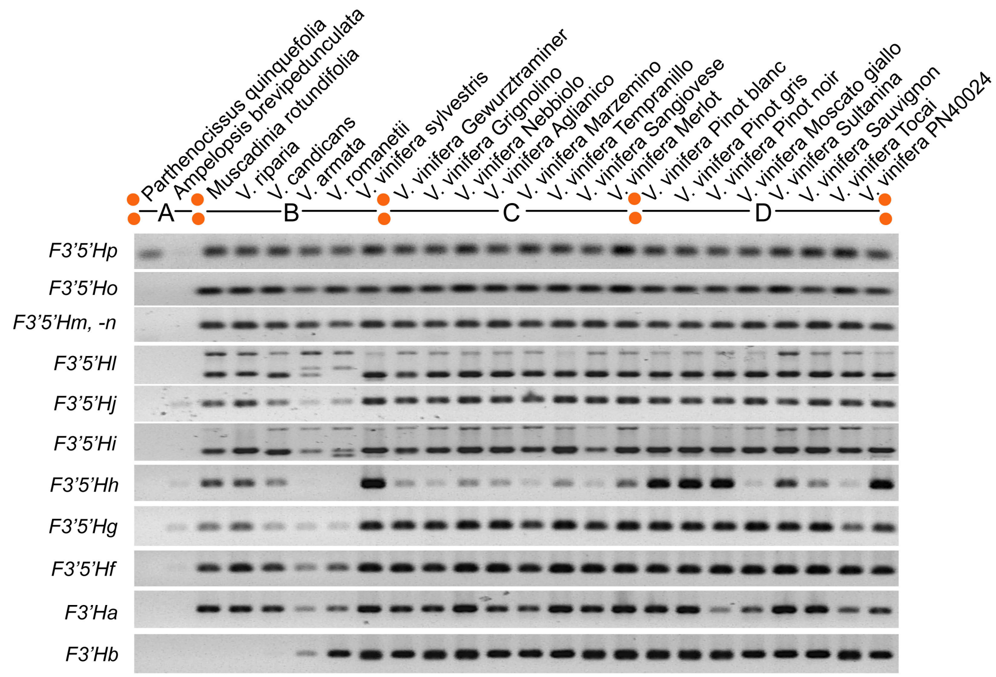

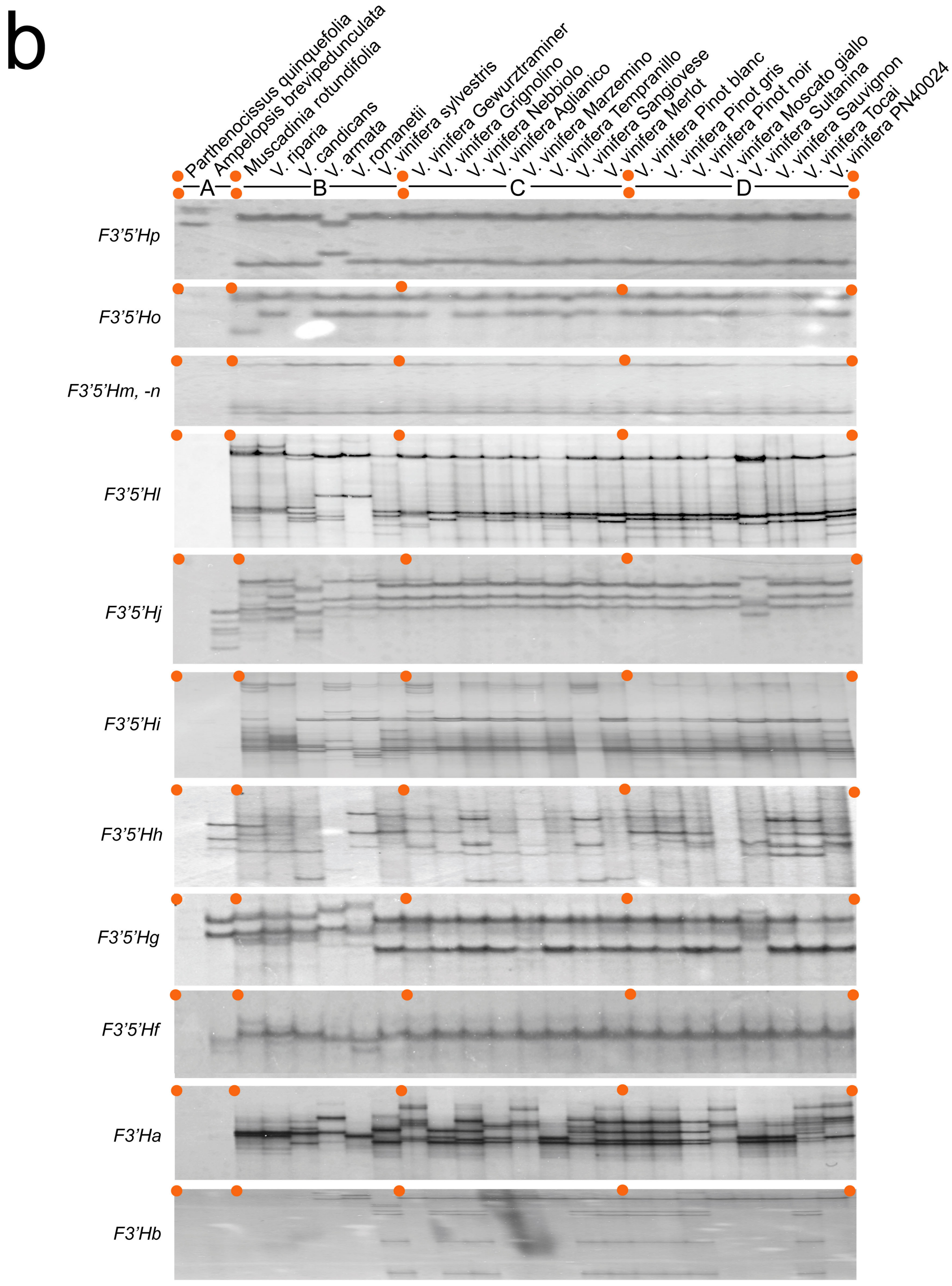

b
Parthenocissus quinquefolia
Ampelopsis brevipedunculata
Muscadinia rotundifolia
V. riparia
V. candicans
V. armata
V. romanetii
V. vinifera sylvestris
V. vinifera Gewurztraminer
V. vinifera Grignolino
V. vinifera Nebbiolo
V. vinifera Aglianico
V. vinifera Marzemino
V. vinifera Tempranillo
V. vinifera Sangiovese
V. vinifera Merlot
V. vinifera Pinot blanc
V. vinifera Pinot gris
V. vinifera Pinot noir
V. vinifera Moscato giallo
V. vinifera Sultanina
V. vinifera Sauvignon
V. vinifera Tocai
V. vinifera PN40024
A
B
C
D
F3'5'Hp
F3'5'Ho
F3'5'Hm, -n
F3'5'Hl
F3'5'Hj
F3'5'Hi
F3'5'Hh
F3'5'Hg
F3'5'Hf
F3'Ha
F3'Hb
