# Supplementary material for: Expansion and subfunctionalisation of flavonoid 3',5'-hydroxylases in the grapevine lineage
Source: BMC Genomics. 2010 Oct 12;11:562. doi: 10.1186/1471-2164-11-562 (PMC3091711; doi:10.1186/1471-2164-11-562)
Supplement: Additional file 9 — Amino acid alignment of substrate recognition sites (SRS) and functional domains for hydroxylation activity (CR1) in plant F3'5'Hs. Amino acid positions crucial for 3' vs. 3'5'-hydroxylation in CR1 and SRS6 are indicated by black arrows; significant amino acid substitutions in grapevine F3'5'Hs are in green background. Relevant amino acid substitutions within domains putatively involved in substrate recognition are highlighted in grapevine F3'5'Hs by blue background when they are unique with respect to all other plant F3'5'Hs or when they are shared exclusively with either monocot F3'5'Hs or other plant F3'Hs, as possible remnants of ancestral transition stages in the evolution of dicot F3'5'Hs. [file 1471-2164-11-562-S9.PDF]

Additional file 9 – Amino acid alignment of substrate recognition sites (SRS) and functional domains for hydroxylation activity (CR1) in plant *F3´5´Hs*

Amino acid positions crucial for 3´ vs. 3´,5´-hydroxylation in CR1 and SRS6 are indicated by black arrows; significant amino acid substitutions in grapevine *F3´5´Hs* are in green background. Relevant amino acid substitutions within domains putatively involved in substrate recognition are highlighted in grapevine *F3´5´Hs* by blue background when they are unique with respect to all other plant *F3´5´H*s or when they are shared exclusively with either monocot *F3´5´H*s or other plant *F3´Hs*, as possible remnants of ancestral transition stages in the evolution of dicot *F3´5´H*s.

	SRS1	CR1	SRS2	SRS4	SRS5	SRS6
Vitis_F35Ha	------------------------	----------	---------	LLLNLFTAGTDTSSSV	HPSTPLNLPR	---------
Vitis_F35Hh	------------------------	----------	VELMTTAGY	LLLNLFTAGTDTSSSV	HPSTPLNLPR	MDEAFGLAL
Vitis_F35Hc	NRPPNAGATLLAYHAQDMVFADY	ANMIGQVILS	VELMTTAGY	LLLNLFTAGTDTSSSV	HPSTPLNLPR	---------
Vitis_F35He	NRPPNAGATLLAYHAQDMVFAD-	----------	---------	----------------	----------	---------
Vitis_F35Hi	NRPPNAGASLLAYHAQDMVFADY	ANMIGQVILS	VELMTTAGY	LLLNLFTAGTDTSSSV	HPSTPLNLPR	MDEAFGLAL
Vitis_F35Hj	NRPPNAGATLLAYHAQDMVFADY	ANMIGQVILS	VELMTTAGY	LLLNLFTAGTDTSSSV	HPSTPLNLPR	MDEAFGLAL
Vitis_F35Hd	NRPPNAGATLLAYHAQDMVFADY	ANMIGQVILS	VELMTSAGY	LLLNLFTAGTDTSSSV	HPSTPLNLPR	MDEAFGLAL
Vitis_F35Hk	SRPPNAGATLLAYHAQDMVFADY	ANMIGQVILS	VELMTTAGY	LLLNLFTAGTDTSSSV	HPSTPLNLPR	MDEAFGLAL
Vitis_F35Hf	NRPPNAGATHLAYDAQDMVFADY	ANMIGQVILS	VELMTTAGY	LLLNLFTAGTDTSSSV	HPSTPLNLPR	MDEAFGLAL
Vitis_F35Hl	NRPPNAGATHLAYDAQDMVFADY	ANMIGQVILS	VELMTTAGY	LLLNLFTAGTDTSSSV	HPSTPLNLPR	MDEAFGLAL
Vitis_F35Hg	NRPPNAGATHLAYDAQDMVFADY	ANMIGQVILS	VELMTSAGY	LLLNLFTAGTDTSSSV	HPSTPLNLPR	MDEAFGLAL
Vitis_F35Hm	NRPPNAGATHLAYGAQDMVFADY	ANIIGQVILS	VELMTCAGY	LLQNLFAAGTDTSASI	HPSTPLNLPR	MDEAFGLAL
Vitis_F35Hn	NRPPNAGATHLAYGAQDMVFADY	ANIIGQVILS	VELMTCAGY	LLQNLFAAGTDTSASI	HPSTPLNLPR	MDEAFGLAL
Vitis_F35Hb	NRPPNAGATLLAYHAQDMVFADY	ANMIGQVILS	VELMTSAGY	LLLNLFTAGTDTSSSV	HPSTPLNLPR	MDEAFGLAL
Vitis_F35Ho	NREPGAATSHLVYGCQDMVFTEY	VNIISQTVLS	VESMVWAGQ	VLVNLFVAGTDTSSST	HPSTPLNLPR	MDEGFGLTL
Cammelia_F35H	NRPPNAGATHLAYGAQDMVFADY	ANMIGQVILS	VELMTTAGY	LLLNLFTAGTDTSSSI	HPSTPLNLPR	MDESFGLAL
Carica_F35H	NRPPNAGATHLAYDSQDMVFADY	ANMIGQVILG	VELMTTAGY	LLLNLFTAGTDTSSSI	HPSTPLNLPR	MEEVFGLAL
Glycine_F35H	NRPSNAGATHLAYDARDMVFAHY	ANMIGQVILS	VELMTVAGY	LLLNLFTAGTDTSSSI	HPSTPLNLPR	MEESFGLAL
Populus_F35Ha	NRPPNAGATHLAYNAQDMVFADY	ANMIGQIILS	VELMTSAGL	LLLNLFTAGTDTSSSI	HPSTPLNLPR	MDEVFGLAL
Populus_F35Hb	NRPIDGGPTHLAYNAQDMVFADY	ANMIGQIILS	VELMTSGGF	LLLDLFTAGTDTSSSV	HPSTPLNLPR	MDEVFGLAL
Gossypium_F35H	NRPSNAGATHIAYNSQDMVFAEY	ANMIGQVILS	VELMTSAGL	LLLNLFTAGTDTSSSI	HPSTPLNLPR	MDEAFGLAL
Viola_F35H	NRPPNAGASHLAYGAQDLVFAKY	ANMIGQVILS	VELMTSAGY	VLLNLFTAGTDTSSSI	HPSTPLNLPR	MDESFGLAL
Rhododendron_F35H	NRPPNAGATHLAYNSQDMVFADY	ANIIGQVILG	VELMTSAGL	LLLNLFTAGTDTSSSV	HPSTPLNLPR	MDESFGLAL
Eustoma_F35H	NRPPNAGATHLAYNAQDMVFADY	ANMLGQVMLS	VELMTVAGY	LLLNMFTAGTDTSSSV	HPSTPLNLPR	MDEPFGLAL
Gentiana_F35H	NRPPNAGATHLAYNAQDMVFAHY	ANMLSQVILS	VELMTSAGY	LLLNLFTAGTDTSSSI	HPSTPLNLPR	MDETFGLAL
Vinca_F35H	NRPPNAGATHLAYGAQDMVFAHY	ANMIGQVILS	VELMTSAGL	LLLNLFSAGTDTSSSI	HPSTPLNLPR	MDESFGLAL
Nierembergia_F35H	NRPPNAGATHFAYNAQDMVFAPY	ANMIGQVILS	VELMTVAGY	LLLNLFTAGTDTSSSV	HPSTPLNLPR	MEEVFGLAL
Petunia_F35H	NRPPNAGATHLAYNAQDMVFAHY	ANMIGQVMLS	VELMTIAGY	LLLNLFTAGTDTSSSA	HPSTPLNLPR	MEEAFGLAL
Solanum_m_F35H	NRPPNAGATHMAYNAQDMVFAPY	ANMIGQVMLS	VELMTVAGY	LLLNLFTAGTDTSSSV	HPSTPLNLPR	MEETFGLAL
Solanum_t_F35H	NRPPNAGATHLAYNAQDMVFAPY	ANMIGQVMLS	VELMTVAGY	LLLNLFTAGTDTSSSV	HPSTPLNLPR	MEESFGLAL
Lycianthes_F35H	NRPPNAGATHLAYDAQDMVFAHY	ANMIGQVILS	VELMTVAGY	LLLNLFTAGTDTSSSA	HPSTPLNLPR	MEESFGLAL
Torenia_F35H	NRPANAGATHLAYGAQDMVFAPY	ANMIGQVILS	VELMTSAGY	LLLNLFTAGTDTSSST	HPSTPLNLPR	MEEAFGIAL
Verbena_F35H	DRPPNAGATLLAYNAQDMVFAHY	ANMIGQVILS	VELMTSAGY	LLLNLFTAGTDTSSST	HPSTPLNLPR	MEEAFGLAL
Campanula_F35H	NRPIDGGPTYLAYNAQDMVFAEY	ANMIGRIILS	MELMRMAGL	LLLDLFTAGTDTSSSV	HPSTPLNLPR	MEESFGIAL
Lobelia_F35H	NRPLEGGPTHLAYNAQDMVFANY	ANMLGQVIFS	MELMRVAGL	LILDLVTAGTDTSSSI	HPSTPLSLPR	MEESFGIAL
Delphinium_F35H	NRPTDAGATHIAYNSQDMVFADY	ANMLGQVILS	IELMTSAGL	LLLNLFSAGTDTSSST	HPSTPLNLPR	MEETFGIAL
Vitis_F35Hp	NRPPNAGATHIAYEAQDFVFADI	ANIIGQKSLS	VELMRLAGL	LLLNLFIAGTDTSSGT	HPSVPLNIPR	MDEAFGLVL
Picea_F35H	NRPGNAGATYIAYDSQDMVWAAY	ANMIGQIILS	VELMTSAGY	LLLNLFTAGTDTSSSV	HPSTPLSLPR	MDEAFGLAL
Phalaenopsis_F35H	DRPLDIISKQVSYNGQNMVFADY	ANVIGRISMS	KELLVGQGA	FISDLFVAGTDTSAMV	HPPTPLSIPH	MEEGPGLVL
Dendrobium_F35H	DRPSGVISKEISYNGQNMVFADY	ANVIGRIAMS	KELLVGQGA	FISDLFVAGTDTSAIV	HPPTPLSIPH	MEEGPGLVL
Sorghum_F35Ha	NRPAVASAADITYGCQNMVFANY	ANIVGQITVS	VSLLTGAGL	LIFDMFTAGTDTSSII	HPSTPLSLPH	MRETFGLTL
Zea_F35H	NRPAVASTADITYGCQNMVFANY	ANIVGQITVS	VSLMTGTGL	LIF-------------	HPSTPLSLPH	MRETFGLTV
Sorghum_F35Hb	NRPAVASAADITYRRQNMVFADY	ANIVGQITVS	VSLLTGTGM	LIFDMFTAGTDTSSII	HPSTPLSLPH	MSETFGLAL
Oryza_F35H	NRPAVASAVDITYNYQNMVFANY	ANIVGQITVS	VSLLTGAGL	LIFDMFTAGTDTSSII	HPSTPLSLPH	MSETFGLAL
Vitis_F3Ha	NRPPNSGAKHIAYNYQDLVFAPY	TNALGRVMLG	VELMVLAGV	LLLNLFTAGTDTSSST	HPSTPLSLPR	MDEAYGLTL
Vitis_F3Hb	NRPPNSGAKHIAYNYQDLVFAPY	TNALGRVMLG	VELMVLAGV	LLLNLFTAGTDTSSST	HPSTPLSLPR	MDEAYGLTL
Populus_F3H	SRPPNSGAKHIAYNYQDLVFAPY	ANALGRVMLG	VEVMVLAGV	LLLNMFTAGTDTSSST	HPSTPLSLPR	MDEAYGLTL
Hieracium_F3H	SRPPNSGAKHMAYNYQDLVFAPY	TNALVRVMLG	IEMMVLAGE	LLLNLFVAGTDTSSST	HPSTPLSLPR	MEEAYGLTL
Osteospermum_F3H	SRPPNSGAKHIAYNYQDLVFAPY	TNALARVMLG	VEVMELAGE	LLLNLFTAGTDTSSST	HPSTPLSLPR	MEEAYGLTL
Gerbera_F3H	DRPPNSGAKHIAYNYQDLVFAPY	TNALARVMLG	VELMVLAGE	LLLNLFTAGTDTSSST	HPSTPLSLPR	MEEAYGLTL
Lobelia_F3H	NRPPNSGAVHIAYNYQDLVFAPY	TNALSRVMFG	VEAMVLAGV	LLLNMFIAGTDTSSST	HPSTPLSLPR	MEEGYGLTL
Ipomoea_F3H	NRPPNSGAEHIAYNYQDLVFAPY	TNALARVMLG	EEMMVLAGS	LLLNLFTAGTDTSSST	HPSTPLSLPR	MEEAYGLTL
Petunia_F3H	SRPPNSGAEHMAYNYQDLVFAPY	TNALARVMLG	VEMMVVAGV	LLLNLFVAGTDTSSST	HPSTPLSLPR	MEEAYGLTL
Gentiana_F3H	NRPPNSGAKHIAYNYQDLVFAPY	TNALARVMLG	VEIMVLAGA	LLLNLFIAGTDTSSST	HPSTPLSLPR	MEEGYGLTL
Torenia_F3H	NRPPNSGAKHIAYNYQDLVFAPY	TNALARVMLG	VEIMVLAGA	LLLNLFIAGTDTSSST	HPSTPLSLPR	MEEGYGLTL
Perilla_F3H	SRPPNSGAEHIAYNYNDLVFAPH	TNAISRVMLG	VELMALAGV	LLLNLFTAGTDTTSST	HPSTPLSLPR	MEEAYGITL
Arabidopsis_F3H	SRPPNSGAKHMAYNYQDLVFAPY	VNALGREMIG	TEMMALAGV	LLLNMFTAGTDTSAST	HPPTPLSLPH	MEESYGLTL
Matthiola_F3H	SRPPNSGAKHIAYNYQDLVFAPY	VNALGREMIG	TEMMALAGV	LLLNMFTAGTDTSAST	HPPTPLSLPH	MEETYGITV
Carica_F3H	SRPPNSGAKHIAYNYQDLVFAPY	VNALGQVMLG	VEAMLLAGV	LLLNMFIAGTDTSSST	HPSTPLSLPR	MDEAYGLTL
Glycine_F3H	SRPPNAGAKYIAYNYQDLVFAPY	TNALARAMIG	MEVMVLAGV	LLLNMFTAGTDTSSST	HPSTPLSVPR	MDEAYGLTL
Pelargonium_F3H	SRPPNAGAKHIAYNYHDLVFAPY	TNALGRAVIG	VELMVLAGV	LLLNMFTAGTDTTSST	HSSTPLSLPR	MDEAYGLTL
Allium_F3H	NRPPNSGAEHIAYNYQDLVFAPY	TNALSKAMMG	LEIMSLAGV	LLLNLFTAGTDTSSST	HPSTPLSLPR	MEEAYGLPL
Tricyrtis_F3H	NRPPNSGAEHIAYNYQDLVFAPY	TNALARATVG	LEIMKLAGV	LLLNLFTAGTDTTSST	HPSTPLSLPR	MEESYGLTL
Sorghum_F3Ha	NRPPNSGAEHVAYNYQDLVFAPY	TNTLARATVG	VELMQLAGV	LLLNLFTAGTDTTSST	HPSTPLSLPR	MEEAYGLTL
Sorghum_F3Hb	NRPPNSGAEHMAYNYQDMVFAPY	TNTLARATVG	VELMQLAGV	LLLDLFTAGTDTTSST	HPSTPLSLPR	MEEAYGLTL
Zea_F3Ha	NRPPNSGAEHVAYNYQDLVFAPY	TNTLARATVG	VELMQLAGV	LLLNLFTAGTDTTSST	HPSTPLSLPR	MEEAYGLTL
Oryza_F3Ha	NRPPNSGAEHVAYNYQDLVFAPY	TNTLARATIG	VELMQLAGV	LLLNLFTAGTDTTSST	HPSTPLSLPR	MEEAYGLTL
Sorghum_F3Hc	CRPPNSGGELMAYNYQDVVFAPY	TNALSRAAVG	LEVMEVGGV	LILNLFIAGTDTTSTI	HPSTPLSLPR	MEEAFTLLL
Zea_F3Hb	SRPPNSGGELMAYNYQDVVFAPY	TNALSRAAVG	LEVMQVGGV	LILNLFVAGTDTTSTI	HPSTPLSLPR	MEEAFTLLL
Oryza_F3Hb	SRPRNSGGEHMAYNGRDVVFGPY	TNALSRAAVG	LEVMEVGGV	LILNLFVAGTDTTSTI	HPSTPLSLPR	MDEAFTLLL
