# Supplementary material for: Expansion and subfunctionalisation of flavonoid 3',5'-hydroxylases in the grapevine lineage
Source: BMC Genomics. 2010 Oct 12;11:562. doi: 10.1186/1471-2164-11-562 (PMC3091711; doi:10.1186/1471-2164-11-562)

Additional file 11 - Expression of duplicate *F3´5´H*s in berry skin of four cultivars accumulating 3´5´-OH anthocyanins detected by semiquantitative PCR

Berry skin was sampled at four developmental stages. cDNA was normalised using the housekeeping *Ubiquitin* gene. *UFGT* was used as a marker for anthocyanin gene expression. Even though the preveraison berries were sampled over green bunches immediately before visible colour transition, expression of *UFGT* had already been triggered in 'Aglianico' and barely detectable in 'Nebbiolo'. Either primer of the oligonucleotide pairs targeting the *F3´5´Hi* and *F3´5´Hl* copies anneals to either exon of the corresponding gene model. The corresponding PCR bands obtained from gDNA are approximately 400 bp longer than the cDNA amplicons shown in the stripes of the electrophoresis gel of this figure.

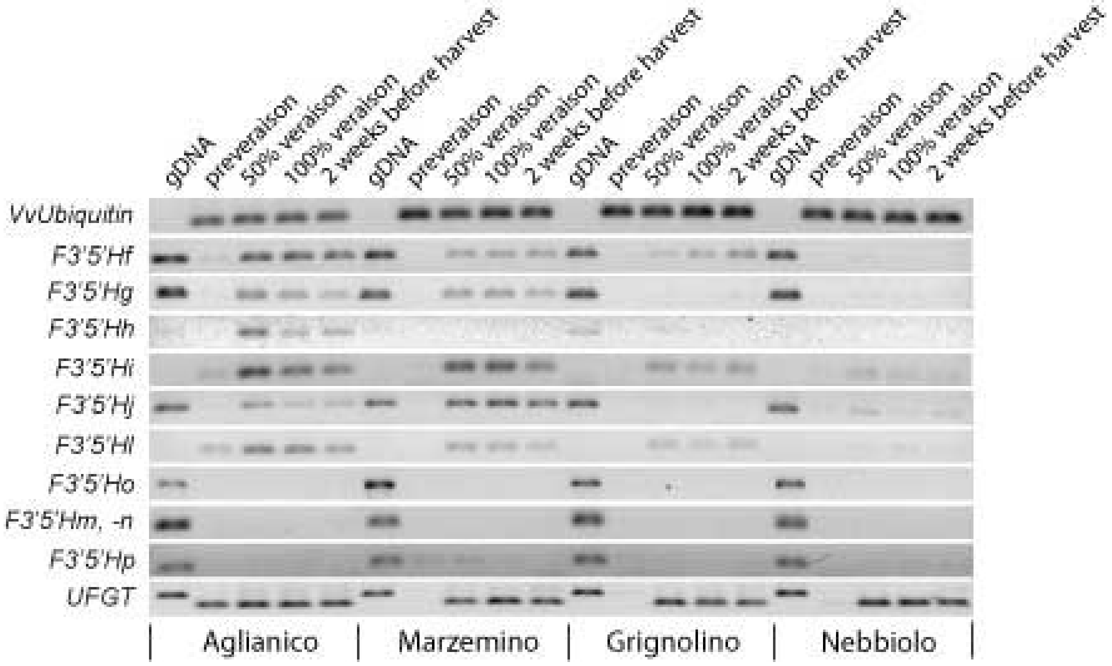

Supplement: Additional file 11 — Expression of duplicate F3'5'Hs in berry skin of four cultivars accumulating 3'5'-OH anthocyanins detected by semiquantitative PCR. Berry skin was sampled at four developmental stages. cDNA was normalised using the housekeeping Ubiquitin gene. UFGT was used as a marker for anthocyanin gene expression. Even though the pre-veraison berries were sampled over green bunches immediately before visible colour transition, expression of UFGT had already been triggered in 'Aglianico' and was barely detectable in 'Nebbiolo'. Either primer of the oligonucleotide pairs targeting the F3'5'Hi and -l copies anneals to either exon of the corresponding gene model. The corresponding PCR bands obtained from gDNA are approximately 400 bp longer than the cDNA amplicons shown in the stripes of the electrophoresis gel of this figure. [file 1471-2164-11-562-S11.PDF]
