# Supplementary material for: Expansion and subfunctionalisation of flavonoid 3',5'-hydroxylases in the grapevine lineage
Source: BMC Genomics. 2010 Oct 12;11:562. doi: 10.1186/1471-2164-11-562 (PMC3091711; doi:10.1186/1471-2164-11-562)
Supplement: Additional file 12 — Analysis of variance of duplicate F3'5'H expression in berry skin of four cultivars along eight developmental stages. [file 1471-2164-11-562-S12.PDF]

Additional file 12 – Analysis of variance of duplicate *F3´5´H* expression in berry skin of four cultivars along eight developmental stages

Three-way ANOVA

1st Factor: gene-copy
2nd Factor: cultivar
3rd Factor: developmental stage

Source	df	Type III SS	MS	F	P	
Main effects						
gene-copy	6	0.028	0.004	63,156	.0000	***
cultivar	3	0.022	0.007	101,564	.0000	***
time	7	0.010	0.001	204,934	.0000	***
Interaction						
gene-copy × cultivar	18	0.062	0.003	460,366	.0000	***
gene-copy × stage	42	0.029	7,06E-01	938,865	.0000	***
cultivar × stage	21	0.014	7,02E+00	933,655	.0000	***
gene-copy × cultivar × stage	126	0.074	5,89E+00	783,533	.0000	***
Error	445	0.033	7.5219e-5			
Total	668	0.277				
Model	223	0.244	0.001	14554299,00	.0000	***

R^2 = SSmodel/SStotal = 0.879
Root MSerror = sqrt(MSerror) = 0.008
Mean Y = 0.005
Coefficient of Variation = 161.7%

Compare Means: **Ranking cultivars for global abundance of the *F3´5´H* transcript pool**
Factor: cultivar
Test: Student-Newman-Keuls
Significance Level: 0.05
Variance: 7.521e-5
LSD 0.05 = 0.001

Rank	Cultivar	Mean	n	Non-significant ranges
1	Aglianico	0.015	167	a
2	Marzemino	0.004	168	b
3	Grignolino	8.161e-4	167	c
4	Nebbiolo	6.912e-4	167	c

Compare Means: **Transcript abundance of the *F3´5´H* gene family at different ripening stages and across cultivars**

Factor: developmental stage
Test: Student-Newman-Keuls
Significance Level: 0.05
Variance: 7.521e-5
Degrees of Freedom: 445
LSD 0.05 = 0.002

Time order	Rank	Developmental stage	Mean	n	Non-significant ranges
1	7	1st preveraison	4.823e-4	83	d
2	8	2nd preveraison	2.798e-4	83	d
3	6	10% veraison	0.002	84	cd
4	5	50% veraison	0.004	84	bc
5	2	100% veraison	0.011	83	a
6	1	1st postveraison	0.011	84	a
7	4	2nd postveraison	0.006	84	b
8	3	harvest	0.007	84	b

Two-way Completely Randomized ANOVA

Cultivar: Aglianico
1st Factor: developmental stage
2nd Factor: gene-copy

Source	df	Type III SS	MS	F	P	
Main effects						
Developmental stage	7	0.020	0.002	10.683	.0000	***
Gene-copy	6	0.083	0.013	50.019	.0000	***
Interaction						
Developmental stage × gene-copy	42	0.092	0.002	7.942	.0000	***
Error	111	0.030	2.785e-4			
Total	166	0.228				
Model	55	0.198	0.003	12.923	.0000	***

R^2 = SSmodel/SStotal = 0.864
Root MSerror = sqrt(MSerror) = 0.016
Mean Y = 0.015
Coefficient of Variation = 109.9%

Compare Means: **Transcript abundance of the *F3´5´H* gene family at different ripening stages in 'Aglianico'**

Factor: developmental stage
Test: Student-Newman-Keuls
Significance Level: 0.05
Variance: 2.785e-4
Degrees of Freedom: 111
LSD 0.05 = 0.010

Time order	Rank	Developmental stage	Mean	n	Non-significant ranges
1	7	1st preveraison	3.246e-4	21	d
2	8	2nd preveraison	2.675e-4	21	d
3	6	10% veraison	0.006	21	cd
4	5	50% veraison	0.013	21	bcd
5	3	100% veraison	0.024	21	ab
6	1	1st postveraison	0.031	21	a
7	4	2nd postveraison	0.017	21	ab
8	2	harvest	0.025	21	ab

Compare Means: **The most expressed duplicate genes in 'Aglianico'**
Factor: gene-copy
Test: Student-Newman-Keuls
Significance Level: 0.05
Variance: 2.785e-4
Degrees of Freedom: 111
LSD 0.05 = 0.009

Rank	Gene-copy	Mean	n	Non-significant ranges
1	*F3´5´Hf*	0.056	24	a
2	*F3´5´Hi*	0.043	24	b
3	*F3´5´Hj*	0.001	24	c
4	*F3´5´Hg*	0.001	24	c
5	*F3´5´Hl*	0.001	24	c
6	*F3´5´Hp*	2.457e-4	24	c
7	*F3´5´Hh*	1.372e-4	23	c

Cultivar: Marzemino
1st Factor: developmental stage
2nd Factor: gene-copy

Source	df	Type III SS	MS	F	P	
Main effects						
Developmental stage	7	0.004	6.607e-4	30.979	.0000	***
Gene-copy	6	0.006	0.001	53.732	.0000	***
Interaction						
Developmental stage × gene-copy	42	0.010	2.523e-4	11.832	.0000	***
Error	112	0.002	2.132e-5			
Total	167	0.024				
Model	55	0.022	4.018e-4	18.840	.0000	***

R^2 = SSmodel/SStotal = 0.902
Root MSerror = sqrt(MSerror) = 0.004
Mean Y = 0.004
Coefficient of Variation = 96.9%

Compare Means: **Transcript abundance of the *F3´5´H* gene family at different ripening stages in ‘Marzemino’**

Factor: developmental stage
Test: Student-Newman-Keuls
Significance Level: 0.05
Variance: 2.132e-5
Degrees of Freedom: 112
LSD 0.05 = 0.002

Time order	Rank	Developmental stage	Mean	n	Non-significant ranges
1	7	1st preveraison	2.436e-4	21	d
2	8	2nd preveraison	1.064e-4	21	d
3	6	10% veraison	0.001	21	d
4	5	50% veraison	0.001	21	d
5	1	100% veraison	0.015	21	a
6	2	1st postveraison	0.010	21	b
7	3	2nd postveraison	0.006	21	c
8	4	harvest	0.001	21	d

Compare Means: **The most expressed duplicate genes in ‘Marzemino’**

Factor: gene-copy
Test: Student-Newman-Keuls
Significance Level: 0.05
Variance: 2.132e-5
Degrees of Freedom: 112
LSD 0.05 = 0.00264150052

Rank	Gene-copy	Mean	n	Non-significant ranges
1	*F3´5´Hj*	0.018	24	a
2	*F3´5´Hi*	0.009	24	b
3	*F3´5´Hf*	0.004	24	c
4	*F3´5´Hg*	7.352e-4	24	d
5	*F3´5´Hl*	3.68e-4	24	d
6	*F3´5´Hp*	9.491e-5	24	d
7	*F3´5´Hh*	9.583e-7	23	d

Cultivar: Grignolino
1st Factor: developmental stage
2nd Factor: gene-copy

Source	df	Type III SS	MS	F	P	
Main effects						
Developmental stage	7	6.278e-5	8.969e-6	9.922	.0000	***
Gene-copy	6	1.583e-4	2.638e-5	29.187	.0000	***
Interaction						
Developmental stage × gene-copy	42	2.112e-4	5.029e-6	5.562	.0000	***
Error	111	1.003e-4	9.040e-7			
Total	166	5.344e-4				
Model	55	4.341e-4	7.893e-6	8.731	.0000	***

R^2 = SSmodel/SStotal = 0.812
Root MSerror = sqrt(MSerror) = 9.507e-4
Mean Y = 8.161e-4
Coefficient of Variation 116.4%

Compare Means: **Transcript abundance of the *F3´5´H* gene family at different ripening stages in 'Grignolino'**

Factor: developmental stage
Test: Student-Newman-Keuls
Significance Level: 0.05
Variance: 9.040e-7
Degrees of Freedom: 111
LSD 0.05 = 5.957e-4

Time order	Rank	Developmental stage	Mean	n	Non-significant ranges
1	4	1st preveraison	6.458e-4	21	b
2	8	2nd preveraison	5.690e-5	21	b
3	6	10% veraison	3.750e-4	21	b
4	5	50% veraison	3.896e-4	21	b
5	2	100% veraison	0.001	20	a
6	1	1st postveraison	0.001	21	a
7	3	2nd postveraison	0.001	21	a
8	7	harvest	2.924e-4	21	b

Compare Means: **The most expressed duplicate genes in ‘Grignolino’**
Factor: gene-copy
Test: Student-Newman-Keuls
Significance Level: 0.05
Variance: 9.040e-7
LSD 0.05 = 5.555e-4

Rank	Gene-copy	Mean	n	Non-significant ranges
1	*F3´5´Hi*	0.002	24	a
2	*F3´5´Hf*	0.002	24	a
3	*F3´5´Hl*	5.789e-4	24	b
4	*F3´5´Hp*	1.812e-4	24	b
5	*F3´5´Hg*	1.598e-4	24	b
6	*F3´5´Hj*	9.554e-5	24	b
7	*F3´5´Hh*	1.834e-5	23	b

Cultivar: Nebbiolo
1st Factor: developmental stage
2nd Factor: gene-copy

Source	df	Type III SS	MS	F	P	
Main effects						
Developmental stage	7	2.601e-5	3.717e-6	6.902	.0000	***
Gene-copy	6	1.182e-4	1.970e-5	36.582	.0000	***
Interaction						
Developmental stage × gene-copy	42	1.564e-4	3.723e-6	6.914	.0000	***
Error	111	5.977e-5	5.385e-7			
Total	166	3.604e-4				
Model	55	3.006e-4	5.466e-6	10.151	.0000	***

R^2 = SSmodel/SStotal = 0.834
Root MSerror = sqrt(MSerror) = 7.338e-4
Mean Y = 6.912e-4
Coefficient of Variation = 106.1%

Compare Means: **Transcript abundance of the *F3´5´H* gene family at different ripening stages in 'Nebbiolo'**

Factor: developmental stage
Test: Student-Newman-Keuls
Significance Level: 0.05
Variance: 5.385e-7
Degrees of Freedom: 111
LSD 0.05 = 4.598e-4

Time order	Rank	Developmental stage	Mean	n	Non-significant ranges
1	4	1st preveraison	7.268e-4	20	b
2	5	2nd preveraison	6.880e-4	21	b
3	6	10% veraison	3.929e-4	21	b
4	3	50% veraison	7.309e-4	21	b
5	1	100% veraison	0.001	21	a
6	2	1st postveraison	9.205e-4	21	b
7	7	2nd postveraison	2.675e-4	21	b
8	8	harvest	2.601e-4	21	b

Compare Means: **The most expressed duplicate genes in 'Nebbiolo'**
Factor: gene-copy
Test: Student-Newman-Keuls
Significance Level: 0.05
Variance: 5.385e-7
LSD 0.05 = 4.288e-4

Rank	Gene-copy	Mean	n	Non-significant ranges
1	*F3´5´Hi*	0.002	24	a
2	*F3´5´Hf*	0.001	24	b
3	*F3´5´Hj*	5.920e-4	23	c
4	*F3´5´Hl*	1.531e-4	24	c
5	*F3´5´Hp*	1.322e-4	24	c
6	*F3´5´Hg*	9.279e-5	24	c
7	*F3´5´Hh*	1.666e-7	23	c

Two-way Completely Randomized ANOVA: gene-copy vs. developmental stage, across cultivars

Gene-copy: *F3´5´Hf*

1st Factor: cultivar
2nd Factor: developmental stage

Source	df	Type III SS	MS	F	P	
Main effects						
Cultivar	3	0.052	0.017	114.036	.0000	***
Developmental stage	7	0.017	0.002	16.464	.0000	***
Interaction						
Cultivar × developmental stage	21	0.047	0.002	14.740	.0000	***
Error	64	0.009	1.522e-4			
Total	95	0.126				
Model	31	0.116	0.003	24.739	.0000	***

R^2 = SSmodel/SStotal = 0.922
Root MSerror = sqrt(MSerror) = 0.012
Mean Y = 0.016
Coefficient of Variation = 76.7%

Compare Means
Factor: developmental stage
Test: Student-Newman-Keuls
Significance Level: 0.05
Variance: 1.522e-4
Degrees of Freedom: 64
LSD 0.05 = 0.010

Time order	Rank	Developmental stage	Mean	n	Non-significant ranges
1	7	1st preveraison	0.001	12	d
2	8	2nd preveraison	0.001	12	d
3	6	10% veraison	0.002	12	d
4	5	50% veraison	0.010	12	cd
5	3	100% veraison	0.025	12	b
6	4	1st postveraison	0.019	12	bc
7	2	2nd postveraison	0.026	12	b
8	1	harvest	0.040	12	a

Gene-copy: ***F3´5´Hg***

1st Factor: cultivar
2nd Factor: developmental stage

Source	df	Type III SS	MS	F	P	
Main effects						
Cultivar	3	5.133e-5	1.711e-5	86.586	.0000	***
Developmental stage	7	2.893e-5	4.134e-6	20.919	.0000	***
Interaction						
Cultivar × developmental stage	21	3.194e-5	1.521e-6	7.697	.0000	***
Error	64	1.264e-5	1.976e-7			
Total	95	1.248e-4				
Model	31	1.122e-4	3.620e-6	18.317	.0000	***

R^2 = SSmodel/SStotal = 0.898
Root MSerror = sqrt(MSerror) = 4.445e-4
Mean Y = 7.260e-4
Coefficient of Variation = 61.2%

Compare Means
Factor: developmental stage
Test: Student-Newman-Keuls
Significance Level: 0.05
Variance: 1.976e-7
Degrees of Freedom: 64
LSD 0.05 = 3.625e-4

Time order	Rank	Developmental stage	Mean	n	Non-significant ranges
1	8	1st preveraison	7.1e-5	12	c
2	7	2nd preveraison	7.941e-5	12	c
3	6	10% veraison	2.875e-4	12	c
4	5	50% veraison	4.169e-4	12	c
5	4	100% veraison	9.89e-4	12	b
6	2	1st postveraison	0.001	12	a
7	1	2nd postveraison	0.001	12	a
8	3	harvest	0.001	12	b

Gene-copy: *F3´5´Hh*

1st Factor: cultivar
2nd Factor: developmental stage

Source	df	Type III SS	MS	F	P	
Main effects						
Cultivar	3	1.024e-6	3.414e-7	64.958	.0000	***
Developmental stage	7	5.547e-7	7.925e-8	15.079	.0000	***
Interaction						
Cultivar × developmental stage	21	1.377e-6	6.561e-8	12.483	.0000	***
Error	63	3.311e-7	5.255e-9<-			
Total	94	3.294e-6				
Model	31	2.963e-6	9.559e-8	18.187	.0000	***

R^2 = SSmodel/SStotal = 0.899
Root MSerror = sqrt(MSerror) = 7.249e-5
Mean Y = 6.681e-5
Coefficient of Variation =108.5%

Compare Means
Factor: developmental stage
Test: Student-Newman-Keuls
Significance Level: 0.05
Variance: 5.255e-9
Degrees of Freedom: 63
LSD 0.05 = 6.177e-5

Time order	Rank	Developmental stage	Mean	n	Non-significant ranges
1	7	1st preveraison	1.25e-6	12	c
2	8	2nd preveraison	0	12	c
3	5	10% veraison	6.416e-6	12	c
4	6	50% veraison	5.416e-6	12	c
5	3	100% veraison	1.247e-4	11	b
6	1	1st postveraison	2.079e-4	12	a
7	2	2nd postveraison	1.492e-4	12	ab
8	4	harvest	4.433e-5	12	c

Gene-copy: ***F3´5´Hi***

1st Factor: cultivar
2nd Factor: developmental stage

Source	df	Type III SS	MS	F	P	
Main effects						
Cultivar	3	0.0279	0.009	26.883	.0000	***
Developmental stage	7	0.0196	0.002	8.116	.0000	***
Interaction						
Cultivar × developmental stage	21	0.0340	0.001	4.683	.0000	***
Error	64	0.022	3.464e-4			
Total	95	0.103				
Model	31	0.081	0.002	7.607	.0000	***

R^2 = SSmodel/SStotal = 0.786
Root MSerror = sqrt(MSerror) = 0.018
Mean Y = 0.014
Coefficient of Variation = 128.3%

Compare Means
Factor: developmental stage
Test: Student-Newman-Keuls
Significance Level: 0.05
Variance: 3.464e-4
Degrees of Freedom: 64
LSD 0.05 = 0.015

Time order	Rank	Developmental stage	Mean	n	Non-significant ranges
1	7	1st preveraison	0.001	12	b
2	8	2nd preveraison	6.404e-4	12	b
3	4	10% veraison	0.010	12	b
4	3	50% veraison	0.015	12	b
5	2	100% veraison	0.032	12	a
6	1	1st postveraison	0.042	12	a
7	5	2nd postveraison	0.007	12	b
8	6	harvest	0.005	12	b

Gene-copy: *F3´5´Hj*

1st Factor: cultivar
2nd Factor: developmental stage

Source	df	Type III SS	MS	F	P	
Main effects						
Cultivar	3	0.005	0.001	75.048	.0000	***
Developmental stage	7	0.003	4.724e-4	19.392	.0000	***
Interaction						
Cultivar × developmental stage	21	0.008	3.834e-4	15.737	.0000	***
Error	63	0.001	2.436e-5			
Total	94	0.018				
Model	31	0.016	5.457e-4	22.399	.0000	***

R^2 = SSmodel/SStotal = 0.916
Root MSerror = sqrt(MSerror) = 0.004
Mean Y = 0.005
Coefficient of Variation = 93.5%

Compare Means
Factor: developmental stage
Test: Student-Newman-Keuls
Significance Level: 0.05
Variance: 2.436e-5
Degrees of Freedom: 63
LSD is: LSD 0.05 = 0.004

Time order	Rank	Developmental stage	Mean	n	Non-significant ranges
1	7	1st preveraison	3.564e-4	11	c
2	8	2nd preveraison	9.016e-5	12	c
3	5	10% veraison	0.001	12	bc
4	6	50% veraison	0.001	12	bc
5	1	100% veraison	0.016	12	a
6	2	1st postveraison	0.012	12	a
7	3	2nd postveraison	0.006	12	b
8	4	harvest	0.002	12	bc

Gene-copy: *F3´5´Hl*

1st Factor: cultivar
2nd Factor: developmental stage

Source	df	Type III SS	MS	F	P	
Main effects						
Cultivar	3	2.303e-5	7.677e-6	78.780	.0000	***
Developmental stage	7	4.413e-5	6.304e-6	64.693	.0000	***
Interaction						
Cultivar × developmental stage	21	3.106e-5	1.479e-6	15.178	.0000	***
Error	64	6.237e-6	9.745e-8			
Total	95	1.044e-4				
Model	31	9.823e-5	3.168e-6	32.514	.0000	***

R^2 = SSmodel/SStotal = 0.940
Root MSerror = sqrt(MSerror) = 3.121e-4
Mean Y = 6.358e-4
Coefficient of Variation = 49.0%

Compare Means
Factor: developmental stage
Test: Student-Newman-Keuls
Significance Level: 0.05
Variance: 9.745e-8
LSD 0.05 = 2.546e-4

Time order	Rank	Developmental stage	Mean	n	Non-significant ranges
1	7	1st preveraison	3.283e-5	12	d
2	8	2nd preveraison	2.916e-6	12	d
3	5	10% veraison	2.269e-4	12	d
4	6	50% veraison	2.127e-4	12	d
5	4	100% veraison	5.32e-4	12	c
6	2	1st postveraison	0.001	12	b
7	1	2nd postveraison	0.002	12	a
8	3	harvest	6.073e-4	12	c

Gene-copy: *F3´5´Hp*

1st Factor: cultivar
2nd Factor: developmental stage

Source	df	Type III SS	MS	F	P	
Main effects						
Cultivar	3	9.066e-8	3.022e-8	4.027	.0110	*
Developmental stage	7	3.301e-7	4.716e-8	6.285	.0000	***
Interaction						
Cultivar × developmental stage	21	3.814e-7	1.816e-8	2.421	.0037	**
Error	63	4.727e-7	7.503e-9			
Total	94	1.291e-6				
Model	31	8.183e-7	2.639e-8	3.518	.0000	***

R^2 = SSmodel/SStotal = 0.633
Root MSerror = sqrt(MSerror) = 8.662e-5
Mean Y = 1.364e-4
Coefficient of Variation 63.5%

Compare Means
Factor: developmental stage
Test: Student-Newman-Keuls
Significance Level: 0.05
Variance: 7.503e-9
Degrees of Freedom: 63
LSD 0.05 = 7.380e-5

Time order	Rank	Developmental stage	Mean	n	Non-significant ranges
1	1	1st preveraison	2.459e-4	12	a
2	6	2nd preveraison	8.1e-5	11	bcd
3	8	10% veraison	5.091e-5	12	d
4	7	50% veraison	7.625e-5	12	cd
5	1	100% veraison	1.6566e-4	12	abc
6	2	1st postveraison	1.81e-4	12	ab
7	4	2nd postveraison	1.538e-4	12	abc
8	5	harvest	1.320e-4	12	bcd
