# Supplementary material for: Expansion and subfunctionalisation of flavonoid 3',5'-hydroxylases in the grapevine lineage
Source: BMC Genomics. 2010 Oct 12;11:562. doi: 10.1186/1471-2164-11-562 (PMC3091711; doi:10.1186/1471-2164-11-562)
Supplement: Additional file 13 — Primer sequences for grapevine F3'Hs and F3'5'Hs. [file 1471-2164-11-562-S13.PDF]

Additional file 13 – Primer sequences for grapevine *F3´H*s and *F3´5´H*s

Gene	forward 5´-3´	reverse 5´-3´
F3´Ha	GGCGGAAGGTTTCCTTGAT	GCACGTTGATCTCGGTGAG
F3´Hb	GGCGGAAGGTTTCCTTGAC	GCACGTTGATCTCGCTGAA
F3´5´Hf	TGTACCAACGACCCCAAAAT	GAACCTTCCTCGTGTCTCAG
F3´5´Hg	AACGTCCCTAAAATGTACTCAACC	GAACCTTCCTCGTGTCTCAAA
F3´5´Hh	CGGGCTTTCCCAGACATT	GCAGGAACAGACACTTCATCG
F3´5´Hi	GCCAGAGACCACTCGATTAC	ACCCAGATTTTCTGGACGTG
F3´5´Hj	CAGAAAATCTGGGTTTCCCTTT	TTCGAGCCATGCTTGAGTTA
F3´5´Hl	GGCTTGTAGGTATGGAAGTTTTT	GTAGTCATGGGCCATCAAGG
F3´5´Hm, -n	CACGCTGAGTCTAGTGTTCTCC	GGATCATGTGATTGGAAGGAA
F3´5´Ho	CCATCTTATTGCCGGAACAG	CGAAGATTTCGCACCAGAG
F3´5´Hp	TTCCAGTCAAATGAATGTACCAG	TTGAGCGAAAAGAATGCAAG
