# Supplementary material for: Expansion and subfunctionalisation of flavonoid 3',5'-hydroxylases in the grapevine lineage
Source: BMC Genomics. 2010 Oct 12;11:562. doi: 10.1186/1471-2164-11-562 (PMC3091711; doi:10.1186/1471-2164-11-562)
Supplement: Additional file 14 — Berry sampling in four red-skinned cultivars and a green-skinned cultivar (Tocai) across eight developmental stages. [file 1471-2164-11-562-S14.PDF]

Additional file 14 – Berry sampling in four red-skinned cultivars and a green-skinned cultivar (Tocai) across eight developmental stages

Berry weight, sugar content (Brix°), pH, titratable acidity (TA), and % of coloured berries at the time of sampling are indicators of the ripening stage.

Date	Treatment	Sampling	Developmental stage	Weight (g)	Brix°	pH	TA	veraison
16-Jul-2008	Aglianico taurasi VCR23	1	1st pre veraison	1.30	2.80	2.45	32.10	0%
16-Jul-2008	Marzemino VCR3	1	1st pre veraison	0.98	2.80	2.41	39.15	0%
28-Jul-2008	Grignolino R1	1	1st pre veraison	1.16	3.30	2.38	37.35	0%
16-Jul-2008	Nebbiolo R1	1	1st pre veraison	0.86	3.40	2.39	39.00	0%
16-Jul-2008	Tocai VCR33	1	1st pre veraison	0.65	3.00	2.53	32.25	0%
25-Jul-2008	Aglianico taurasi VCR23	2	2nd pre veraison	1.33	3.50	2.39	35.33	0%
25-Jul-2008	Marzemino VCR3	2	2nd pre veraison	1.05	4.00	2.31	45.60	0%
4-Aug-2008	Grignolino R1	2	2nd pre veraison	1.49	4.10	2.41	41.25	0%
25-Jul-2008	Nebbiolo R1	2	2nd pre veraison	0.92	3.90	2.26	45.45	0%
25-Jul-2008	Tocai VCR33	2	2nd pre veraison	0.83	3.90	2.49	36.60	0%
13-Aug-2008	Aglianico taurasi VCR23	3 (green)	10% veraison	1.95	4.90	2.35	39.75	0%
4-Aug-2008	Marzemino VCR3	3 (green)	10% veraison	1.21	4.00	2.41	39.45	0%
8-Aug-2008	Grignolino R1	3 (green)	10% veraison	1.28	4.90	2.44	40.35	0%
4-Aug-2008	Nebbiolo R1	3 (green)	10% veraison	1.08	4.50	2.22	48.90	0%
31-Jul-2008	Tocai VCR33	3 (firm)	10% veraison	0.82	4.00	2.52	34.95	0%
13-Aug-2008	Aglianico taurasi VCR23	4 (red)	10% veraison	1.62	8.80	2.43	35.25	35%
4-Aug-2008	Marzemino VCR3	4 (red)	10% veraison	1.23	7.90	2.50	35.70	17%
8-Aug-2008	Grignolino R1	4 (red)	10% veraison	1.50	11.30	2.62	30.00	10%
4-Aug-2008	Nebbiolo R1	4 (red)	10% veraison	0.87	10.70	2.30	42.30	7%
31-Jul-2008	Tocai VCR33	4 (soft)	10% veraison	0.99	7.90	2.62	29.40	15%
18-Aug-2008	Aglianico taurasi VCR23	5 (green)	50% veraison	2.00	5.00	2.38	39.60	0%
7-Aug-2008	Marzemino VCR3	5 (green)	50% veraison	1.31	4.70	2.48	37.05	0%
13-Aug-2008	Grignolino R1	5 (green)	50% veraison	1.43	6.00	2.46	38.55	0%
13-Aug-2008	Nebbiolo R1	5 (green)	50% veraison	1.00	5.90	2.23	41.40	0%
4-Aug-2008	Tocai VCR33	5 (firm)	50% veraison	0.82	4.80	2.52	33.60	0%
18-Aug-2008	Aglianico taurasi VCR23	6 (red)	50% veraison	1.85	10.00	2.50	31.20	61%
7-Aug-2008	Marzemino VCR3	6 (red)	50% veraison	1.42	8.90	2.64	27.45	32%
13-Aug-2008	Grignolino R1	6 (red)	50% veraison	1.78	10.80	2.62	28.05	43%
13-Aug-2008	Nebbiolo R1	6 (red)	50% veraison	1.18	12.30	2.43	27.00	53%
4-Aug-2008	Tocai VCR33	6 (soft)	50% veraison	1.23	10.00	2.73	22.20	39%
25-Aug-2008	Aglianico taurasi VCR23	7 (red)	100% veraison	2.20	11.90	2.57	26.81	86%
21-Aug-2008	Marzemino VCR3	7 (red)	100% veraison	1.82	14.00	2.97	13.95	100%
25-Aug-2008	Grignolino R1	7 (red)	100% veraison	2.08	13.40	2.80	19.50	93%
21-Aug-2008	Nebbiolo R1	7 (red)	100% veraison	1.41	13.70	2.69	20.25	97%
18-Aug-2008	Tocai VCR33	7 (soft)	100% veraison	1.65	14.70	3.08	10.35	100%
4-Sep-2008	Aglianico taurasi VCR23	8	1st post veraison	2.32	14.00	2.74	18.92	100%
1-Sep-2008	Marzemino VCR3	8	1st post veraison	2.11	16.60	3.13	9.56	100%
1-Sep-2008	Grignolino R1	8	1st post veraison	2.15	16.60	2.89	15.60	100%
1-Sep-2008	Nebbiolo R1	8	1st post veraison	1.58	18.80	2.72	14.04	100%
25-Aug-2008	Tocai VCR33	8	1st post veraison	1.66	15.60	3.16	10.43	100%
15-Sep-2008	Aglianico taurasi VCR23	9	2nd post veraison	2.52	16.50	2.89	13.88	100%
9-Sep-2008	Marzemino VCR3	9	2nd post veraison	1.97	18.80	3.20	7.65	100%
9-Sep-2008	Grignolino R1	9	2nd post veraison	2.24	17.80	3.00	12.90	100%
15-Sep-2008	Nebbiolo R1	9	2nd post veraison	1.76	21.10	2.90	9.98	100%
1-Sep-2008	Tocai VCR33	9	2nd post veraison	1.58	17.80	3.21	8.97	100%
1-Oct-2008	Aglianico taurasi VCR23	10	Harvest	2.38	18.00	2.86	12.98	100%
22-Sep-2008	Marzemino VCR3	10	Harvest	2.33	19.00	3.29	7.20	100%
22-Sep-2008	Grignolino R1	10	Harvest	2.26	18.50	3.07	10.80	100%
1-Oct-2008	Nebbiolo R1	10	Harvest	1.71	22.80	2.88	9.38	100%
9-Sep-2008	Tocai VCR33	10	Harvest	1.78	20.00	3.39	6.45	100%
