# Supplementary material for: Expansion and subfunctionalisation of flavonoid 3',5'-hydroxylases in the grapevine lineage
Source: BMC Genomics. 2010 Oct 12;11:562. doi: 10.1186/1471-2164-11-562 (PMC3091711; doi:10.1186/1471-2164-11-562)
Supplement: Additional file 10 — Transcripts of duplicate F3'5'Hs detected in various organs of two grape cultivars by semiquantitative PCR. Bold + indicates high expression of PCR amplicons visualised on agarose gel stained with ethidium bromide (see Figure 7), regular + indicates weak expression detected only by the more sensitive silver staining, - indicates lack of detectable transcripts. [file 1471-2164-11-562-S10.PDF]

Additional file 10 - Transcripts of duplicate *F3´5´H*s detected in various organs of two grape cultivars by semiquantitative PCR

Bold + indicates high expression of PCR amplicons visualised on agarose gel stained with ethidium bromide (see Figure 7), regular + indicates weak expression detected only by the more sensitive silver staining, - indicates lack of detectable transcripts.

	Merlot								Aglianico							
	gDNA	leaf	petiole	tendril	flower	shoot	skin	flesh	gDNA	leaf	petiole	tendril	flower	shoot	skin	flesh
F3'5'Hf	**+**	-	+	+	+	-	**+**	+	**+**	+	+	-	+	+	**+**	-
F3'5'Hg	**+**	-	-	-	-	-	**+**	-	**+**	+	+	-	+	-	**+**	-
F3'5'Hi	**+**	+	+	+	-	-	**+**	-	**+**	+	+	-	+	-	**+**	-
F3'5'Hj	**+**	-	+	+	-	+	**+**	-	**+**	+	+	-	+	-	**+**	-
F3'5'Hl	**+**	-	+	-	+	-	**+**	-	**+**	-	+	-	+	-	**+**	-
F3'5'Hm, -n	**+**	-	**+**	-	**+**	-	-	-	**+**	-	-	-	-	-	-	-
F3'5'Ho	**+**	-	**+**	-	**+**	-	-	-	**+**	-	-	-	-	-	-	-
F3'5'Hp	**+**	**+**	+	**+**	**+**	**+**	+	-	**+**	**+**	**+**	**+**	**+**	**+**	+	-
